# Risk factors for breast cancer: an umbrella review of observational cohort studies and causal relationship analysis

**DOI:** 10.3389/fonc.2025.1541233

**Published:** 2025-05-02

**Authors:** Zhuo Wang, Lei Feng, Yuqing Xia, Zheming Zhu, Lina Wu, Song Gao

**Affiliations:** Department of Oncology, Shengjing Hospital of China Medical University, Shenyang, China

**Keywords:** breast cancer, etiology, cohort studies, umbrella review, meta-analysis

## Abstract

**Objective:**

To conduct an umbrella review of prospective meta-analyses and perform a causal relationship analysis to evaluate causal effects.

**Methods:**

PubMed, Web of Science, Embase, and manual reference list searches were used from database inception to July 27, 2023. Meta-analyses of prospective studies on non-genetic risk factors for breast cancer incidence were included. Overlapping articles were assessed using corrected coverage area. We utilized the AMSTAR-2 criteria to evaluate methodological quality and graded each meta-analysis to assess the strength of evidence. This study is registered with PROSPERO (CRD42023470151). We further explored the causal impacts.

**Results:**

Risk factors were classified into 11 categories. Among the 281 meta-analyses of cohort studies, five (1.8%) provided strong evidence, eight (2.8%) indicated highly suggestive evidence, and 23 (8.2%) and 55 (19.6%) showed suggestive and weak evidence, respectively. Breast density (2.89; 2.57-3.25), cardiac glycoside (1.39; 1.33-1.45), atrial fibrillation (1.18; 1.14-1.22), vegetable-fruit-soybean dietary pattern (0.87; 0.83-0.92), and postmenopausal women with BMI ≥25 (0.86; 0.81-0.91) were strongly associated with breast cancer incidence. For all associations graded as weak evidence or higher, further confirmed the causal relationship between BMI, fruit intake, calcium channel blockers, cheese intake, insulin like growth factor-1 levels, serum triglyceride levels causally

**Discussion:**

Identifying primary risk factors is crucial for delineating high-risk populations among women, facilitating tailored prevention strategies and advancing investigations into underlying mechanisms.

**Systematic review registration:**

https://www.crd.york.ac.uk/PROSPERO/, identifier CRD42023470151.

## 1 Introduction

By 2023, breast cancer (BC) accounted for 11.6% of all cancer diagnoses worldwide, with 2.3 million new cases representing 31% of diagnoses in women. It is the second most common cancer globally, following lung cancer, and the most common cancer among women (1, 2). Despite medical advances, BC incidence continues to rise (1). Identifying and mitigating risk factors is crucial to addressing the growing BC burden. Genetic factors such as BRCA gene mutations contribute to BC occurrence (3) but offer limited preventive value. Recent research has highlighted modifiable factors, such as environmental and lifestyle influences, that impact BC risk (4–8). Mitigating these non-genetic risk factors is crucial for lowering BC incidence. However, owing to the impracticality of studying these exposures through randomized controlled trials, these studies may introduce inherent biases, including selection bias (i.e., inappropriate participant selection) and information bias (i.e., inaccurate data collection), which could lead to either an overestimation or underestimation of the results. Furthermore, wide effect size ranges, and even conflicts often exhibited. For example, Kast et al. (9) reported that a higher BMI in young adults is associated with a reduced risk of BC. In contrast, Fakhri et al. (10) found that obese women have a higher risk of BC compared to those with a BMI below 30. In this context, meta-analysis is a useful tool to address studies with varying effect sizes and directions.

Nevertheless, recently, as original research continuously updates and more meta-analyses emerge, there are obvious discrepancies in findings, even within the same topic. For instance, two recent meta-analyses investigating the relationship between atrial fibrillation (AF) and breast cancer (BC) incidence reached entirely contradictory conclusions (11, 12). The umbrella review effectively evaluate these diverse results, combining systematic reviews and meta-analyses on a given topic, assessing sample size, association strength, heterogeneity, and bias.

However, establishing causality is challenging with observational research. Mendelian randomization (MR) uses genetic variation as a proxy for exposure, reducing confounding factors and enhancing causal inference (13, 14). Pearson-Stuttard et al. (15) explored the risk of developing multiple-site cancers with type 2 diabetes using an umbrella review and MR, exemplifying effective methodology.

Therefore, we aimed to conduct an umbrella review to explore BC risk factors and perform a MR analysis to evaluate the causal effects.

## 2 Materials and methods

### 2.1 Literature search and selection criteria

We conducted a comprehensive search using keywords across PubMed, Web of Science, and Embase databases. Our search encompassed meta-analyses that examined the association between non-genetic risk factors for BC from database inception to July 27, 2023. **Supplementary Table 1** outlines the complete search strategy. We also manually reviewed reference lists of eligible studies.

This study followed the Preferred Reporting Items for Systematic Reviews and Meta-analyses (PRISMA) reporting guideline (16) and was registered at PROSPERO (https://www.crd.york.ac.uk/PROSPERO/) with CRD42023470151.

Two authors independently searched the databases, screened titles and abstracts, and identified meta-analyses meeting the inclusion criteria through full-text reading. A third author resolved any discrepancies. Inclusion criteria involved meta-analyses with: 1. observational cohort study designs; 2. non-genetic risk factors as the exposure of interest; 3. BC as the reported outcome; 4. available risk estimates between risk factors and BC (risk ratio, odds ratio, hazard ratio) with 95% confidence intervals (CIs), number of cases/controls, or total population size; 5. publication in English; and 6. study population comprising women. We excluded meta-analyses of genetic markers, systematic reviews without quantitative analyses, animal or laboratory studies, and reviews lacking study-specific data (risk ratio, odds ratio, hazard ratio) that could not be retrieved from the original studies. We also excluded studies with baseline populations already diagnosed with cancer.

### 2.2 Overlapping and outdated reviews

When two or more reviews examine the same exposure and outcome, overlapping associations may result in multiple primary studies being included in the reviews. Additionally, research indicates that half of published reviews become outdated within 5.5 years. We first identified meta-analyses with identical risk factors and outcomes to mitigate bias in interpreting such outcomes. Subsequently, we:

1. Selected the most recent literature for reviews with the same author.
2. Excluded outdated overlapping reviews published before 2018 with different authors.
3. Assessed the extent of overlap using a generated graphical cross-table (citation matrix) for reviews published before or after 2018 with different authors and used an index termed corrected coverage area (CCA) to quantify the degree of overlap, calculated as CCA = (N - r)/(r * c - r) * 100%, where N: the total number of primary studies across all reviews, r: rows, and c: columns, expressed as a percentage.

Overlap was categorized as follows: 1. very high: CCA >15%, 2. high: CCA=11-15%, 3. moderate: CCA=6-10%, and 4. slight: CCA=0-5% (17). Both reviews were retained for slight or moderate overlap. For high or very high overlap, the review with the highest quality based on the AMSTAR 2 tool was prioritized. The most recent review was included when quality ratings were identical (18, 19).

### 2.3 Data extraction

Two reviewers independently extracted data, including the first author’s name, journal name, publication year, study design, exposure factors, health outcomes, included studies, cases, total participants, and the estimated summary effects with 95% CIs, from eligible studies. Additionally, if available, information relevant to dose-response relationships was retrieved from meta-analyses. To ensure transparency, a third author reviewed the discrepancies between the two reviewers and resolved them by considering all relevant data and methodologies.

### 2.4 Evaluation of the quality of included meta-analyses

Two reviewers (WZ and XYQ) evaluated the methodological quality of the included studies using the AMSTAR-2 tool (20), assigning an overall score. In case of disagreement, a third author (GS) was consulted. AMSTAR-2 assesses 16 items, with 7 considered critical. Deficiencies in any critical item may affect the overall review quality. The key areas deemed crucial included: protocol registered before the commencement of the review; adequacy of the literature search; justification for excluding individual studies; risk of bias in the included studies; appropriateness of meta-analytical methods; considering bias risk when interpreting results; and assessment of the presence and likely impact of publication bias. The final ratings were classified into four levels ranging from “high” to “very low”: 1. high: no defects or one non-critical area with defects; 2. moderate: more than one non-critical area with defects; 3. low: one critical area with or without non-critical areas with defects and 4. very low: more than one critical area with or without non-critical areas with defects. AMSTAR-2 scoring results assess the quality of the included studies and account for potential biases and methodological limitations, providing a more reliable interpretation of results.

### 2.5 Evaluating the strength of evidence using grading criteria

BC risk factors were categorized into four evidence-based classes: class I (strong evidence), class II (highly suggestive evidence), class III (suggestive evidence), and class IV (weak evidence) (21, 22) (**Table 1**). This classification system offers an objective, standardized approach, consistent with other grading schemes in cancer epidemiology. This classification method enables the study to rank the strength of evidence for each risk factor. class I and II evidence correspond to factors with a high level of confidence, whereas class III and IV represent factors with lower confidence. Specifically, class IV (weak evidence) factors are associated with reduced confidence, which may be influenced by factors such as data heterogeneity, insufficient sample size, or methodological limitations.

**Table 1:** Evidence grading for meta-analyses of risk factors associated with breast cancer incidence (cohort studies only).

Evidence	Criteria used	Decreased Risk	Increased Risk
Strong	p < 10^–6^; >1000 cases; I^2^ < 50%; no small study effects; prediction interval excludes the null value; no excess significance bias	***Dietary intake*** Vegetable-fruit-soybean dietary pattern: highest vs. lowest; ***Anthropometric indices*** BMI>=25: high vs. low, postmenopausal;	***Imageological diagnosis*** Breast density: highest vs. lowest category; ***Use of medical/hormonal therapy*** Cardiac glycosides use: ever vs. never; ***Pre-existing medical conditions and interventions*** Atrial fibrillation: ever vs. never;
Highly Suggestive	p < 10^–6^; >1000 cases; p < 0.05 of the largest study in a meta-analysis	***Life behaviour*** Physical activity: highest vs. lowest category; ***Anthropometric indices*** BMI iya:per 5 kg/m2;	***Life behaviour*** Education level: highest vs. lowest category; Smoking:ever vs. never; light exposure at night: highest vs. lowest category; ***Use of medical/hormonal therapy*** Antipsychotic use: ever vs. never; Calcium channel blockers: ever vs. never; antibiotic use: ever vs. never;
Suggestive	p < 10^–3^; >1000 cases	***Dietary intake*** Fruit intake: per 100 g/day; Fiber intake: highest vs. lowest category; Selenium: ever vs. never; Tofu intake: highest vs. lowest category; Adherence score: highest vs. lowest category; ***Life behaviour*** Lifestyle Quality Indices: highest vs. lowest category; ***Anthropometric indices*** BMI: per 5 kg/m2; BMI<25:high vs. low, postmenopausal; ***Use of medical/hormonal therapy*** Aspirin intake: ever vs. never; ***Pre-existing medical conditions and interventions*** Bariatric Surgery: ever vs. never;	***Dietary intake*** Alcohol: highest vs. lowest category; Total meat intake: per 100 g/day; Red meat intake: per 100 g/day; ***Imageological diagnosis*** Bone mineral density: highest vs. lowest category; ***Life behaviour*** Famine exposure: ever vs. never; ***Anthropometric indices*** BMI: highest vs. lowest category; Fat mass: highest vs. lowest category; Weight gain: highest vs. lowest category; ***Biomarkers*** IGF1: highest vs. lowest category; ***Pre-existing medical conditions and interventions*** Antibody: ever vs. never; Periodontal disease: ever vs. never; Metabolic Syndrome: ever vs. never; Hyperthyroidism: ever vs. never;
Weak	p < 0.05	***Dietary intake*** Vegetable intake: per 100 g/day; Soy intake: per 30 g/day; Soy isoflavone: per 10mg/day; Coffee intake: highest vs. lowest category; Vitamin D intake: highest vs. lowest category; Cheese intake:per 30 g/day; B-carotene:per 5000ug/day; Flavonols: highest vs. lowest category; Dietary calcium intake: per 350mg/day; Dietary folate intake: highest vs. lowest category; Prudent/healthy dietary pattern: highest vs. lowest category; Fruit intake: highest vs. lowest category; Adherence score: per 1-point; A-carotene: highest vs. lowest category; Vitamin B2: highest vs. lowest category; Fruits and vegetables intake: highest vs. lowest category; Total dairy food intake: highest vs. lowest category; Dietary calcium intake: highest vs. lowest category; Marine n-3 PUFA: highest vs. lowest category; Vegetarians: yes vs. no; Higher Mushroom Consumption: highest vs. lowest category; ***Life behaviour*** Physical activity at a young age: highest vs. lowest category; Time in the Sun: highest vs. lowest category; ***Past gynaecological history*** Parity: parous vs. nulliparous; ***Anthropometric indices*** Weight loss: highest vs. lowest category; ***Use of medical/hormonal therapy*** Bisphosphonates: ever vs. never; Thiazolidinediones use: ever vs. never; Insulins: ever vs. never; ***Biomarkers*** Serum TG levels: highest vs. lowest category; ***Pre-existing medical conditions and*** ***interventions*** CAD: ever vs. never; ***congenital factor*** Twin membership: highest vs. lowest category;	***Dietary intake*** DII: per 1-point; Wine Drinking: highest vs. lowest category; Processed meat intake: per 50 g/day; SSBs: per 250mg/day; Processed meat intake: per 50 g/day; Glycemic index/Glycemic load: highest vs. lowest category; Glycemic index: highest vs. lowest category; Glycemic index: per 10 units/day; Total fat intake: highest vs. lowest category; ***Life behaviour*** Negative Emotions: ever vs. never; Flight attendants: yes vs. no; Sedentary work: highest vs. lowest category; Occupational exposure-organic solvents: ever vs. never; ***Environment:*** NO2: per 10 ug/m3; ***Anthropometric indices*** Birth length: highest vs. lowest category; BMI>= 30: ever vs. never; Birth Weight: highest vs. lowest category; ***Biomarkers*** Serum/plasma iron: highest vs. lowest category; Plasma prolactin levels: highest vs. lowest category; ***Use of medical/hormonal therapy*** Antidepressant use: ever vs. never; ***Pre-existing medical conditions and*** ***interventions*** Obstructive sleep apnea: ever vs. never; Autoimmune thyroiditis: ever vs. never; Goitre: ever vs. never; Sleep-disordered breathing: ever vs. never; Diabetes: ever vs. never; ***congenital factor*** Paternal age: per 15 years;

BMI, body mass index; BMI iya, Body mass index in young adulthood; CAD, coronary artery disease; outdoor LAN, outdoor light at night. The bold text represents the main categories of the risk factors.

### 2.6 Data analysis

We focused on cohort studies, recalculating summary effects and 95% CIs using random- or fixed-effects models (23). Heterogeneity was evaluated using the I² statistic and 95% predicted intervals (PIs) (24–26) to account for variability across studies and reduce its impact on the results. We evaluated the presence of publication bias by assessing small-study effects and excess significance bias. Egger’s regression asymmetry test (P<.10) and whether the summary estimate of random effects exceeded the point estimate of the largest study in the meta-analysis were used to assess small-study effects (27). Additionally, sensitivity analyses were conducted on all eligible cohort and case-control studies using the same criteria as those used in the primary analysis. R software version 4.3.0 was used for all statistical analyses (http://cran.r-project.org/).

### 2.7 MR

We performed a two-sample MR analysis using genetic variants as proxies for exposure to explore the causal impacts of non-genetic risk factors on BC (28) Genome-wide association study (GWAS) catalogs were queried for exposures rated as weak evidence or higher in the umbrella review to find relevant GWAS offering summary-level genetic information (29). We used data from previous GWAS to ascertain the association between single nucleotide polymorphisms (SNPs) and BC risk (30).

Given the necessity of MR analysis to validate the three core assumptions (31), we established stringent criteria for selecting the instrumental variables (IVs) to ensure the robustness and reliability of the results (32). First, we set a significance threshold of P<5e−08 to choose SNPs as IVs for each exposure. Second, we addressed linkage disequilibrium (LD) between SNPs by excluding those with strong LD (r2 = 0.001, κb=10,000 kb). The PhenoScanner database was used to mitigate the effects of confounding factors. Finally, we computed the F-statistic for all selected SNPs to evaluate weak instrument bias, excluding SNPs with an F-statistic <10, to ensure that all remaining SNPs were strongly associated with exposure (31).

Causal impacts were estimated using the inverse variation-weighted (IVW) MR as the primary analysis. In instances where exposure exhibited significant effects in the main IVW MR analysis, we applied various robust MR methods (such as the weighted median and MR-Egger) in the sensitivity analyses to address potential violations of the IV assumption (33). We systematically employed leave-one-out modeling to evaluate the potential influence of outliers and pleiotropic SNPs (34). We excluded one SNP at a time to determine whether individual SNPs affected the primary causal relationship. Considering the potential for genetic pleiotropy, interactions, and confounding effects among the different exposure factors, we conducted multivariable MR (MVMR) analyses (35) to assess the direct effects of various exposure factors on BC. To further investigate reverse causation, we performed a reverse MR analysis using BC-related SNPs as IVs (treating BC as the exposure and various risk factors as outcomes).

## 3 Results

### 3.1 Literature retrieval and selection

**Figure 1:**
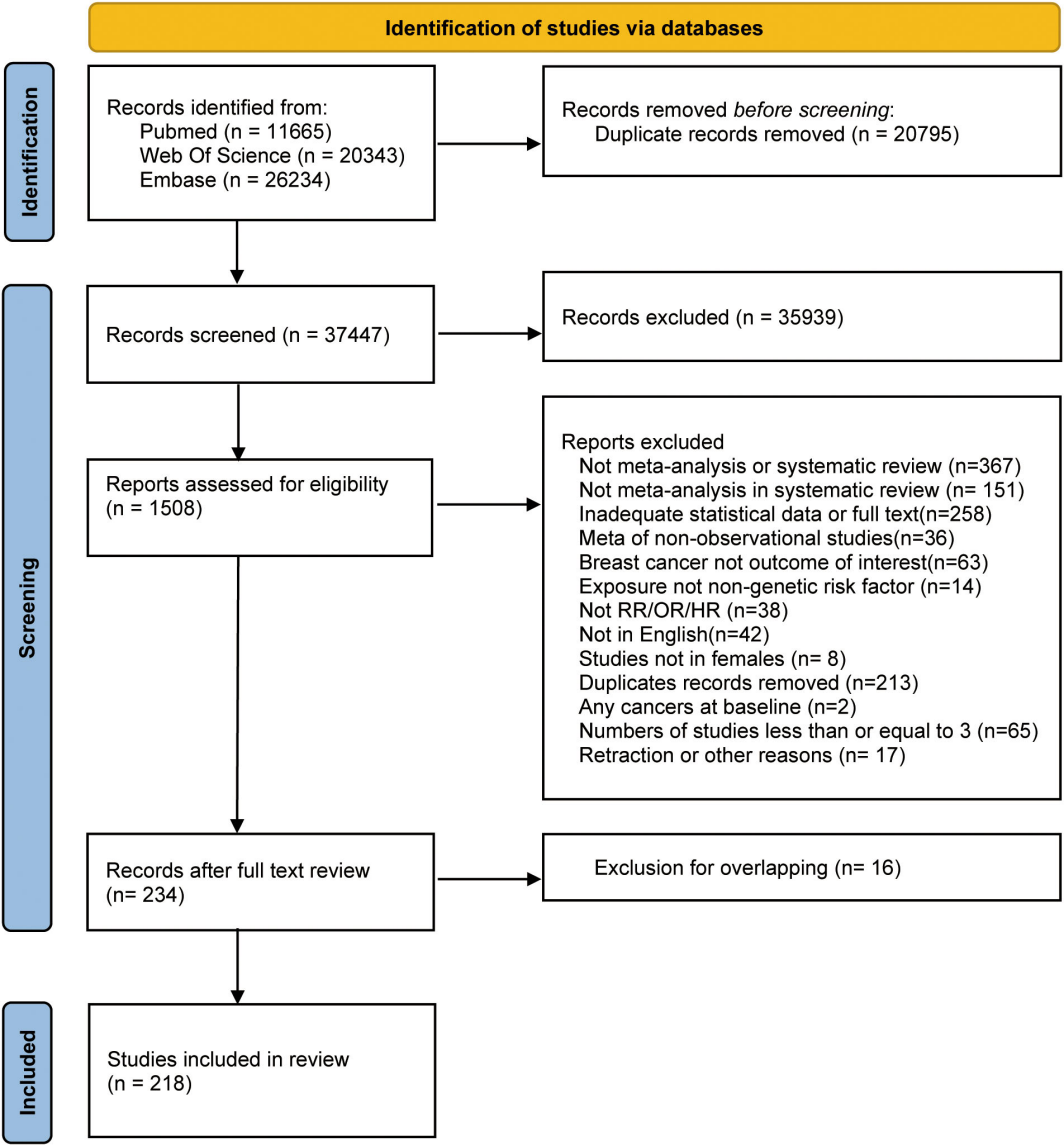
Flow diagram of selection of meta-analyses regarding risk factors for breast cancer.

**Figure 1** depicts the literature retrieval and selection process. Up to July 2023, we retrieved 58,242 articles. After screening and following the exclusion of duplicate meta-analyses based on assessed exposure and outcome (36–51) (**Supplementary Table 7**), 218 articles, including 427 meta-analyses remained (52–269).

### 3.2 Characteristics of meta-analyses

Among the 427 included meta-analyses, the estimated values from studies ranged from 2 to 69 (median, 10). The median number of cases and individuals for each meta-analysis were 14,055 and 3,328,403, respectively. The minimum and maximum numbers of cases in meta-analyses were 138 and 591,297, respectively. The smallest total population was 1,083, whereas the largest was 18,281,388. Furthermore, 403 of the 427 meta-analyses included >1,000 patients with BC.

**Figure 2:**
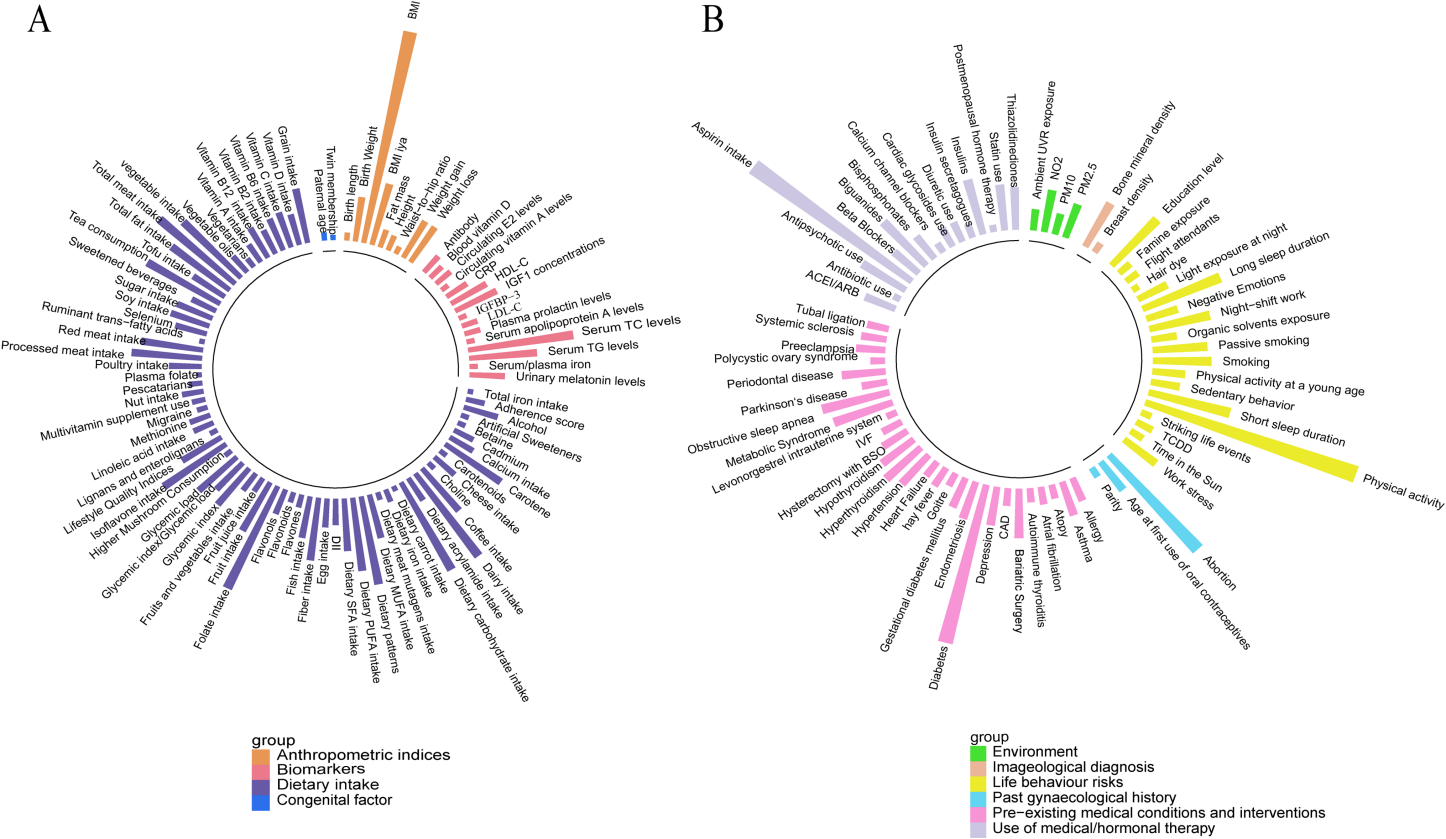
Overall presentation of associations with the risk of breast cancer (cohort studies only) **(A)** Anthropometric indices, biomarkers, congenital factors, and dietary intake. **(B)** Environment, imageological diagnosis, lifestyle behavior, gynecological history, pre-existing medical conditions and interventions, and medical/hormonal therapy use.

From the pool of 427 meta-analyses, 281 consisted exclusively of cohort studies, including at least two that evaluated 202 risk factors and categorized into 10 major groups. (**Figure 2** and **Supplementary Table 2**).

### 3.3 Quality assessment

We evaluated the methodological quality of 218 studies, which included meta-analyses of 427 observational studies, using the AMSTAR-2 tool (**Supplementary Table 4**). Overall, most meta-analyses exhibited low to very low quality owing to various factors such as study design limitations and reduced methodological rigor (such as potential publication bias, indirectness, and inconsistency). Specifically, only a small proportion was rated as having “high” or “moderate” quality.

### 3.4 Main analysis

#### 3.4.1 Summary effect size

When using P<.05 as the threshold for statistical significance, among the 281 studies solely comprising cohort studies, 150 (53%) and 115 (41%) meta-analyses presented significant summary fixed- and random-effects estimates, respectively (**Supplementary Table 2**). Applying a threshold of P<.001, 78 (28%) and 40 (14%) studies produced significant findings using summary fixed- and random-effects models, respectively. In case of P<10^-6^, 49 (17%) and 21 (7%) studies provided significant summary fixed- and random-effects estimates, respectively. In meta-analyses where the random-effects estimate had P<10^-6^, 13 reported different risk factors associated with an increased BC incidence. These thresholds were selected in alignment with the evidence grading system used in this study, which allow for a clearer distinction of the strength and reliability of the findings at each level of evidence.

#### 3.4.2 Heterogeneity between studies

We reanalyzed 281 meta-analyses using random or fixed-effects models and found that 91 (32%) exhibited significant heterogeneity (I² = 50–75%). Notably, 33 (12%) of the meta-analyses showed substantial heterogeneity (I² > 75%) (**Supplementary Table 3**). The heterogeneity observed in most outcomes can be attributed to several factors, including study design, sample size, study quality, environmental influences, and population characteristics. When calculating the 95% PIs, the null hypothesis was excluded in 22 studies, including alcohol, smoking, glycemic index, glycemic index/glycemic load, coffee intake, breast density, IGF-1 concentrations, serum triglyceride (TG) levels and the like.

#### 3.4.3 Grading the evidence

Evidence from 46 meta-analyses suggested the presence of small-study effects. Moreover, evidence of excessive significant bias (P<.10) was observed in 65 meta-analyses with different exposures, including BMI, total meat intake, vegetable intake, metabolic syndrome, dietary calcium intake, and PA. For further details, please refer to **Supplementary Table 3**.

#### 3.4.4 Grading the evidence

We classified the strength of evidence for each risk factor associated with BC (**Table 1**). Moreover, **Supplementary Table 5** presents information on elaborate explanations of the assessment criteria (specifically for cohort studies), whereas **Supplementary Table 6** details the outcomes for all studies.

**Figure 3:**
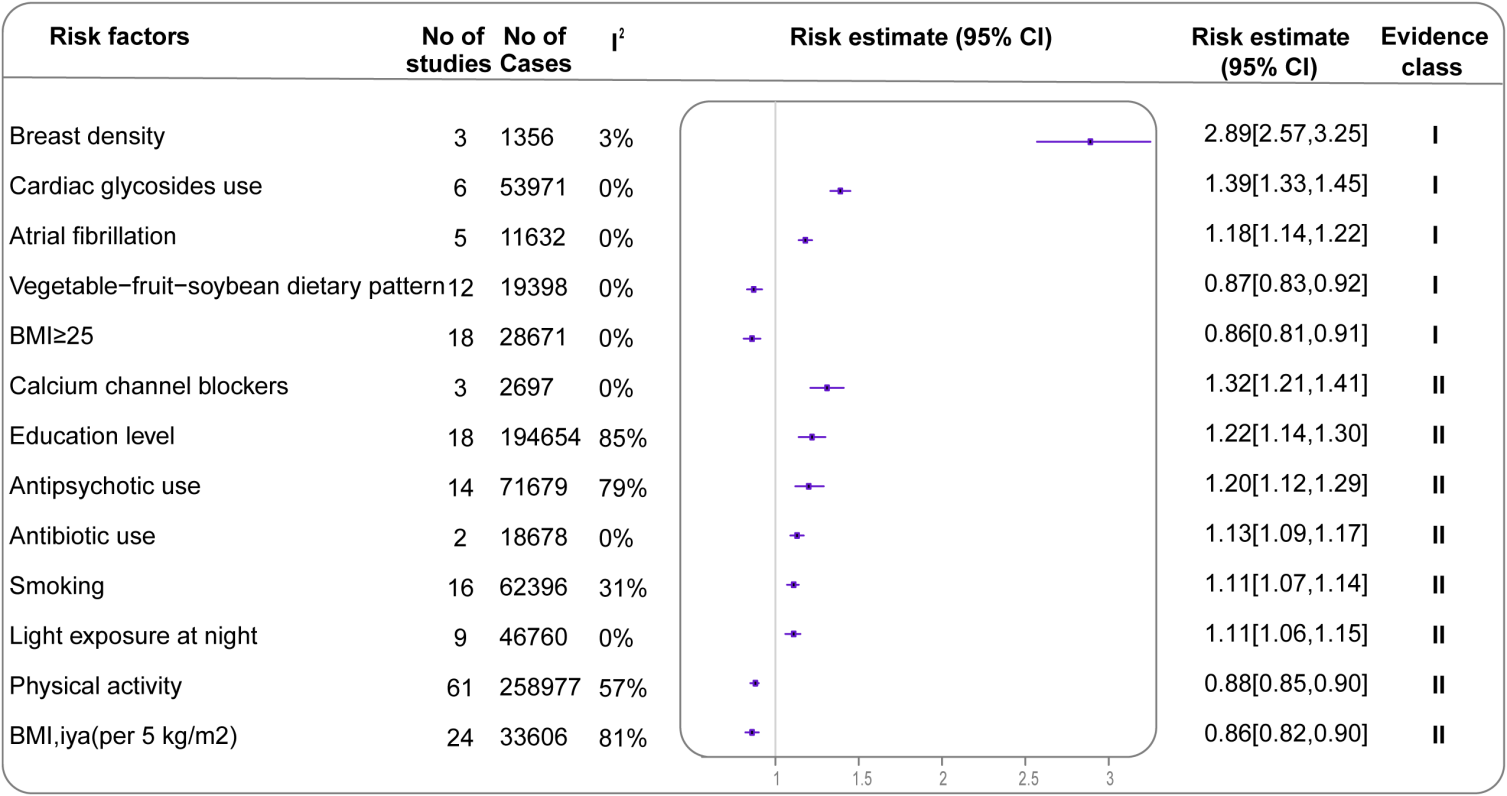
Forest plot of effect estimates and 95% CIs for all exposures associated with BC in the main analysis (summary random effects for cohort studies only) Evidence is graded as strong or highly suggestive (n=13). CI, confidence interval; BC, breast cancer.

Among the 281 meta-analyses included in the primary analysis, only five (1.8%) met the criteria for strong evidence. Including breast density (2.89; 95% CI: 2.57-3.25), cardiac glycosides use (1.39; 95% CI: 1.33-1.45), AF history (1.18; 95% CI: 1.14-1.22), adherence to a vegetable-fruit-soybean dietary pattern (0.87; 95% CI: 0.83-0.92) and Postmenopausal women with a BMI ≥25 (0.86high vs. low; 95% CI: 0.81-0.91) (**Table 1**, **Supplementary Table 5**). Eight analyses (2.8%) provided highly suggestive evidence (**Table 1**, **Figure 3**). Twenty-three analyses (8.2%) provided suggestive evidence, 55 (19.6%) provided weak evidence, and the remaining showed no significant association.

### 3.5 Sensitivity analyses

When cohort and case-control studies were included (**Supplementary Table 6**), additional four exposure factors associated with increased BC incidence provided strong evidence: autoimmune thyroiditis (2.71; 95% CI: 2.13-3.43), weight gain (1.55; 95% CI: 1.40-1.71), oral progestogen (1.28; 95% CI: 1.19-1.39), and light exposure at night (1.13; 95% CI: 1.09-1.16). Strong evidence for two exposures reducing BC incidence was also found: number of births (0.79; 95% CI: 0.75-0.83) and sex hormone-binding globulin (0.65; 95% CI: 0.58-0.73); both associations were only included in case-control studies and were not evaluated in the main analysis. Conversely, when case-control studies were included, the strong correlations between breast density, BMI ≥25 (high vs. low, postmenopausal), and BC risk were downgraded to highly suggestive evidence (2.89; 95% CI: 2.57-3.25) and not significant (0.86; 95% CI: 0.81-0.91), whereas the remaining three strong associations remained strong.

### 3.6 MR

**Figure 4:**
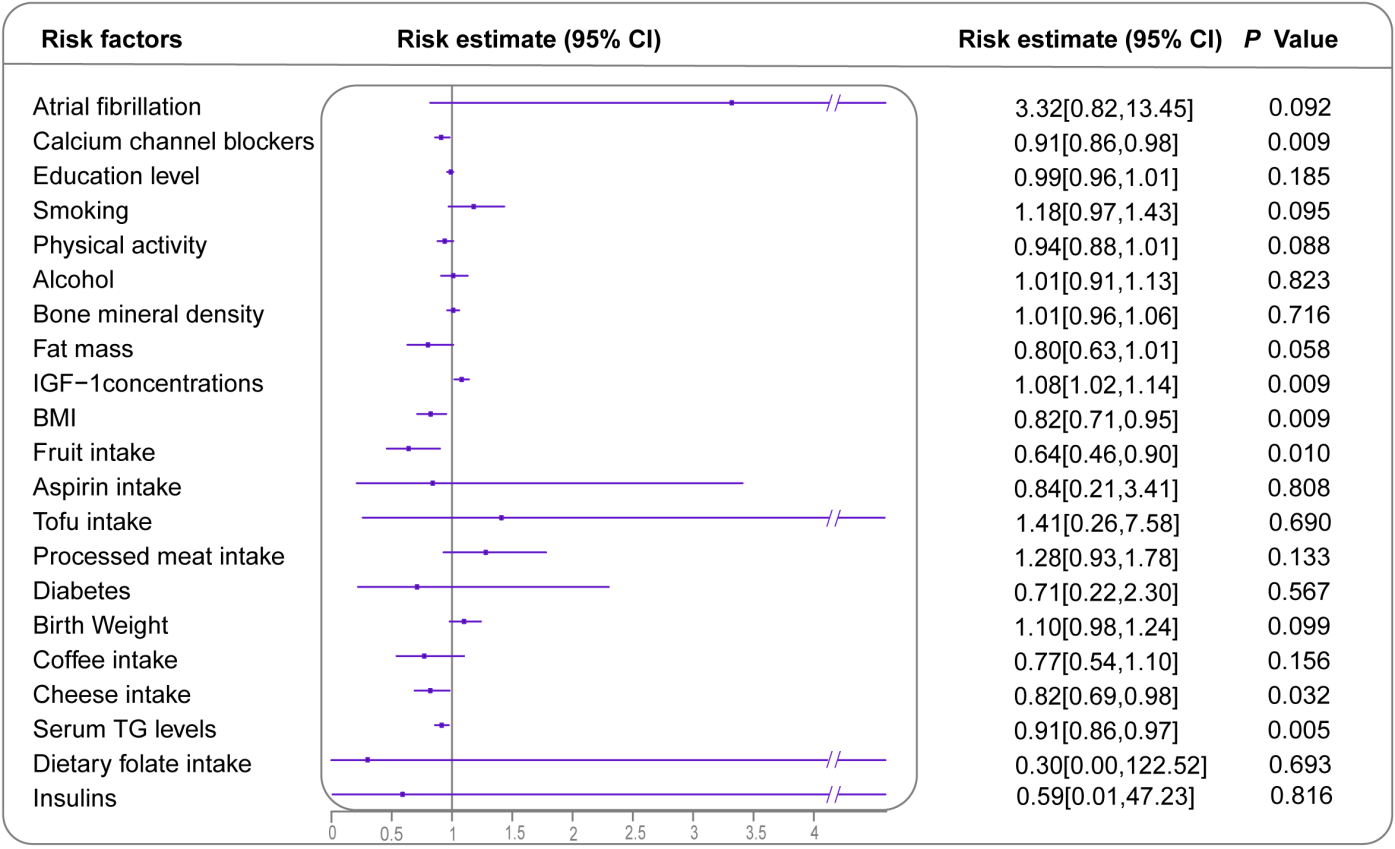
Forest plot demonstrating inverse variance weighted MR results for all identified risk or protective factors for BC with available GWAS. Effect sizes are presented as ORs with 95% CIs. However, the OR for goiter is 28.76 [0.00, 509785.17], which is significantly higher than other values, and is, therefore, not displayed. MR, Mendelian randomization; BC, breast cancer; GWAS, genome-wide association studies; OR, odds ratio; CI, confidence interval.

In the MR analysis, 22 risk factors had available genetic instruments (**Supplementary Table 8**). The genetically predicted IGF-1 concentrations demonstrated a correlation with BC (1.08; 95% CI: 1.02-1.14) (**Figure 4**, **Supplementary Table 9**). Additionally, higher fruit intake (0.64; 95% CI: 0.46-0.90), cheese intake (0.82; 95% CI: 0.69-0.98), higher serum TG levels (0.91; 95% CI: 0.86-0.97) and higher BMI category (0.82; 95% CI: 0.71-0.95) were found to have protective effects against BC. The sensitivity analysis yielded directions consistent with the main analysis, supporting potential causal effects (**Supplementary Figures 1**, **2**, **Supplementary Table 10**). Reverse MR-IVW analysis (**Supplementary Figure 3**) indicated a potential bidirectional relationship between calcium channel blockers (CCBs) and BC. No other exposures demonstrated a similar reverse causal association with BC incidence.

In the MVMR analysis, genetic predictions of CCBs and IGF-1 concentrations showed independent associations with BC after adjusting for BMI and serum TG levels. Similarly, the genetic predictions of fruit and cheese intake were independently linked to a decreased risk of BC (**Supplementary Table 11**).

## 4 Discussion

This umbrella review analyzed data from 427 meta-analyses, with 281 including at least two cohort studies. In the primary analysis, only five meta-analyses provided strong evidence regarding BC, showing significant strength and no bias. Increased breast density, AF history, and cardiac glycoside use were linked to an elevated BC risk. Conversely, adherence to a vegetable-fruit-soybean dietary pattern was associated with a decreased BC incidence. Furthermore, BMI inversely correlated with BC risk among postmenopausal women with a BMI ≥25. Using MR analysis, we identified causal effects between six risk factors and BC: BMI, CCBs, fruit intake, cheese intake, IGF-1 levels, and serum TG levels. CCBs exhibited bidirectional effects on BC.

A meta-analysis examining the relationship between AF history and cardiac glycoside use in BC incidence has garnered substantial evidence. Prior investigations into whether AF increases BC incidence have produced conflicting findings, with this link absent in the WCRF CUP report (270). Hence, our evidence grading system offers valuable supplementary insights. Some studies have proposed that the increased BC risk might stem from shared risk factors such as obesity, diabetes, and unhealthy lifestyles. Nonetheless, these assertions require further validation using clinical data. Another suggested mechanism linking AF to BC involves a systemic inflammatory response (271, 272); however, large-scale epidemiological studies have not confirmed this link. Additionally, cardiac glycosides, particularly digoxin, emerged as potent BC risk factors in our study, possibly because of their estrogenic properties and binding to estrogen receptors (273). In a sizable prospective study involving postmenopausal women (274), adjustment for multiple variables revealed a heightened risk of incident BC associated with AF in women; however, further adjustment for cardiac glycosides mitigated this risk, indicating their potential intermediary role.

Our meta-analysis revealed strong evidence supporting an inverse relationship between BMI and BC incidence, irrespective of menopausal status. Our results are consistent with those of the 2018 WCRF CUP report. Several mechanisms may explain the inverse correlation between BMI and BC risk. First, studies have indicated a negative correlation between breast density and BMI (275, 276), with women who are obese or overweight exhibiting lower breast density and, thus, a reduced BC risk. Second, Among obesity-related protein biomarkers, lower adiponectin levels and higher leptin and IGF-1 levels were associated with an increased BC risk. Adiponectin levels were negatively correlated with BMI and leptin concentration in women (277). Reduced adiponectin levels may enhance insulin signaling (278), which is associated with tumor growth. Another adipokine, leptin, is a key molecular mediator of the relationship between obesity and BC and is overexpressed in individuals who are obese or overweight. Finally, IGF-1, which is mediated by the IGF-1 receptor, is implicated in BC development and progression by regulating proliferation and survival genes via multiple signaling pathways (279). Our comprehensive review, supported by the MR analysis, confirmed the causal relationship between increased IGF-1 concentrations and elevated BC risk, further strengthening our findings.

Substantial suggestive evidence indicates an association between CCB use and an increased BC risk, as confirmed through the MR analysis. Prior studies, such as that by Thakur et al. (174), have suggested an elevated risk of BC with CCB use, whereas Wright et al. (280) indicated no significant association. These differing conclusions likely stem from the high heterogeneity in study design, population, and follow-up duration (281). However, the mechanisms underlying the effect of CCBs on BC risk remain unclear (282). *In vitro* CCB treatment has been shown to upregulate pathways related to breast cell proliferation and migration (283), whereas calcium-dependent processes exhibit tumor-suppressive effects in BC (284). Moreover, the MR reverse causation analysis suggested that BC influences the use of CCBs. This finding reveals a complex bidirectional relationship, highlighting the need for additional research on its mechanisms and clinical implications. Given the uncertainty surrounding the underlying mechanisms, this area presents a novel and important avenue for future studies, particularly in investigating causality pathways and their potential roles in BC risk.

Our study has several strengths. First, we prioritized prospective cohort studies as the main analysis to avoid the influence of epidemiological bias as much as possible. At the same time, in order to make the results comprehensive, all observational studies including case-control studies were further included in the sensitivity analysis, and the differences were discussed. Overlapping articles were assessed using CCA, and the highest quality or most recent reviews of overlapping articles were included to avoid duplicate inclusion. We employed comprehensive and robust methodologies, ensuring the rigor and reliability of our findings. Moreover, the large sample size further enhanced the credibility and precision of our results. Finally, given the inherent limitations of inferring causation from observational studies, this pioneering effort amalgamated umbrella reviews with bidirectional two-sample MR studies in the BC risk domain, providing novel insights into the potential and reverse causal relationships among risk exposures.

However, this study has some limitations. First, it relies on literature retrieval by the original authors and findings from past meta-analyses, potentially leading to some studies being overlooked. Second, we found significant heterogeneity across studies, likely attributable to differences in study design, sample characteristics, and measurement methods. Although random effects models and sensitivity analyses were employed to account for heterogeneity, its impact could not be completely eliminated. Furthermore, the AMSTAR-2 assessment indicated that most of the included studies were of low quality, suggesting a potential risk of bias. Despite conducting bias tests, the exact sources of bias could not be definitively identified. Future research should implement more consistent study designs and enhance methodological quality to minimize both heterogeneity and bias. Third, studies that separately reported the results for pre- and postmenopausal women were limited. Consequently, stratified analyses based on menopausal status were not conducted in this study, potentially overlooking the differential effects of certain exposures owing to differences in menopausal status. Fourth, the umbrella review identified numerous significant exposures. However, the MR analysis has limitations owing to genetic instrument constraints and sample size issues, resulting in fewer established causal effects. This limitation does not imply that the exposures not identified in this analysis lack causal effects, nor does it confirm the absolute accuracy of the conclusions drawn from the MR analysis.

This umbrella review synthesized meta-analyses focusing on the risk factors associated with BC. The MR analysis elucidated the causal relationships between specific risk factors and BC incidence. Identifying these factors facilitates the development of targeted preventive strategies for high-risk populations and investigations into their underlying mechanisms.

## Data Availability

The original contributions presented in the study are included in the article/**Supplementary Material**. Further inquiries can be directed to the corresponding authors.
