# Supplementary material for: Risk factors for breast cancer: an umbrella review of observational cohort studies and causal relationship analysis
Source: Front Oncol. 2025 May 2;15:1541233. doi: 10.3389/fonc.2025.1541233 (PMC12081379; doi:10.3389/fonc.2025.1541233)
Supplement: Supplementary file 1 [file DataSheet1.docx]

**Supplementary Material**

**Supplemental Fig 1.** MR leave−one−out sensitivity analysis for (A) Calcium channel blockers;(B) IGF-1 concentrations(C) BMI (D) Serum TG levels (E) Fruit intake (F) Cheese intake on the breast cancer using MR.

**Supplemental Fig 2.** Scatter plots of effects of (A) Calcium channel blockers;(B) IGF-1 concentrations(C) BMI (D) Serum TG levels (E) Fruit intake (F) Cheese intake on the breast cancer using MR.

**Supplemental Fig 3.** Forest plot demonstrating inverse variance weighted Mendelian randomization results for breast cancer on all identified known risk or protective factors with available GWAS, to determine effect sizes by OR and 95% CI.

**Supplemental Table 1:** Search strategies in the umbrella review.

**Supplemental Table 2:** PRISMA Checklist for Meta-Analysis Reporting.

**Supplemental Table 3:** Description of 281 meta-analyses investigating risk factors associated with breast cancer incidence - only cohort studies included.

**Supplemental Table 4:** Evaluation of heterogeneity, small study effects and excess significance bias in the 281 meta-analyses investigating risk factors associated with breast cancer incidence - only cohort studies included.

**Supplemental Table 5:** A Measurement Tool to Assess Systematic Reviews (AMSTAR-2) Summary quality assessment for all included systematic review.

**Supplemental Table 6:** Details of evidence grading for meta-analyses of risk factors for breast cancer incidence— only cohort studies included.

**Supplemental Table 7：**Details of evidence grading for meta-analyses of risk factors for breast cancer incidence— all study types included.

**Supplemental Table 8.** Evaluation of overlapping associations between included articles in the umbrella review according to the same exposure and outcome.

**Supplemental Table 9.** Mendelian randomization (MR) analysis; exposure-outcome included in the main analysis, cohorts only.

**Supplemental Table 10.** Two-sample inverse variance weighted mendelian randomization full results of the analyses of risk factors on breast cancer.

**Supplemental Table 11.** Results from Mendelian randomization sensitivity analyses of risk factors on breast cancer.

**Supplemental Table 12.** Results from multivariable (MV) Mendelian randomization (MR) sensitivity analyses of risk factors on breast cancer.

**Supplemental Fig 1.** MR leave−one−out sensitivity analysis for (A) Calcium channel blockers;(B) IGF-1 concentrations(C) BMI (D) Serum TG levels (E) Fruit intake (F) Cheese intake on the breast cancer using MR.

**
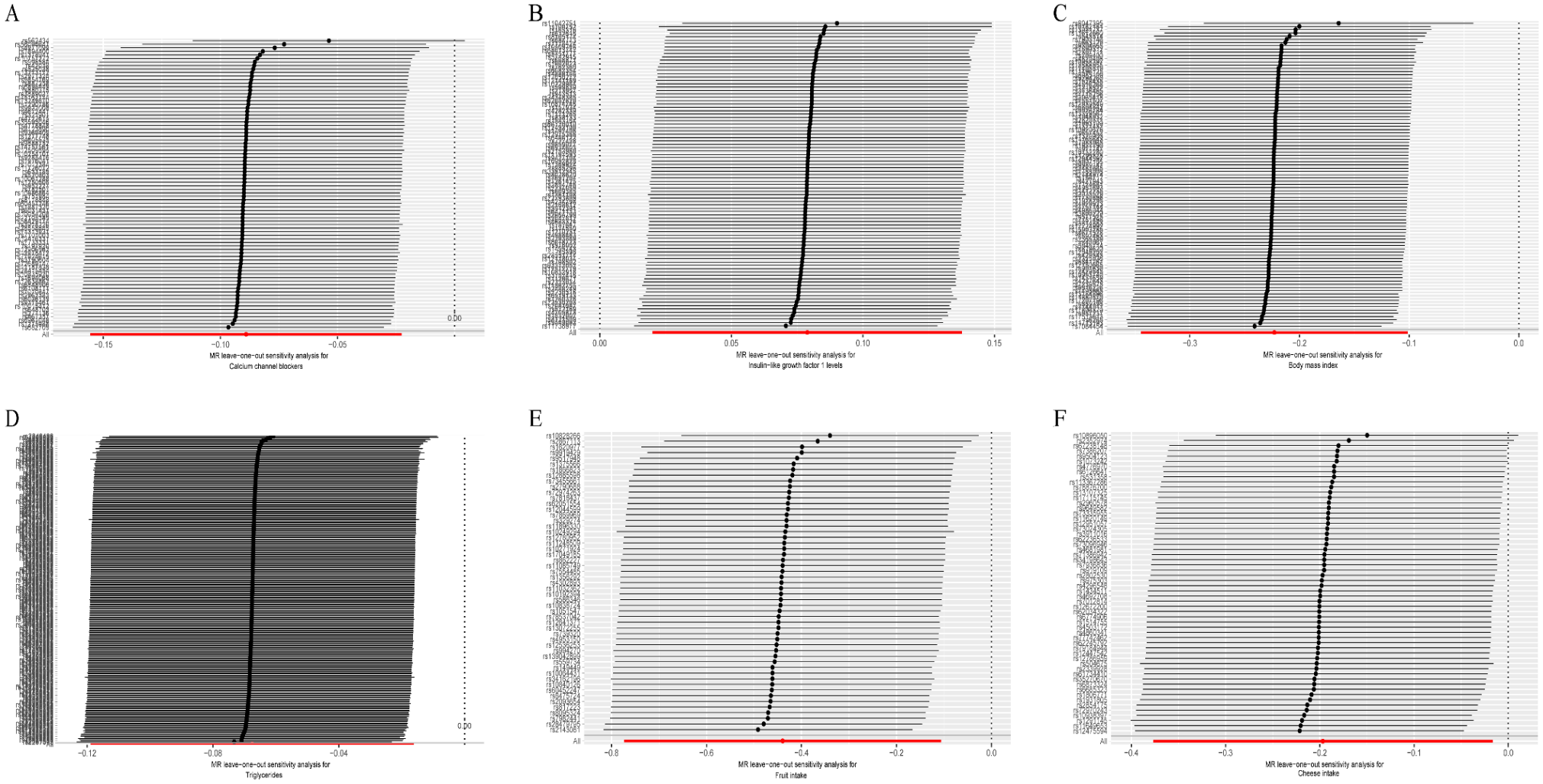
**

**Supplemental Fig 2.** Scatter plots of effects of (A) Calcium channel blockers;(B) IGF-1 concentrations(C) BMI (D) Serum TG levels (E) Fruit intake (F) Cheese intake on the breast cancer using MR.

**
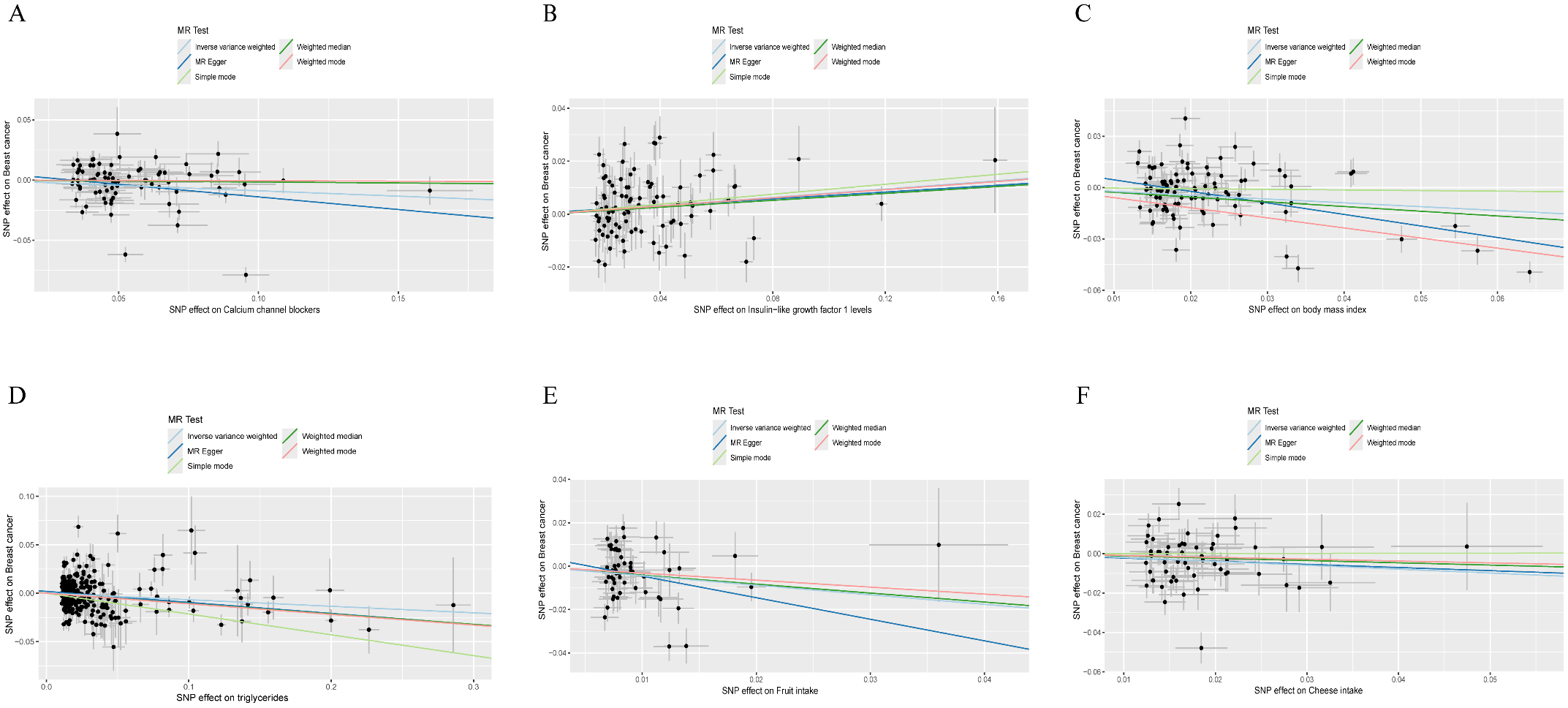
**

**Supplemental Fig 3.** Forest plot demonstrating inverse variance weighted Mendelian randomization results for breast cancer on all identified known risk or protective factors with available GWAS, to determine effect sizes by OR and 95% CI.**
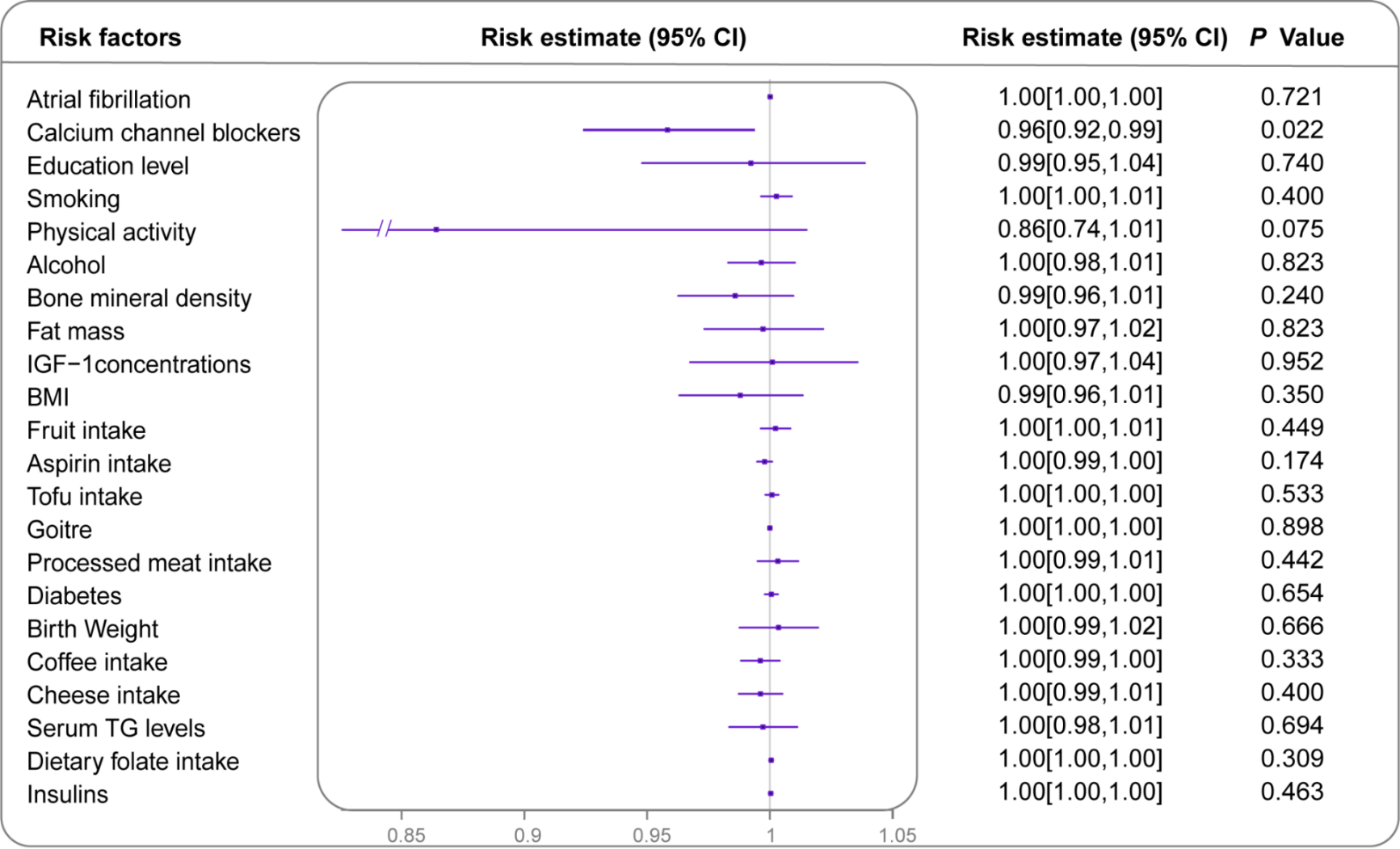
**

**Supplemental Table 1.** Search strategies in the umbrella review

| **Database** | **Literature search strategy** |
| --- | --- |
| PubMed | #1: Breast neoplasm OR breast tumor OR breast cancer OR breast malignant neoplasm OR breast malignant tumor OR breast carcinoma |
|  | #2: meta analysis OR systematic review |
|  | (((#1 AND #2)  Search equation: (((((((((((((((((((("Breast Neoplasms"[Mesh]) OR (Breast Neoplasm[Title/Abstract])) OR (Breast Tumors[Title/Abstract])) OR (Breast Tumor[Title/Abstract])) OR (Breast Cancer[Title/Abstract])) OR (Mammary Cancer[Title/Abstract])) OR (Mammary Cancers[Title/Abstract])) OR (Malignant Neoplasm of Breast[Title/Abstract])) OR (Breast Malignant Neoplasm[Title/Abstract])) OR (Breast Malignant Neoplasms[Title/Abstract])) OR (Malignant Tumor of Breast[Title/Abstract])) OR (Breast Malignant Tumor[Title/Abstract])) OR (Breast Malignant Tumors[Title/Abstract])) OR (Cancer of Breast[Title/Abstract])) OR (Cancer of the Breast[Title/Abstract])) OR (Human Mammary Carcinomas[Title/Abstract])) OR (Human Mammary Carcinoma[Title/Abstract])) OR (Human Mammary Neoplasm[Title/Abstract])) OR (Human Mammary Neoplasms[Title/Abstract])) OR (Breast Carcinoma[Title/Abstract])) OR (Breast Carcinomas[Title/Abstract]) |
| Embase | #1: (breast AND neoplasm) OR (breast AND tumor) OR (breast AND malignant AND neoplasm) OR (breast AND malignant AND tumor) OR (breast AND cancer) OR (breast AND carcinoma) |
|  | #2: (meta AND analysis) OR (systematic AND revuew) |
|  | #3: #1 AND #2 |
|  | Search equation: ((meta analysis) OR (systematic review)) AND ((Breast neoplasm) OR (breast tumor) OR (breast cancer) OR (breast malignant neoplasm) OR (breast malignant tumor) OR (breast carcinoma) |
| Web of Science | #1: TS= (Breast neoplasm OR breast tumor OR breast cancer OR breast malignant neoplasm OR breast malignant tumor OR breast carcinoma) |
|  | #2: TS= (meta analysis OR systematic review) |
|  | #3: #1 AND #2 |
|  | Search equation: (TS= ( Breast neoplasm OR breast tumor OR breast cancer OR breast malignant neoplasm OR breast malignant tumor OR breast carcinoma)) AND (TS=(meta analysis OR systematic review)) |

**Supplemental Table 2.** **PRISMA Checklist for Meta-Analysis Reporting.**

| **Section and Topic** | **Item #** | **Checklist item** | **Location where item is reported** |
| --- | --- | --- | --- |
| **TITLE** | | |  |
| Title | 1 | Identify the report as a systematic review. | 1 |
| **ABSTRACT** | | |  |
| Abstract | 2 | See the PRISMA 2020 for Abstracts checklist. | 1,2 |
| **INTRODUCTION** | | |  |
| Rationale | 3 | Describe the rationale for the review in the context of existing knowledge. | 2 |
| Objectives | 4 | Provide an explicit statement of the objective(s) or question(s) the review addresses. | 2 |
| **METHODS** | | |  |
| Eligibility criteria | 5 | Specify the inclusion and exclusion criteria for the review and how studies were grouped for the syntheses. | 3 |
| Information sources | 6 | Specify all databases, registers, websites, organisations, reference lists and other sources searched or consulted to identify studies. Specify the date when each source was last searched or consulted. | 2 |
| Search strategy | 7 | Present the full search strategies for all databases, registers and websites, including any filters and limits used. | 2, Supplemental Table 1 |
| Selection process | 8 | Specify the methods used to decide whether a study met the inclusion criteria of the review, including how many reviewers screened each record and each report retrieved, whether they worked independently, and if applicable, details of automation tools used in the process. | 3 |
| Data collection process | 9 | Specify the methods used to collect data from reports, including how many reviewers collected data from each report, whether they worked independently, any processes for obtaining or confirming data from study investigators, and if applicable, details of automation tools used in the process. | 3 |
| Data items | 10 | List and define all other variables for which data were sought (e.g. participant and intervention characteristics, funding sources). Describe any assumptions made about any missing or unclear information. | 3 |
| Study risk of bias assessment | 11 | Specify the methods used to assess risk of bias in the included studies, including details of the tool(s) used, how many reviewers assessed each study and whether they worked independently, and if applicable, details of automation tools used in the process. | 3,4 |
| Effect measures | 12 | Specify for each outcome the effect measure(s) (e.g. risk ratio, mean difference) used in the synthesis or presentation of results. | 4 |
| Synthesis methods | 13a | Describe the processes used to decide which studies were eligible for each synthesis (e.g. tabulating the study intervention characteristics and comparing against the planned groups for each synthesis (item #5)). | 4 |
|  | 13b | Describe any methods required to prepare the data for presentation or synthesis, such as handling of missing summary statistics, or data conversions. | 4 |
|  | 13c | Describe any methods used to tabulate or visually display results of individual studies and syntheses. | 4 |
|  | 13d | Describe any methods used to synthesize results and provide a rationale for the choice(s). If meta-analysis was performed, describe the model(s), method(s) to identify the presence and extent of statistical heterogeneity, and software package(s) used. | 4 |
|  | 13e | Describe any methods used to explore possible causes of heterogeneity among study results (e.g. subgroup analysis, meta-regression). | 4 |
|  | 13f | Describe any sensitivity analyses conducted to assess robustness of the synthesized results. | 4 |
| Reporting bias assessment | 14 | Describe any methods used to assess risk of bias due to missing results in a synthesis (arising from reporting biases). | 4 |
| Certainty assessment | 15 | Describe any methods used to assess certainty (or confidence) in the body of evidence for an outcome. | 4 |
| **RESULTS** | | |  |
| Study selection | 16a | Describe the results of the search and selection process, from the number of records identified in the search to the number of studies included in the review, ideally using a flow diagram. | 5, Figure 1 |
|  | 16b | Cite studies that might appear to meet the inclusion criteria, but which were excluded, and explain why they were excluded. | Supplemental Table 7 |
| Study characteristics | 17 | Cite each included study and present its characteristics. | Supplemental Table 2 |
| Risk of bias in studies | 18 | Present assessments of risk of bias for each included study. | Supplemental Table 3 |
| Results of individual studies | 19 | For all outcomes, present, for each study: (a) summary statistics for each group (where appropriate) and (b) an effect estimate and its precision (e.g. confidence/credible interval), ideally using structured tables or plots. | Figure3, Supplemental Table 3 |
| Results of syntheses | 20a | For each synthesis, briefly summarise the characteristics and risk of bias among contributing studies. | Supplemental Table 3 |
|  | 20b | Present results of all statistical syntheses conducted. If meta-analysis was done, present for each the summary estimate and its precision (e.g. confidence/credible interval) and measures of statistical heterogeneity. If comparing groups, describe the direction of the effect. | Supplemental Table 3 |
|  | 20c | Present results of all investigations of possible causes of heterogeneity among study results. | 6, Supplemental Table 5 |
|  | 20d | Present results of all sensitivity analyses conducted to assess the robustness of the synthesized results. | 6, Supplemental Table 5,6 |
| Reporting biases | 21 | Present assessments of risk of bias due to missing results (arising from reporting biases) for each synthesis assessed. | 6,7, Supplemental Table 3 |
| Certainty of evidence | 22 | Present assessments of certainty (or confidence) in the body of evidence for each outcome assessed. | 6,, Supplemental Table 5,6 |
| **DISCUSSION** | | |  |
| Discussion | 23a | Provide a general interpretation of the results in the context of other evidence. | 7 |
|  | 23b | Discuss any limitations of the evidence included in the review. | 8,9 |
|  | 23c | Discuss any limitations of the review processes used. | 8,9 |
|  | 23d | Discuss implications of the results for practice, policy, and future research. | 9 |
| **OTHER INFORMATION** | | |  |
| Registration and protocol | 24a | Provide registration information for the review, including register name and registration number, or state that the review was not registered. | 2 |
|  | 24b | Indicate where the review protocol can be accessed, or state that a protocol was not prepared. | 2 |
| Support | 25 | Describe sources of financial or non-financial support for the review, and the role of the funders or sponsors in the review. | 9 |
| Competing interests | 26 | Declare any competing interests of review authors. | 9 |

*From:*  Page MJ, McKenzie JE, Bossuyt PM, Boutron I, Hoffmann TC, Mulrow CD, et al. The PRISMA 2020 statement: an updated guideline for reporting systematic reviews. BMJ 2021;372:n71. doi: 10.1136/bmj.n71

**Supplemental Table 3:** Description of 281 meta-analyses investigating risk factors associated with breast cancer incidence - only cohort studies included.

| **Author, year** | **Exposure** | **Exposure contrast** | **N^a^** | **Sample size cases/cohort** | **Summary relative risk (95% CI)** | | | **Fixed**  **P-value^b^** | **Random P-value^c^** | **95% Prediction interval^d^** |
| --- | --- | --- | --- | --- | --- | --- | --- | --- | --- | --- |
|  |  |  |  |  | **Fixed Effects** | **Random Effects** | **Largest Study** |  |  |  |
| ***Dietary intake*** | | | | | | | | | | |
| Farvid, M. S.2020 | Fiber intake | highest vs. lowest | 17 | 67160/1962523 | 0.915[0.888, 0.942] | 0.917[0.886, 0.948] | [0.86, 0.94] | 2.80e-09 | 3.51e-07 | [0.87, 0.966] |
| Zhang, L.2019 | Vegetable-fruit-soybean dietary pattern | highest vs. lowest | 12 | 19398/757364 | 0.874[0.83, 0.921] | 0.874[0.83, 0.921] | [0.795, 1.018] | 4.89e-07 | 4.89e-07 | [0.824, 0.928] |
| Shin, S.2023 | Alcohol | highest vs. lowest | 4 | 1251/159858 | 1.842[1.426, 2.379] | 1.842[1.426, 2.379] | [1.16, 2.67] | 2.87e-06 | 2.87e-06 | [1.05, 3.23] |
| Kazemi, A.2021 | Total meat intake | per 100 g/day | 25 | 190014/3979157 | 1.017[1, 1.033] | 1.083[1.033, 1.135] | [0.932, 1.052] | 4.82e-02 | 9.54e-04 | [0.922, 1.272] |
| Li, N.2022 | Fiber intake | highest vs. lowest | 20 | 54845/2207175 | 0.956[0.941, 0.973] | 1.083[0.91, 0.976] | [0.94, 1.02] | 2.27e-07 | 8.41e-04 | [0.87, 1.02] |
| Cai, X.2016 | Selenium | highest vs. lowest | 12 | 7558/142580 | 0.893[0.849,0.939] | 0.893[0.849, 0.939] | [0.519, 0.919] | 1.14e-05 | 1.14e-05 | [0.843, 0.946] |
| Wang, Q.2020 | Tofu intake | highest vs. lowest | 14 | 17075/136740 | 0.815[0.755,0.879] | 0.769[0.674, 0.878] | [0.708, 0.982] | 1.52e-07 | 9.66e-05 | [0.512, 1.157] |
| Malcomson, F. C.2023 | Adherence score | highest vs. lowest | 7 | 72420/1449105 | 0.723[0.679,0.77] | 0.736[0.646, 0.839] | [0.626, 0.869] | 7.09e-24 | 4.33e-06 | [0.506, 1.07] |
| Li, D.Y.2018 | DII | per 1 | 6 | 15007/172704 | 1.195[1.112,1.284] | 1.325[1.043, 1.683] | [1.008, 1.759] | 1.32e-06 | 2.14e-02 | [0.586, 2.996] |
| Chen, J.Y.2016 | Wine Drinking | highest vs. lowest | 9 | 10220/541052 | 1.183[1.09,1.283] | 1.222[1.037, 1.439] | [0.654, 1.492] | 5.80e-05 | 1.66e-02 | [0.779, 1.917] |
| Kazemi, A.2021 | Processed meat intake | per 50g /day | 17 | 92323/2522068 | 1.091[1.028,1.158] | 1.178[1.04, 1.333] | [0.438, 1.219] | 4.21e-03 | 9.77e-03 | [0.788, 1.761] |
| Pan, B.2023 | SSBs | per 250ml/day | 7 | 14886/455697 | 1.022[0.988,1.057] | 1.171[1.004, 1.365] | [0.968, 1.038] | 2.04e-01 | 4.38e-02 | [0.779, 1.76] |
| Cao, Y.2016 | Total fat intake | highest vs. lowest | 20 | 35344/1220608 | 1.055[1.011,1.101] | 1.103[1.021, 1.192] | [0.968, 1.209] | 1.34e-02 | 1.24e-02 | [0.882, 1.381] |
| Anderson,J.J.2018 | Processed meat intake | highest vs. lowest | 8 | 25170/1305016 | 1.074[1.027,1.123] | 1.102[1.006, 1.208] | [0.942, 1.122] | 1.88e-03 | 3.74e-02 | [0.839, 1.448] |
| Rezaianzadeh, A.2018 | Red meat intake | highest vs. lowest | 8 | 20585/937330 | 1.083[1.004,1.168] | 1.0887[1.001, 1.18] | [0.788, 1.199] | 3.91e-02 | 4.68e-02 | [0.954, 1.239] |
| Farvid, M. S.2021 | Red meat intake | highest vs. lowest | 14 | 39205/1171894 | 1.069[1.032,1.107] | 1.085[1.023, 1.15] | [0.982, 1.141] | 2.04e-04 | 6.61e-03 | [0.911, 1.291] |
| Farvid, M. S.2021 | Processed meat intake | highest vs. lowest | 16 | 35157/1045950 | 1.06[1.023,1.098] | 1.06[1.007, 1.116] | [0.958, 1.178] | 1.16e-03 | 2.55e-02 | [0.924, 1.216] |
| Turati, F.2015 | Glycemic index | highest vs. lowest | 13 | 50464/1255092 | 1.052[1.015,1.09] | 1.052[1.015, 1.09] | [0.988, 1.119] | 5.64e-03 | 5.64e-03 | [1.01, 1.095] |
| Long, T.2022 | Glycemic index/Glycemic load | highest vs. lowest | 14 | 66280/1162453 | 1.049[1.013,1.086] | 1.049[1.013, 1.086] | [0.988, 1.119] | 7.10e-03 | 7.10e-03 | [1.009, 1.09] |
| Schlesinger, S.2017 | Glycemic index | per 10 units/day | 10 | 36826/1110003 | 1.037[1.009,1.065] | 1.037[1.003, 1.072] | [1.008, 1.118] | 9.36e-03 | 3.37e-02 | [0.965, 1.115] |
| Kazemi, A.2021 | Vegetable intake | per 100g /day | 14 | 76875/1550521 | 0.994[0.984,1.005] | 0.974[0.952, 0.996] | [0.91, 0.99] | 3.17e-01 | 1.87e-02 | [0.909, 1.043] |
| Kazemi, A.2021 | Fruit intake | per 100 g/day | 15 | 80434/1558220 | 0.978[0.969,0.987] | 0.969[0.95, 0.988] | [0.918, 0.968] | 9.10e-07 | 1.74e-03 | [0.911, 1.03] |
| Kazemi, A.2021 | Soy intake | per 30g /day | 7 | 4122/15528 | 0.967[0.94,0.994] | 0.967[0.94, 0.994] | [0.691, 1.171] | 1.63e-02 | 1.63e-02 | [0.932, 1.002] |
| Wei, Y.2020 | Soy isoflavone | per 10 mg/day | 9 | 10229/631498 | 0.967[0.948,0.987] | 0.967[0.945, 0.99] | [0.896, 1.057] | 1.08e-03 | 5.18e-03 | [0.926, 1.01] |
| Aune, D.2012 | Fruits and vegetables intake | per 200g /day | 6 | 6220/226880 | 0.966[0.933,1] | 0.966[0.933, 1] | [0.894, 1.044] | 4.77e-02 | 4.77e-02 | [0.92, 1.014] |
| Li, Y. 2021 | Coffee intake | highest vs. lowest | 15 | 53119/1524226 | 0.958[0.923,0.995] | 0.958[0.923, 0.995] | [0.898, 1.041] | 2.57e-02 | 2.57e-02 | [0.92, 0.999] |
| Song, D.2019 | Vitamin D intake | highest vs. lowest | 12 | 24608/886437 | 0.955[0.915,0.997] | 0.955[0.915, 0.997] | [0.943, 1.142] | 3.41e-02 | 3.41e-02 | [0.91, 1.002] |
| Kazemi, A.2021 | Cheese intake | per 30g /day | 10 | 46744/1419872 | 0.986[0.971,1] | 0.952[0.909, 0.996] | [0.948, 1.018] | 5.25e-02 | 3.30e-02 | [0.839, 1.079] |
| Aune, D.2012 | B-carotene | per 5000ug/day | 10 | 18191/825911 | 0.95[0.914,0.988] | 0.95[0.914, 0.988] | [0.908, 1.018] | 9.55e-03 | 9.55e-03 | [0.908, 0.994] |
| Liu, F.2022 | Flavonols | highest vs. lowest | 6 | 6325/515174 | 0.942[0.897,0.99] | 0.942[0.897, 0.99] | [0.884, 1.034] | 1.79e-02 | 1.79e-02 | [0.879, 1.01] |
| Aune, D.2012 | Fruit intake | per 100 g/day | 10 | 16763/785668 | 0.947[0.908,0.988] | 0.939[0.886, 0.996] | [0.987, 1.188] | 1.14e-02 | 3.67e-02 | [0.81, 1.09] |
| Ghoreishy, S. M.2023 | Dietary calcium intake | per 350 mg/day | 6 | 47823/1575204 | 0.976[0.967,0.986] | 0.937[0.888, 0.988] | [0.97, 0.99] | 1.41e-06 | 1.66e-02 | [0.818, 1.073] |
| Zeng, J.2020 | Dietary folate intake | highest vs. lowest | 23 | 42208/1191707 | 0.95[0.918,0.983] | 0.934[0.88, 0.991] | [0.832, 1.012] | 3.35e-03 | 2.39e-02 | [0.754, 1.156] |
| Brennan, S. F.2010 | Prudent/healthy dietary pattern | highest vs. lowest | 8 | 13885/465891 | 0.926[0.874,0.981] | 0.928[0.871, 0.989] | [0.68, 1.19] | 9.03e-03 | 2.04e-02 | [0.83, 1.037] |
| Aune, D.2012 | Fruit intake | highest vs. lowest | 10 | 16763/785668 | 0.921[0.87,0.975] | 0.92[0.864, 0.979] | [0.943, 1.253] | 4.64e-03 | 8.74e-03 | [0.833, 1.016] |
| Malcomson, F. C.2023 | Adherence score | per 1-point | 5 | 68990/1441887 | 0.91[0.89,0.93] | 0.917[0.863, 0.974] | [0.876, 0.938] | 5.32e-17 | 5.15e-03 | [0.742, 1.134] |
| Hu, F.2012 | A-carotene | highest vs. lowest | 5 | 11447/363730 | 0.913[0.848,0.982] | 0.913[0.848, 0.982] | [0.832, 1.06] | 1.45e-02 | 1.45e-02 | [0.81, 1.028] |
| Zeng, J.2020 | Vitamin B2 | highest vs. lowest | 10 | 20100/622953 | 0.928[0.876,0.982] | 0.899[0.819, 0.986] | [0.882, 1.042] | 1.00e-02 | 2.46e-02 | [0.712, 1.134] |
| Aune, D.2012 | Fruits and vegetables intake | highest vs. lowest | 6 | 5206/188188 | 0.89[0.802,0.989] | 0.89[0.802, 0.989] | [0.76, 1.09] | 3.01e-02 | 3.01e-02 | [0.768, 1.033] |
| Dong, J. Y.2011 | Total dairy food intake | highest vs. lowest | 10 | 19339/662579 | 0.909[0.859,0.962] | 0.876[0.795, 0.965] | [0.882, 1.042] | 9.85e-04 | 7.22e-03 | [0.694, 1.106] |
| Ghoreishy, S. M2023. | Dietary calcium intake | highest vs. lowest | 7 | 47911/1579901 | 0.93[0.878,0.985] | 0.873[0.77, 0.989] | [0.912, 1.071] | 1.34e-02 | 3.34e-02 | [0.63, 1.208] |
| Zheng, J. S.2013 | Dietary PUFA intake | highest vs. lowest | 11 | 12296/480475 | 0.928[0.881,0.977] | 0.861[0.771, 0.961] | [0.8, 1.38] | 4.55e-03 | 7.85e-03 | [0.612, 1.211] |
| Shin, S.2023 | Isoflavone intake | highest vs. lowest | 4 | 2180/172652 | 0.845[0.744,0.96] | 0.849[0.726, 0.992] | [0.59, 1.11] | 9.58e-03 | 3.97e-02 | [0.53, 1.359] |
| Parra-Soto, S.2022 | Vegetarians | yes vs.no | 4 | 471/558626 | 0.851[0.746,0.97] | 0.849[0.722, 0.998] | [0.539, 0.99] | 1.60e-02 | 4.68e-02 | [0.503, 1.431] |
| Ba, D. M.2021 | Higher mushroom consumption | highest vs. lowest | 3 | 11105/100773 | 0.851[0.759,0.954] | 0.83[0.705, 0.977] | [0.77, 1.03] | 5.83e-03 | 2.54e-02 | [0.175, 3.941] |
| Boyd, N. F.2003 | Total fat intake | highest vs. lowest | 14 | 8735/577284 | 0.986[0.958,1.016] | 1.114[0.995, 1.248] | [0.94, 1] | 3.59e-01 | 6.20e-02 | [0.809, 1.534] |
| Shin, S.2023 | Green tea | highest vs. lowest | 3 | 1387/151238 | 1.102[0.931,1.304] | 1.102[0.931, 1.304] | [0.868, 1.609] | 2.57e-01 | 2.57e-01 | [0.37, 3.281] |
| Cao, Y.2016 | Dietary MUFA intake | highest vs. lowest | 17 | 33457/1037980 | 1.06[1.009,1.115] | 1.085[0.974, 1.207] | [0.958, 1.199] | 2.16e-02 | 1.38e-01 | [0.781, 1.506] |
| Cao, Y.2016 | Dietary SFA intake | highest vs. lowest | 20 | 35344/1220608 | 1.062[1.014,1.112] | 1.08[0.99, 1.177] | [1.03, 1.26] | 1.07e-02 | 8.20e-02 | [0.83, 1.404] |
| Mullie, P.2016 | Glycemic load | highest vs. lowest | 12 | 30438/1659097 | 1.047[1.004,1.092] | 1.076[0.995, 1.163] | [0.887, 1.167] | 3.21e-02 | 6.68e-02 | [0.839, 1.379] |
| Li, C.2016 | Dietary cholesterol intake | highest vs. lowest | 6 | 5442/346959 | 1.07[0.994,1.151] | 1.072[0.985, 1.167] | [0.977, 1.157] | 7.11e-02 | 1.09e-01 | [0.926, 1.242] |
| Kolahdouz Mohammadi, R.2017 | Ruminant trans-fatty acids | highest vs. lowest | 2 | 3893/129224 | 1.063[0.951,1.189] | 1.069[0.941, 1.215] | [0.921, 1.171] | 2.84e-01 | 3.07e-01 | NA |
| Chen, H.2021 | DII | highest vs. lowest | 6 | 15772/308000 | 1.047[0.992,1.106] | 1.061[0.982, 1.146] | [0.912, 1.071] | 9.23e-02 | 1.34e-01 | [0.882, 1.275] |
| Chang, V. C.2019 | Dietary iron intake | highest vs. lowest | 5 | 7862/223709 | 1.032[0.955,1.116] | 1.057[0.913, 1.224] | [0.901, 1.151] | 4.24e-01 | 4.59e-01 | [0.67, 1.667] |
| Anderson, J. J.2018 | Red meat intake | highest vs. lowest | 10 | 30952/1455252 | 1.045[1.003,1.087] | 1.057[0.992, 1.127] | [0.93, 1.12] | 3.37e-02 | 8.76e-02 | [0.886, 1.261] |
| Florez-Garcia, V. A.2023 | Cadmium | highest vs. lowest | 8 | 15203/367629 | 1.033[0.975,1.095] | 1.056[0.944, 1.181] | [0.81, 1] | 2.66e-01 | 3.44e-01 | [0.762, 1.463] |
| Farvid, M. S.2021 | Total red and processed meat | highest vs. lowest | 12 | 29810/932398 | 1.036[0.995,1.078] | 1.052[0.994, 1.113] | [0.958, 1.178] | 8.49e-02 | 8.08e-02 | [0.92, 1.203] |
| Cao, Y.2016 | Dietary PUFA intake | highest vs. lowest | 16 | 33209/1012088 | 1.004[0.963,1.047] | 1.047[0.956, 1.147] | [0.909, 1.079] | 8.55e-01 | 3.22e-01 | [0.798, 1.374] |
| Mullie, P.2016 | Glycemic index | highest vs. lowest | 13 | 30438/1657852 | 1.039[1,1.08] | 1.045[0.99, 1.103] | [0.943, 1.113] | 5.23e-02 | 1.13e-01 | [0.907, 1.204] |
| Shin, S.2023 | Unhealthy Dietary pattern | highest vs. lowest | 3 | 974/107589 | 1.056[0.887,1.258] | 1.043[0.736, 1.477] | [1.028, 1.699] | 5.38e-01 | 8.13e-01 | [0.023, 47.244] |
| Kim, Y.2020 | Dietary PUFA intake | highest vs. lowest | 13 | 12364/474607 | 1.015[1.006,1.025] | 1.043[0.975, 1.116] | [1.081, 1.181] | 1.96e-03 | 2.23e-01 | [0.882, 1.233] |
| Li, N.2022 | Sugar intake | highest vs. lowest | 10 | 17937/613718 | 1.03[0.979,1.083] | 1.036[0.944, 1.137] | [0.851, 1.081] | 2.59e-01 | 4.52e-01 | [0.794, 1.352] |
| Nie, X. C.2014 | Black tea consumption | highest vs. lowest | 7 | 3946/1135827 | 0.999[0.92,1.085] | 1.035[0.907, 1.181] | [0.667, 0.988] | 9.84e-01 | 6.10e-01 | [0.715, 1.499] |
| Kazemi, A.2021 | Egg intake | per 50g /day | 11 | 45508/1294387 | 1.021[0.979,1.066] | 1.034[0.956, 1.118] | [0.959, 1.219] | 3.31e-01 | 3.99e-01 | [0.845, 1.266] |
| Nindrea, R. D.2019 | Dietary PUFA intake | highest vs. lowest | 6 | 156039/270838 | 1.043[0.965,1.128] | 1.032[0.933, 1.141] | [0.991, 1.22] | 2.84e-01 | 5.45e-01 | [0.834, 1.276] |
| Pan, B.2023 | ASBs | per 250ml/day | 3 | 13018/287915 | 0.973[0.921,1.028] | 1.031[0.865, 1.23] | [0.908, 1.018] | 3.22e-01 | 7.32e-01 | [0.154, 6.919] |
| Hui, C.2013 | Total flavonoids | highest vs. lowest | 4 | 2168/49957 | 1.026[0.877,1.2] | 1.026[0.877, 1.2] | [0.85, 1.25] | 7.47e-01 | 7.47e-01 | [0.727, 1.448] |
| Long, T.2022 | Glycemic load | highest vs. lowest | 14 | 66280/1162453 | 1.031[0.99,1.074] | 1.024[0.953, 1.1] | [1.001, 1.141] | 1.36e-01 | 5.21e-01 | [0.834, 1.256] |
| Yu, F.2014 | Tea consumption | per 3 cups | 15 | 20500/725390 | 1.018[0.98,1.056] | 1.021[0.976, 1.068] | [0.752, 1.271] | 3.61e-01 | 3.62e-01 | [0.929, 1.122] |
| Wang, Y.2020 | Green tea | highest vs. lowest | 6 | 2470/242418 | 1.014[0.845,1.217] | 1.014[0.845, 1.217] | [0.598, 2.79] | 8.80e-01 | 8.80e-01 | [0.784, 1.312] |
| Van Puyvelde, H.2023 | Choline | highest vs. lowest | 3 | 15342/483933 | 1.019[0.962,1.08] | 1.013[0.918, 1.117] | [0.929, 1.079] | 5.23e-01 | 8.03e-01 | [0.364, 2.814] |
| Shin, S.2023 | Soyfoods intake | highest vs. lowest | 3 | 1104/94779 | 1.009[0.807,1.262] | 1.012[0.719, 1.426] | [0.79, 1.45] | 9.36e-01 | 9.44e-01 | [0.029, 35.678] |
| Ye, X.2023 | Artificial Sweeteners | highest vs. lowest | 3 | 8855/3389003 | 0.989[0.961,1.018] | 1.009[0.918, 1.11] | [0.954, 1.014] | 4.69e-01 | 8.50e-01 | [0.349, 2.922] |
| Zhang, D.2020 | Vitamin C intake | highest vs. lowest | 14 | 31825/194907 | 1.009[0.966,1.054] | 1.009[0.966, 1.054] | [0.918, 1.129] | 6.72e-01 | 6.72e-01 | [0.962, 1.06] |
| Brennan, S. F.2010 | Western/unhealthy dietary pattern | highest vs. lowest | 8 | 13885/465891 | 1.003[0.936,1.074] | 1.005[0.92, 1.096] | [0.709, 1.329] | 9.42e-01 | 9.18e-01 | [0.822, 1.228] |
| Kazemi, A.2021 | Cereals intake | per 20g /day | 13 | 21558/492066 | 1.003[0.996,1.009] | 1.003[0.996, 1.009] | [0.948, 1.079] | 4.52e-01 | 4.52e-01 | [0.995, 1.01] |
| Kazemi, A.2021 | Fruit juice intake | per 50g /day | 6 | 12627/337255 | 0.996[0.989,1.004] | 1[0.988, 1.013] | [0.988, 1.018] | 3.53e-01 | 9.60e-01 | [0.972, 1.03] |
| Chan, A. L.2011 | Multivitamin supplement use | highest vs. lowest | 5 | 56339/342512 | 0.994[0.947,1.043] | 1[0.922, 1.084] | [0.889, 1.059] | 8.02e-01 | 9.96e-01 | [0.782, 1.279] |
| Sun, S.2016 | Choline | highest vs. lowest | 2 | 5022/165247 | 1.047[0.955,1.148] | 0.999[0.804, 1.242] | [0.721, 1.071] | 3.28e-01 | 9.95e-01 | NA |
| Kazemi, A.2021 | Fish intake | per 100g /day | 16 | 42485/6303773 | 0.987[0.938,1.038] | 0.994[0.926, 1.068] | [0.624, 1.262] | 6.04e-01 | 8.79e-01 | [0.856, 1.155] |
| Ren, X.2020 | Folate intake | per 100ug /day | 16 | 33176/1072045 | 0.996[0.991,1.001] | 0.994[0.985, 1.002] | [0.95, 1.03] | 9.20e-02 | 1.61e-01 | [0.969, 1.019] |
| Schlesinger, S.2017 | Sugar intake | per 10g /day | 4 | 12397/378138 | 0.993[0.985,1.001] | 0.994[0.981, 1.006] | [0.98, 1] | 1.00e-01 | 3.26e-01 | [0.952, 1.037] |
| Zeng, J. 2020 | Vitamin B12 | highest vs. lowest | 13 | 25665/749051 | 0.985[0.936,1.038] | 0.993[0.933, 1.056] | [0.891, 1.031] | 5.76e-01 | 8.17e-01 | [0.891, 1.106] |
| Ghoreishy, S. M. | Total calcium intake | per 350 mg/day | 3 | 38782/1186688 | 0.992[0.977,1.007] | 0.992[0.977, 1.007] | [0.978, 1.008] | 2.93e-01 | 2.93e-01 | [0.899, 1.094] |
| Kazemi, A.2021 | Milk intake | per 200g /day | 13 | 51327/1663814 | 0.995[0.981,1.009] | 0.99[0.961, 1.019] | [0.961, 1.081] | 4.76e-01 | 5.01e-01 | [0.915, 1.071] |
| Lei, L.2016 | Flavan-3-ols | highest vs. lowest | 4 | 25420/730471 | 0.986[0.92,1.056] | 0.985[0.908, 1.068] | [0.905, 1.079] | 6.85e-01 | 7.14e-01 | [0.783, 1.239] |
| Li, N.2022 | Dietary carbohydrate intake | highest vs. lowest | 38 | 131058/4610498 | 0.97[0.96,0.98] | 0.983[0.954, 1.014] | [0.955, 1.008] | 1.27e-09 | 2.84e-01 | [0.88, 1.1] |
| Sun, S.2016 | Betaine | highest vs. lowest | 2 | 5022/165247 | 0.983[0.897,1.078 | 0.983[0.897, 1.078] | [0.795, 1.223] | 7.17e-01 | 7.17e-01 | NA |
| Reng, Q.2022 | Dietary meat mutagens intake | highest vs. lowest | 3 | 7340/713871 | 0.98[0.907,1.058] | 0.982[0.898, 1.074] | [0.802, 1.051] | 6.01e-01 | 6.97e-01 | [0.462, 2.091] |
| Zhao, T. T.2019 | Isoflavone intake | highest vs. lowest | 11 | 15838/809232 | 0.993[0.952,1.035] | 0.98[0.917, 1.048] | [0.941, 1.194] | 7.27e-01 | 5.57e-01 | [0.806, 1.192] |
| Aune, D.2012 | Dietary total carotenoids | per 10000 µg/day | 3 | 4290/208276 | 0.997[0.881,1.128] | 0.98[0.786, 1.223] | [0.819, 1.15] | 9.60e-01 | 8.60e-01 | [0.085, 11.332] |
| Van Puyvelde, H.2023 | Betaine | highest vs. lowest | 3 | 18342/483933 | 0.975[0.917,1.037] | 0.975[0.917, 1.037] | [0.892, 1.052] | 4.17e-01 | 4.17e-01 | [0.655, 1.452] |
| Kazemi, A.2021 | Dairy intake | per 200g /day | 10 | 17886/544481 | 0.967[0.958,0.976] | 0.975[0.946, 1.004] | [0.95, 0.97] | 7.20e-13 | 9.53e-02 | [0.905, 1.05] |
| Song, D.2019 | Vitamin D intake | per 400IU/d | 7 | 14900/470280 | 0.974[0.94,1.009] | 0.974[0.94, 1.009] | [0.608, 1.211] | 1.39e-01 | 1.39e-01 | [0.93, 1.02] |
| Fabiani, R.2016 | Apple intake | highest vs. lowest | 3 | 9195/479219 | 0.973[0.935,1.013] | 0.973[0.935, 1.013] | [0.93, 1.01] | 1.84e-01 | 1.84e-01 | [0.75, 1.263] |
| Ghoreishy, S. M.2023 | Total calcium intake | highest vs. lowest | 3 | 38782/1186688 | 0.97[0.909,1.035] | 0.97[0.909, 1.035] | [0.908, 1.039] | 3.58e-01 | 3.58e-01 | [0.636, 1.479] |
| Ren, X.2020 | Folate intake | highest vs. lowest | 19 | 37917/1367403 | 0.971[0.935,1.007] | 0.97[0.913, 1.029] | [0.832, 1.012] | 1.13e-01 | 3.12e-01 | [0.791, 1.188] |
| Touvier, M.2015 | Total cholesterol | per 1 mmol/L | 13 | 21436/992565 | 0.972[0.957,0.987] | 0.97[0.939, 1.002] | [1, 1.08] | 2.79e-04 | 6.22e-02 | [0.878, 1.071] |
| Xiao, Y.2018 | Whole grain intake | highest vs. lowest | 4 | 5734/114433 | 0.963[0.882,1.052] | 0.968[0.821, 1.143] | [0.801, 1.031] | 4.01e-01 | 7.05e-01 | [0.485, 1.933] |
| Kazemi, A.2021 | Poultry intake | per 100g /day | 12 | 32492/1098878 | 0.958[0.92,0.998] | 0.966[0.903, 1.033] | [0.902, 1.172] | 3.88e-02 | 3.16e-01 | [0.835, 1.117] |
| Lafranconi, A.2018 | Coffee intake | highest vs. lowest | 15 | 46798/1403158 | 0.965[0.929,1.001] | 0.965[0.927, 1.003] | [0.898, 1.041] | 5.91e-02 | 7.38e-02 | [0.911, 1.021] |
| Zhou, Y.2016 | Linoleic acid intake | highest vs. lowest | 8 | 9483/356396 | 0.959[0.914,1.064] | 0.962[0.834, 1.11] | [0.585, 1.715] | 7.21e-01 | 5.98e-01 | [0.671, 1.38] |
| Xin, Y.2015 | Total vegetable oils | highest vs. lowest | 5 | 4854/171953 | 0.973[0.866,1.094] | 0.961[0.75, 1.231] | [0.649, 1.044] | 6.50e-01 | 7.52e-01 | [0.436, 2.116] |
| Van Puyvelde, H.2023 | Methionine | highest vs. lowest | 8 | 24468/765041 | 0.958[0.911,1.008] | 0.958[0.911, 1.008] | [0.912, 1.071] | 9.61e-02 | 9.61e-02 | [0.9, 1.02] |
| Li, N.2022 | Whole grain intake | highest vs. lowest | 8 | 7853/211956 | 0.985[0.954,1.018] | 0.955[0.865, 1.054] | [0.808, 1.029] | 3.67e-01 | 3.59e-01 | [0.744, 1.225] |
| Adani, G.2020 | Dietary acrylamide intake | highest vs. lowest | 10 | 21173/585303 | 0.955[0.907,1.005] | 0.955[0.907, 1.005] | [0.761, 1.111] | 7.70e-02 | 7.70e-02 | [0.898, 1.014] |
| Aune, D.2012 | A-carotene | per 100ug /day | 6 | 9461/463721 | 0.948[0.908,0.99] | 0.955[0.898, 1.017] | [1.004, 1.782] | 1.66e-02 | 1.49e-01 | [0.81, 1.126] |
| Parra-Soto, S.2022 | Pescatarians | highest vs. lowest | 5 | 585/565969 | 0.956[0.873,1.047] | 0.953[0.842, 1.079] | [0.78, 1.04] | 3.37e-01 | 4.52e-01 | [0.683, 1.332] |
| Han, X.2022 | Dietary vitamin A | highest vs. lowest | 8 | 11532/1049423 | 0.953[0.885,1.027] | 0.953[0.885, 1.027] | [0.831, 1.201] | 2.04e-01 | 2.04e-01 | [0.868, 1.046] |
| Hu, F.2012 | Total b-carotene | highest vs. lowest | 9 | 25272/967169 | 0.951[0.899,1.006] | 0.951[0.899, 1.006] | [0.851, 1.27] | 8.12e-02 | 8.12e-02 | [0.889, 1.018] |
| Liu, F.2022 | Flavones | highest vs. lowest | 5 | 5615/424536 | 0.956[0.915,0.999] | 0.942[0.853, 1.04] | [0.912, 1.071] | 4.56e-02 | 2.36e-01 | [0.691, 1.284] |
| Buck, K.2010 | Lignans and enterolignans | highest vs. lowest | 3 | 4169/138768 | 0.936[0.848, 1.033] | 0.942[0.807, 1.1] | [0.794, 1.112] | 1.88e-01 | 4.51e-01 | [0.176, 5.045] |
| Chang, V. C.2019 | Total iron intake | highest vs. lowest | 2 | 4035/140961 | 0.883[0.812,0.96] | 0.94[0.733, 1.204] | [0.763, 0.921] | 3.69e-03 | 6.23e-01 | NA |
| Chen, H. 2018 | Dietary carrot intake | highest vs. lowest | 2 | 2615/96151 | 0.977[0.926,1.031] | 0.939[0.806, 1.093] | [0.667, 1.038] | 3.94e-01 | 4.13e-01 | NA |
| Yang, J.2023 | Isoflavone intake | highest vs. lowest | 7 | 21294/887179 | 0.955[0.897,1.017] | 0.937[0.858, 1.024] | [0.91, 1.1] | 1.50e-01 | 1.52e-01 | [0.765, 1.149] |
| Naghshi, Sina.2021 | Total nuts | highest vs. lowest | 3 | 5695/108924 | 0.937[0.851,1.032] | 0.937[0.851, 1.032] | [0.834, 1.053] | 1.88e-01 | 1.88e-01 | [0.501, 1.754] |
| Zeng, J.2020 | Vitamin B6 | highest vs. lowest | 13 | 25261/740062 | 0.941[0.889,0.995] | 0.932[0.867, 1.002] | [0.872, 1.032] | 3.38e-02 | 5.59e-02 | [0.805, 1.08] |
| Liu, X. O.2014 | Vegetable intake | highest vs. lowest | 2 | 262/917 | 0.909[0.681,1.212] | 0.909[0.681, 1.212] | [0.659, 1.23] | 5.15e-01 | 5.15e-01 | NA |
| Zhao, T. T.2019 | Soyfoods intake | highest vs. lowest | 6 | 5042/359842 | 0.923[0.852,1.001] | 0.906[0.814, 1.008] | [0.864, 1.123] | 5.22e-02 | 6.99e-02 | [0.709, 1.158] |
| Kazemi, A.2021 | Yogurt intake | per 200g /day | 6 | 35542/965070 | 0.943[0.9,0.987] | 0.905[0.785, 1.045] | [0.989, 1.269] | 1.23e-02 | 1.73e-01 | [0.6, 1.366] |
| Shin, S.2023 | Healthy Dietary pattern | highest vs. lowest | 3 | 1466/108218 | 0.892[0.752,1.059] | 0.892[0.752, 1.059] | [0.75, 1.23] | 1.92e-01 | 1.92e-01 | [0.295, 2.703] |
| Kazemi, A.2021 | Nut intake | per 28g /day | 5 | 6859/241198 | 0.946[0.9,0.995] | 0.892[0.763, 1.043] | [0.911, 1.011] | 3.23e-02 | 1.52e-01 | [0.602, 1.323] |
| Liu, X.2013 | Cruciferous vegetables intake | highest vs. lowest | 2 | 3947/135162 | 0.859[0.738,1.001] | 0.859[0.738, 1.001] | [0.744, 1.159] | 5.19e-02 | 5.19e-02 | NA |
| Markellos, C.2022 | Olive oil intake | highest vs. lowest | 3 | 10313/347278 | 0.996[0.948,1.047] | 0.665[0.284, 1.558] | [0.951, 1.051] | 8.82e-01 | 3.48e-01 | [0, 14064.909] |
| ***Imageological diagnosis*** | | | | | | | | | | |
| Bodewes, F. T. H.2022 | Breast density | highest vs. lowest | 3 | 1356/109050 | 2.905[2.609,3.235] | 2.889[2.569, 3.249] | [2.67, 3.37] | 3.75e-84 | 3.06e-70 | [1.246,6.702] |
| Qu, X.2013 | Bone mineral density | per 0.1 g/m2 | 7 | 2498/117866 | 1.201[1.138,1.268] | 1.22[1.131, 1.32] | [1.073, 1.251] | 3.59e-11 | 4.15e-0 | [1.024, 1.458] |
| Qu, X.2013 | Bone mineral density | highest vs. lowest | 6 | 2171/107925 | 1.493[1.309,1.703] | 1.822[1.335, 2.485 | [1.117, 1.53 | 2.28e-09 | 1.54e-0 | [0.72, 4.61 |
| Nagel, G.2017 | Bone mineral density | highest vs. lowest | 7 | 2274/111881 | 1.407[1.231,1.607] | 1.581[1.162, 2.151] | [1.118, 1.536] | 5.15e-07 | 3.56e-03 | [0.655, 3.815] |
| Chen, J. H.2019 | Bone mineral density | per 0.1 g/m2 | 9 | 2706/163711 | 0.847[0.756,0.948] | 0.905[0.685, 1.196] | [0.77, 1.055] | 4.07e-03 | 4.83e-01 | [0.38, 2.154] |
| ***Life behaviour risks*** | | | | | | | | | | |
| Warren, G. W2013 | Smoking | ever vs. never | 15 | 31198/1022298 | NA | 1.126[1.082, 1.172] | [1.019, 1.169] | NA | 5.58e-09 | [1.046, 1.214] |
| Urbano, T.2021 | Light exposure at night | highest vs. lowest | 7 | 42502/527558 | 1.118[1.072,1.166] | 1.118[1.072, 1.166] | [1.022, 1.181] | 1.94e-07 | 1.94e-07 | [1.058, 1.181] |
| Chan ,D. S.M.2019 | Physical activity | highest vs. lowest | 16 | 36251/1932512 | 0.882[0.846,0.92] | 0.882[0.846, 0.92] | [0.784, 0.968] | 3.94e-09 | 3.94e-09 | [0.843, 0.923] |
| Ramalho ,NM.2020 | Education level | highest vs. lowest | 18 | 194654/10233064 | 1.239[1.214,1.264] | 1.221[1.144, 1.303] | [1.139, 1.289] | 1.70e-96 | 2.19e-09 | [0.959, 1.554] |
| Zhou, J.2023 | Famine exposure | ever vs. never | 4 | 10844/185341 | 1.301[1.231,1.376] | 1.294[1.174, 1.425] | [0.818, 1.444] | 1.32e-20 | 1.99e-07 | [0.968, 1.729] |
| Warren, G.W.2013 | Smoking | ever vs. never | 15 | 31198/1022298 | 1.086[1.058,1.116] | \| 1.101[1.053, 1.152] \| [1.011, 1.111] \| \| --- \| --- \| | [1.011, 1.111] | 1.21e-09 | 2.90e-05 | [0.972, 1.247] |
| Chan, D.S.M.2019 | Physical activity | highest vs. lowest | 21 | 32415/1554423 | 0.908[0.873,0.944] | 0.885[0.835, 0.937] | [0.899, 1.029] | 1.43e-06 | 3.02e-05 | [0.758, 1.032] |
| Neilson, H.K. | Physical activity | highest vs. lowest | 27 | 67508/2158709 | 0.911[0.881,0.942] | 0.881[0.833, 0.932] | [0.892, 1.03] | 3.84e-08 | 1.02e-05 | [0.728, 1.067] |
| Chen, X.2016 | Physical activity | highest vs. lowest | 38 | 66697/2533901 | 0.881[0.857,0.905] | 0.874[0.843, 0.906] | [0.856, 1.107] | 5.73e-20 | 1.50e-13 | [0.78, 0.98] |
| Chan, D.S.M.2019 | Physical activity | highest vs. lowest | 10 | 26514/1816908 | 0.876[0.832,0.923] | 0.871[0.812, 0.935] | [0.759, 1.319] | 5.59e-07 | 1.31e-04 | [0.746, 1.017] |
| Hidayat, K.2020 | Physical activity | highest vs. lowest | 4 | 5684/171671 | 0.782[0.699,0.876] | 0.782[0.699, 0.876] | [0.679, 0.899] | 2.08e-05 | 2.08e-05 | [0.611, 1.003] |
| Armenta-Guirado, B. I.2023 | Lifestyle Quality Indices | highest vs. lowest | 17 | 43794/1170291 | 0.8[0.772,0.828] | 0.772[0.711, 0.838] | [0.795, 1.217] | 7.63e-36 | 7.47e-10 | [0.595, 1.002] |
| Xu, C.2022 | Negative Emotions | ever vs. never | 9 | 2080/129621 | 1.053[1,1.11] | 1.826[1.249, 2.668] | [0.968, 1.078] | 5.08e-02 | 1.87e-03 | [0.568, 5.869] |
| Weinmann, S.2022 | Flight attendants | ever vs. never | 4 | 680/22410 | 1.425[1.321,1.537] | 1.425[1.321, 1.537] | [1.324, 1.693] | 4.31e-20 | 4.31e-20 | [1.207, 1.683] |
| Khuder, S. A.2000 | Passive smoking | ever vs. never | 3 | 922/399080 | 1.32[1.091,1.597] | 1.32[1.091, 1.597] | [0.969, 1.64] | 4.34e-03 | 4.34e-03 | [0.384, 4.54] |
| Lee, J.2021 | Sedentary work | highest vs. lowest | 13 | 82317/3318327 | 1.114[1.05,1.182] | 1.141[1.04, 1.252] | [0.669, 1.487] | 3.58e-04 | 5.40e-03 | [0.899, 1.448] |
| Xiao, W.2022 | Occupational Exposure-organic solvents | ever vs. never | 7 | 27481/1291458 | 1.091[1.064,1.118] | 1.106[1.026, 1.192] | [1.07, 1.13] | 3.72e-12 | 8.30e-03 | [0.908, 1.347] |
| Kim, A. S.2018 | Passive smoking | ever vs. never | 5 | 318729/432689 | 1.085[1.021,1.153] | 1.085[1.021, 1.153] | [0.9, 1.18] | 8.68e-03 | 8.68e-03 | [0.983, 1.197] |
| Chong, F.2021 | Sedentary behavior | per 1h/day | 8 | 17048/462782 | 1.009[1.004,1.015] | 1.011[1, 1.023] | [1, 1.02] | 7.78e-04 | 4.73e-02 | [0.98, 1.044] |
| Hidayat, K.2020 | Physical activity at a young age | highest vs. lowest | 9 | 11879/450019 | 0.928[0.889,0.969] | 0.882[0.797, 0.976] | [0.773, 0.952] | 7.57e-04 | 1.46e-02 | [0.671, 1.159] |
| Hiller, T.W.R.2020 | Time in the Sun | highest vs. lowest | 4 | 4019/144807 | 0.841[0.785,0.9] | 0.849[0.726, 0.924] | [0.758, 0.958] | 6.34e-07 | 1.16e-03 | [0.498, 1.347] |
| Cong, X.2023 | TCDD | ever vs. never | 5 | 3950/306385 | 0.992[0.879,1.12] | 1.315[0.796, 2.173] | [0.077, 3.299] | 9.02e-01 | 2.85e-01 | [0.311, 5.557] |
| Chen, C.2014 | Passive smoking | ever vs. never | 2 | 462/1801 | 0.972[0.778,1.214] | 1.297[0.545, 3.088] | [0.693, 1.112] | 8.00e-01 | 5.56e-01 | NA |
| Lin, Y.2013 | Striking life events | ever vs. never | 3 | NA/NA | 1.09[1.032,1.152] | 1.127[0.991, 1.282] | [1.001, 1.141] | 2.03e-03 | 6.87e-02 | [0.288, 4.414] |
| Chong, F.2021 | Sedentary behavior | highest vs. lowest | 8 | 17048/462782 | 1.058[1.008,1.11] | 1.085[0.993, 1.185] | [0.969, 1.229] | 2.24e-02 | 7.07e-02 | [0.841, 1.4] |
| Jia, Y.2017 | Depression | ever vs. never | 11 | 73387/829498 | 0.998[0.932,1.069] | 1.078[0.932, 1.247] | [0.823, 1.022] | 9.59e-01 | 3.11e-01 | [0.73, 1.591] |
| Urbano, T.2021 | Light exposure at night | highest vs. lowest | 4 | 4258/265734 | 1.051[0.957,1.153] | 1.051[0.957, 1.153] | [0.84, 1.52] | 2.97e-01 | 2.97e-01 | [0.857, 1.289] |
| Kamdar, B.B.2013 | Long-term night-shift work | highest vs. lowest | 3 | 4510/266633 | 1.052[0.953,1.163] | 1.047[0.921, 1.19] | [0.82, 1.27] | 3.16e-01 | 4.83e-01 | [0.311, 3.524] |
| Yang, Y.2013 | Passive smoking | ever vs. never | 10 | 14831/782534 | 1.01[0.959,1.064] | 1.024[0.939, 1.117] | [0.88, 1.09] | 7.06e-01 | 5.92e-01 | [0.83, 1.263] |
| Kamdar, B.B.2013 | Short-term night-shift work | highest vs. lowest | 2 | 2069/188071 | 1.011[0.916,1.115] | 1.011[0.916, 1.115] | [0.871, 1.101] | 8.28e-01 | 8.28e-01 | NA |
| Wong, A.T.Y.2021 | Long sleep duration | highest vs. lowest | 15 | 47167/1365136 | 1.007[0.979,1.036] | 0.998[0.96, 1.039] | [0.983, 1.053] | 6.12e-01 | 9.38e-01 | [0.931, 1.07] |
| Wong, A.T.Y.2021 | Short sleep duration | highest vs. lowest | 15 | 60039/1365136 | 0.995[0.977,1.012] | 0.993[0.973, 1.014] | [0.978, 1.028] | 5.49e-01 | 5.15e-01 | [0.962, 1.026] |
| Van, N. T. H.2021 | Night-shift work | highest vs. lowest | 12 | 58896/2643241 | 0.981[0.945,1.019] | 0.982[0.943, 1.022] | [0.924, 1.053] | 3.31e-01 | 3.65e-01 | [0.926, 1.04] |
| Takkouche , B.2005 | Hair dye | ever vs. never | 2 | 1135/665993 | 0.947[0.894,1.002] | 0.981[0.85, 1.132] | [0.867, 0.982] | 5.71e-02 | 7.93e-01 | NA |
| Shi, Yun.2015 | Physical activity | highest vs. lowest | 3 | 16658/857030 | 0.963[0.865,1.071] | 0.967[0.848, 1.104] | [0.911, 1.409] | 4.82e-01 | 6.23e-01 | [0.292, 3.21] |
| Heikkilä, K.2013 | Work stress | highest vs. lowest | 11 | 1008/107212 | 0.967[0.82,1.141] | 0.967[0.82, 1.141] | [0.633, 1.191] | 6.92e-01 | 6.92e-01 | [0.799, 1.17] |
| Chen, Yuheng.2018 | Long sleep duration | highest vs. lowest | 8 | 22379/527674 | 0.964[0.899,1.034] | 0.959[0.868, 1.059] | [0.77, 1.03] | 3.07e-01 | 4.06e-01 | [0.752, 1.222] |
| Chen, Yuheng.2018 | Short sleep duration | highest vs. lowest | 8 | 22379/527674 | 0.959[0.914,1.006] | 0.952[0.894, 1.014] | [0.713, 0.982] | 8.84e-02 | 1.27e-01 | [0.839, 1.08] |
| Chan, D. S. M.2019 | Walking | highest vs. lowest | 4 | 7250/188660 | 0.948[0.865,1.039] | 0.948[0.865, 1.039] | [0.822, 1.141] | 2.54e-01 | 2.54e-01 | [0.775, 1.16] |
| ***Environment*** | | | | | | | | | | |
| Wei, W.2021 | NO2 | per 10 µg/m3 | 11 | 126083/4083012 | 1.016[1.008,1.023] | 1.023[1.008, 1.039] | [1, 1.02] | 1.87e-05 | 2.48e-03 | [0.982, 1.066] |
| Wei, W.2021 | PM10 | per 10 µg/m3 | 6 | 24941/1392824 | 0.991[0.977,1.005] | 1.04[0.983, 1.1] | [1.087, 1.308] | 2.18e-01 | 1.77e-01 | [0.872, 1.239] |
| Wei, W.2021 | PM2.5 | per 10 µg/m3 | 10 | 1591297/4569594 | 1.023[0.998,1.048] | 1.027[0.99, 1.065] | [0.988, 1.048] | 6.69e-02 | 1.57e-01 | [0.957, 1.101] |
| Praud, D.2023 | NO2 | per 10 µg/m3 | 7 | 119536/4815997 | 1.011[1.003,1.02] | 1.013[0.999, 1.027] | [1, 1.02] | 8.99e-03 | 6.62e-02 | [0.981, 1.046] |
| Hiller, T. W. R.2020 | UVR | highest vs. lowest | 6 | 15941/399720 | 1.01[0.968,1.053] | 1.013[0.932, 1.102] | [0.971, 1.091] | 6.48e-01 | 7.55e-01 | [0.802, 1.28] |
| ***Past gynaecological history*** | | | | | | | | | | |
| Li, C.2021 | Parity | ever vs. never | 3 | 5278/2464049 | 0.903[0.824,0.991] | 0.903[0.824, 0.991] | [0.793, 1.191] | 3.14e-02 | 3.14e-02 | [0.496, 1.646] |
| Ji, L. W.2019 | Age at first use of oral contraceptives | highest vs. lowest | 10 | 15093/1635060 | 1.257[1.118,1.414] | 1.174[0.898, 1.535] | [0.742, 1.329] | 1.33e-04 | 2.41e-01 | [0.504, 2.736] |
| Deng, Y.2018 | IA | ever vs. never | 24 | 17862/69061 | 1.047[1.007,1.09] | 1.08[0.982, 1.187] | [0.677, 0.837] | 2.25e-02 | 1.12e-01 | [0.703, 1.659] |
| Guo, J.2015 | Spontaneous abortion | ever vs. never | 12 | 18452/1089413 | 1.022[0.983,1.063] | 1.017[0.967, 1.07] | [0.994, 1.144] | 2.72e-01 | 5.13e-01 | [0.913, 1.133] |
| ***Anthropometric indices*** | | | | | | | | | | |
| Neil-Sztramko, S. E.2017 | BMI>=25 | high vs. low, postmenopausal | 18 | 28671/1156200 | 0.858[0.808,0.912] | 0.858[0.808, 0.912] | [0.77, 0.96] | 6.53e-07 | 6.53e-07 | [0.804, 0.916] |
| Hidayat, K.2018 | BMI iya | per 5 kg/m2 | 24 | 33606/2000548 | 0.957[0.945,0.968] | 0.86[0.819, 0.903] | [0.923, 0.973] | 8.39e-13 | 1.30e-09 | [0.718, 1.03] |
| Byun, D.2022 | BMI | per 5 kg/m2 | 21 | 39733/1849775 | 0.864[0.849,0.879] | 0.844[0.811, 0.878] | [0.89, 0.97] | 2.64e-61 | 5.77e-17 | [0.737, 0.966] |
| Namazi, N.2019 | Fat mass | highest vs. lowest | 7 | 5956/265841 | 1.44[1.332,1.555] | 1.445[1.226, 1.703] | [1.43, 1.79] | 2.83e-20 | 1.16e-05 | [0.883, 2.364] |
| Hao, Y.2021 | Weight gain | highest vs. lowest | 16 | 20536/864957 | 1.392[1.318,1.471] | 1.415[1.273, 1.574] | [0.549, 1.11] | 2.44e-32 | 1.35e-10 | [0.996, 2.012] |
| Chen, Y.2017 | BMI | highest vs. lowest | 28 | 51544/5048361 | 1.289[1.238,1.342] | 1.209[1.083, 1.35] | [0.712, 0.972] | 1.71e-34 | 7.16e-04 | [0.741, 1.973] |
| Neil-Sztramko, S. E.2017 | BMI <25 | high vs. low, postmenopausal | 18 | 29860/1221731 | 0.884[0.836,0.934] | 0.853[0.785, 0.927] | [0.867, 1.047] | 1.21e-05 | 1.80e-04 | [0.674, 1.078] |
| Xue, F.2007 | Birth length | highest vs. lowest | 3 | 612/6112 | 1.708[1.266,2.304] | 1.708[1.266, 2.304] | [0.808, 2.429] | 4.63e-04 | 4.63e-04 | [0.245, 11.904] |
| Dehesh, T.2023 | BMI >=30 | highest vs. lowest | 12 | 20197/1221332 | 1.163[1.105,1.226] | 1.22[1.027, 1.45] | [0.766, 1.017] | 1.16e-08 | 2.35e-02 | [0.677, 2.2] |
| Xu, X.2009 | Birth weight | highest vs. lowest | 4 | 3638/3340062 | 1.192[1.048,1.355] | 1.192[1.048, 1.355] | [1.023, 1.332] | 7.37e-03 | 7.37e-03 | [0.899, 1.579] |
| Liu, K.2018 | BMI | per 5 kg/m2 | 12 | 19480/2282874 | 1.021[1.015,1.028] | 1.025[1.008, 1.043] | [1.02, 1.04] | 6.04e-10 | 4.77e-03 | [0.97, 1.083] |
| Chan, D. S. M.2019 | Weight loss | highest vs. lowest | 16 | 19351/1035071 | 0.896[0.834,0.963] | 0.896[0.834, 0.963] | [0.63, 1.114] | 2.68e-03 | 2.68e-03 | [0.828, 0.969] |
| Neil-Sztramko, S. E.2017 | BMI >=30 | highest vs. lowest | 4 | 5357/319784 | 1.113[0.867,1.429] | 1.113[0.867, 1.429] | [0.89, 2.63] | 4.00e-01 | 4.00e-01 | [0.643, 1.926] |
| Zhou, W.2020 | Birth weight | highest vs. lowest | 8 | 11068/384250 | 1.078[0.982,1.183] | 1.107[0.945, 1.297] | [0.809, 1.189] | 1.17e-01 | 2.09e-01 | [0.754, 1.625] |
| Xu, X.2009 | Birth weight | per 1 kg | 5 | 10168/3728138 | 1.049[0.999,1.101] | 1.049[0.964, 1.142] | [1.01, 1.2] | 5.45e-02 | 2.71e-01 | [0.841, 1.308] |
| Amadou, A.2013 | Waist-to-hip ratio | per 0.1 | 3 | 1245/136148 | 1.018[0.97,1.069] | 1.018[0.97, 1.069] | [0.929, 1.099] | 4.63e-01 | 4.63e-01 | [0.745, 1.392] |
| Amadou, A.2013 | Height | per 10 cm | 6 | 2830/224123 | 1.016[0.998,1.035] | 1.016[0.998, 1.035] | [0.981, 1.081] | 8.19e-02 | 8.19e-02 | [0.991, 1.042] |
| ***Biomarkers*** | | | | | | | | | | |
| Key, T. J.2010 | IGF1 concentrations | highest vs. lowest | 16 | 4790/14218 | 1.25[1.124,1.391] | 1.25[1.124, 1.391] | [1.065, 1.675] | 4.06e-05 | 4.06e-05 | [1.112, 1.405] |
| Amerizadeh, A.2022 | Serum TG levels | highest vs. lowest | 11 | 18793/862537 | 0.948[0.906,0.992] | 0.948[0.906, 0.992] | [0.761, 1.111] | 2.17e-02 | 2.17e-02 | [0.899, 0.999] |
| Nouri, M.2022 | Serum TG levels | highest vs. lowest | 9 | 14567/722211 | 0.935[0.879,0.994] | 0.935[0.879, 0.994] | [0.8, 1.13] | 3.27e-02 | 3.27e-02 | [0.868, 1.007] |
| Ren, X.2020 | Plasma folate | highest vs. lowest | 2 | 815/19939 | 1.19[0.908,1.558] | 1.631[0.609, 4.369] | [0.849, 1.479] | 2.07e-01 | 3.31e-01 | NA |
| Renehan, A. G.2004 | IGF1 concentrations | highest vs. lowest | 4 | 927/2852 | 1.265[0.958,1.67] | 1.265[0.958, 1.67] | [0.838, 1.985] | 9.81e-02 | 9.81e-02 | [0.687, 2.329] |
| Renehan, A. G.2004 | IGFBP-3 | highest vs. lowest | 3 | 546/1883 | 1.193[0.849,1.675] | 1.193[0.849, 1.675] | [0.732, 1.766] | 3.09e-01 | 3.09e-01 | [0.132, 10.794] |
| Chang, V. C.2019 | Serum/plasma iron | highest vs. lowest | 3 | 4231/377740 | 1.207[1.079,1.349] | 1.364[1.012, 1.838] | [0.993, 1.273] | 9.67e-04 | 4.18e-02 | [0.05, 37.523] |
| Song, D.2019 | Blood vitamin D | highest vs. lowest | 6 | 2221/83711 | 1.178[1.038,1.338] | 1.17[0.927, 1.479] | [1.021, 1.361] | 1.13e-02 | 1.87e-01 | [0.675, 2.029] |
| Lou, M. W. C.2023 | CRP | highest vs. lowest | 9 | 6553/256083 | 1.079[0.996,1.168] | 1.138[0.984, 1.315] | [0.9, 1.201] | 6.20e-02 | 8.10e-02 | [0.751, 1.723] |
| Wang, M.2016 | Plasma prolactin levels | highest vs. lowest | 6 | 6283/12173 | 1.174[1.051,1.311] | 1.174[1.051, 1.311] | [0.99, 1.29] | 4.49e-03 | 4.49e-03 | [1.004, 1.373] |
| Walker, K.2011 | Circulating E2 levels | highest vs. lowest | 7 | 693/2302 | 1.083[0.968,1.213] | 1.103[0.96, 1.268] | [0.861, 1.181] | 1.65e-01 | 1.67e-01 | [0.831, 1.464] |
| Ren, X.2020 | Plasma folate | per 5ng/ml | 2 | 815/19939 | 1.029[0.981,1.08] | 1.105[0.897, 1.36] | [0.971, 1.071] | 2.40e-01 | 3.48e-01 | NA |
| Song, D.20119 | Blood vitamin D | per 5 nmol/L | 4 | 1925/72844 | 0.998[0.982,1.015] | 1.007[0.96, 1.056] | [1.003, 1.053] | 8.49e-01 | 7.69e-01 | [0.813, 1.248] |
| Nouri, M.2022 | HDL–C | highest vs. lowest | 8 | 10699/445749 | 0.998[0.906,1.1] | 0.998[0.906, 1.1] | [0.858, 1.289] | 9.69e-01 | 9.69e-01 | [0.884, 1.127] |
| Ma, H. Q.2016 | Serum triglycerides | highest vs. lowest | 8 | 12177/594751 | 0.936[0.874,1.003] | 0.987[0.859, 1.134] | [0.761, 1.111] | 6.01e-02 | 8.52e-01 | [0.699, 1.393] |
| Nouri, M.2022 | Serum TC levels | highest vs. lowest | 13 | 26260/1083693 | 1.006[0.971,1.042] | 0.976[0.894, 1.065] | [0.608, 0.809] | 7.46e-01 | 5.81e-01 | [0.748, 1.272] |
| Xu, J.2017 | Urinary 6-sulfatoxymelatonin levels | highest vs. lowest | 6 | 1824/5778 | 0.974[0.879,1.079] | 0.968[0.848, 1.105] | [0.893, 1.252] | 6.17e-01 | 6.32e-01 | [0.699, 1.34] |
| Amerizadeh, A.2022 | HDL–C | highest vs. lowest | 8 | 9248/378200 | 0.964[0.903,1.029] | 0.967[0.86, 1.088] | [0.858, 1.289] | 2.74e-01 | 5.81e-01 | [0.737, 1.27] |
| Amerizadeh, A.2022 | Serum TC levels | highest vs. lowest | 14 | 36050/1861168 | 0.962[0.927,0.999] | 0.962[0.881, 1.05] | [1.03, 1.33] | 4.46e-02 | 3.83e-01 | [0.716, 1.291] |
| Nouri, M.2022 | Serum apolipoprotein A levels | highest vs. lowest | 3 | 8770/308319 | 1.04[0.923,1.171] | 0.957[0.703, 1.303] | [0.901, 1.291] | 5.21e-01 | 7.80e-01 | [0.023, 39.338] |
| Amerizadeh, A2022. | LDL–C | highest vs. lowest | 6 | 8046/291988 | 0.94[0.88,1.004] | 0.94[0.88, 1.004] | [0.75, 1.13] | 6.62e-02 | 6.62e-02 | [0.856, 1.032] |
| Wong, A. T. Y.2021 | Urinary aMT6s | highest vs. lowest | 7 | 2296/6790 | 0.88[0.793,0.976] | 0.889[0.779, 1.015] | [0.643, 1.282] | 1.59e-02 | 8.15e-02 | [0.678, 1.165] |
| Nouri, M.2022 | HDL–C | per 1 mmol/L | 5 | 2255/90233 | 0.937[0.805,1.09] | 0.862[0.668, 1.113] | [0.558, 1.149] | 3.99e-01 | 2.55e-01 | [0.387, 1.923] |
| Han, X.2022 | Circulating high levels of vitamin A | highest vs. lowest | 3 | 1312/7692 | 0.81[0.641,1.024] | 0.8[0.598, 1.07] | [0.678, 1.479] | 7.77e-02 | 1.33e-01 | [0.058, 10.965] |
| ***Use of medical/hormonal therapy*** | | | | | | | | | | |
| Karasneh, R. A.2017 | Cardiac glycosides use | ever vs. never | 6 | 53971/2339718 | 1.388[1.327,1.452] | 1.388[1.327, 1.452] | [1.321, 1.461] | 7.36e-46 | 7.36e-46 | [1.302, 1.48] |
| Thakur, A. A.20118 | Calcium channel blockers | ever vs. never | 3 | 2697/166459 | 1.306[1.207,1.412] | 1.306[1.207, 1.412] | [1.21, 1.42] | 3.02e-11 | 3.02e-11 | [0.784, 2.174] |
| Simin, J.2020 | Antibiotic use | ever vs. never | 2 | 18678/2158968 | 1.132[1.093,1.172] | 1.132[1.093, 1.172] | [1.09, 1.17] | 5.55e-12 | 5.55e-12 | NA |
| Li, Y. Y.2020 | Bisphosphonates | ever vs. never | 7 | 16404/905330 | 0.87[0.822,0.92] | 0.87[0.811, 0.933] | [0.799, 0.929] | 1.39e-06 | 1.02e-04 | [0.762, 0.995] |
| Leung, J. C. N.2022 | Antipsychotic use | ever vs. never | 4 | 2293/826036 | 1.228[1.143,1.319] | 1.388[1.114, 1.729] | [1.14, 1.6] | 1.80e-08 | 3.50e-03 | [0.569, 3.387] |
| Gao, Z.2022 | Antipsychotic use | ever vs. never | 4 | 18005/684713 | 1.388[1.273,1.514] | 1.374[1.082, 1.744] | [1.199, 1.499] | 1.13e-13 | 9.17e-03 | [0.476, 3.964] |
| Zhuang, Y.2022 | Antidepressant use | ever vs. never | 7 | 45809/1382755 | 1.134[1.099,1.171] | 1.093[1.011, 1.182] | [1.119, 1.289] | 3.24e-15 | 2.51e-02 | [0.88, 1.359] |
| Bakierzynska, M.2023 | Aspirin intake | ever vs. never | 28 | 60709/2533260 | 0.947[0.926,0.969] | 0.909[0.854, 0.966] | [1.04, 1.25] | 2.95e-06 | 2.16e-03 | [0.683, 1.209] |
| Peng, R.2020 | Bisphosphonates | ever vs. never | 7 | 14341/690131 | 0.882[0.83,0.936] | 0.891[0.81, 0.982] | [0.799, 0.949] | 4.04e-05 | 1.93e-02 | [0.698, 1.138] |
| Chen, Y.2023 | Thiazolidinediones | ever vs. never | 5 | 4340/1594865 | 0.878[0.804,0.959] | 0.878[0.804, 0.959] | [0.787, 0.968] | 3.81e-03 | 3.81e-03 | [0.761, 1.013] |
| Chen, Y.2023 | Insulins | ever vs. never | 11 | 20590/5501210 | 0.934[0.899,0.97] | 0.838[0.747, 0.94] | [0.892, 1.052] | 4.22e-04 | 2.62e-03 | [0.6, 1.17] |
| Yang, J,2023 | Beta blockers | ever vs. never | 9 | 11494/326801 | 1.087[1.032,1.145] | 1.136[0.879, 1.469] | [0.894, 1.026] | 1.66e-03 | 3.30e-01 | [0.472, 2.738] |
| Ni, X. J.2012 | Postmenopausal hormone therapy | ever vs. never | 2 | 737/423482 | 1.001[0.916,1.094] | 1.126[0.796, 1.593] | [0.892, 1.072] | 9.82e-01 | 5.03e-01 | NA |
| Ni, H.2017 | Diuretic use | ever vs. never | 6 | 86306/3013478 | 1.086[1.044,1.129] | 1.076[0.984, 1.176] | [1.119, 1.269] | 4.44e-05 | 1.09e-01 | [0.821, 1.41] |
| Chen, Y.2023 | Insulin secretagogues | ever vs. never | 4 | 1056/216914 | 1.04[0.91,1.189] | 1.039[0.903, 1.196] | [0.788, 1.635] | 5.64e-01 | 5.91e-01 | [0.729, 1.482] |
| Undela, K.2012 | Statin use | ever vs. never | 13 | 31009/2044142 | 1.009[0.98,1.04] | 1.008[0.975, 1.043] | [0.978, 1.109] | 5.37e-01 | 6.29e-01 | [0.953, 1.067] |
| Du, X.2012 | Insulin glargine | ever vs. never | 5 | 1137/201857 | 1.005[0.797,1.268] | 1.005[0.797, 1.268] | [0.77, 1.57] | 9.66e-01 | 9.66e-01 | [0.689, 1.466] |
| Santucci, C.2021 | Aspirin intake | ever vs. never | 20 | 36624/1690197 | 0.984[0.957,1.01] | 0.964[0.907, 1.024] | [1.04, 1.25] | 2.29e-01 | 2.34e-01 | [0.766, 1.213] |
| Qiao, Y.2018 | Aspirin intake | ever vs. never | 20 | 37550/1192249 | 0.968[0.945,0.992] | 0.963[0.912, 1.017] | [0.928, 1.169] | 8.71e-03 | 1.72e-01 | [0.79, 1.174] |
| Tang, G. H.2018 | Metformin | ever vs. never | 11 | 2581/496817 | 0.951[0.915,0.988] | 0.936[0.842, 1.041] | [0.766, 1.214] | 9.76e-03 | 2.24e-01 | [0.712, 1.231] |
| Du, R.2018 | Thiazolidinedione | ever vs. never | 7 | 3019/271458 | 0.925[0.862,0.992] | 0.82[0.664, 1.013] | [0.882, 1.131] | 2.92e-02 | 6.58e-02 | [0.455, 1.479] |
| Ni, H.2017 | Angiotensin-converting enzyme inhibitor / angiotensin-receptor | ever vs. never | 9 | 32316/766811 | 0.943[0.882,1.009] | 0.948[0.88, 1.021] | [0.829, 1] | 8.94e-02 | 1.61e-01 | [0.844, 1.065] |
| Indrakusuma, Aabp.2022 | Antipsychotic use | ever vs. never | 2 | 5572/536738 | 1.293[1.159,1.444] | 0.753[0.224, 2.529] | [1.199, 1.499] | 4.61e-06 | 6.46e-01 | NA |
| Chen, Y. | Biguanides | ever vs. never | 6 | 14373/3567122 | 0.461[0.445,0.478] | 0.691[0.389, 1.23] | [0.363, 0.392] | 0.00e+00 | 2.09e-01 | [0.083, 5.756] |
| ***Pre-existing medical conditions and interventions*** | | | | | | | | | | |
| Yao, X.2023 | Atrial fibrillation | ever vs. never | 5 | 11632/442702 | 1.179[1.139,1.221] | 1.179[1.139, 1.221] | [1.111, 1.211] | 1.36e-20 | 1.36e-20 | [1.115, 1.248] |
| Hardefeldt, P. J.2012 | Antibody | ever vs. never | 8 | 1687/7727 | 2.024[1.636,2.504] | 2.522[1.666, 3.816] | [1.104, 3.523] | 8.46e-11 | 1.21e-05 | [0.747, 8.517] |
| Wu, D.2023 | Obstructive sleep apnea | ever vs. never | 9 | 193316/2775494 | 1.666[1.644,1.687] | 1.582[1.265, 1.978] | [1.828, 2.579] | 0.00e+00 | 5.67e-05 | [0.72, 3.476] |
| Shi, T.2018 | Periodontal disease | ever vs. never | 7 | 3471/169754 | 1.184[1.11,1.263] | 1.203[1.094, 1.322] | [1.111, 1.361] | 2.74e-07 | 1.42e-04 | [0.994, 1.455] |
| Guo, M.2019 | Metabolic syndrome | ever vs. never | 17 | 25569/118295 | 1.043[1.006,1.081] | 1.198[1.08, 1.328] | [0.904, 0.992] | 2.13e-02 | 6.25e-04 | [0.824, 1.74] |
| Tran, T. V.2023 | Hyperthyroidism | ever vs. never | 12 | 16289/4336992 | 1.127[1.088,1.162] | 1.139[1.075, 1.207] | [1.118, 1.358] | 2.86e-12 | 1.02e-05 | [1.004, 1.292] |
| Wilson, R. B.2023 | Bariatric surgery | ever vs. never | 13 | 23218/2173832 | 0.567[0.545,0.59] | 0.558[0.436, 0.715] | [0.378, 0.428] | 7.16e-175 | 4.12e-06 | [0.208, 1.497] |
| Hardefeldt, P. J.2012 | Autoimmune thyroiditis | ever vs. never | 4 | 646/1085 | 2.923[2.133,4.005] | 2.923[2.133, 4.005] | [1.779, 4.1] | 2.46e-11 | 2.46e-11 | [1.464, 5.836] |
| Hardefeldt, P. J.2012 | Goitre | ever vs. never | 4 | 549/1009 | 2.824[2.037,3.914] | 2.824[2.037, 3.914] | [1.021, 4.87] | 4.61e-10 | 4.61e-10 | [1.379, 5.783] |
| Wei, L.2021 | Sleep-disordered breathing | ever vs. never | 8 | 8300/1398113 | 1.017[0.988,1.046] | 1.357[1.077, 1.709] | [0.93, 0.99] | 2.52e-01 | 9.68e-03 | [0.615, 2.994] |
| Shao, J.2018 | Periodontal disease | ever vs. never | 11 | 3953/173162 | 1.175[1.105,1.249] | 1.219[1.064, 1.396] | [1.032, 1.232] | 2.85e-07 | 4.33e-03 | [0.875, 1.698] |
| Xiong, F.2023 | Diabetes | ever vs. never | 46 | 241406/17326476 | 1.313[1.299,1.326] | 1.155[1.04, 1.282] | [1.106, 1.15] | 0.00e+00 | 7.10e-03 | [0.594, 2.244] |
| Chen, S.2021 | Hypothyroidism | ever vs. never | 5 | 5127/276031 | 0.953[0.909,1] | 0.953[0.909, 1] | [0.919, 1.069] | 4.85e-02 | 4.85e-02 | [0.882, 1.03] |
| Chen, Hsin-Hao.2023 | CAD | ever vs. never | 5 | 19075/1109286 | 0.899[0.845,0.956] | 0.87[0.776, 0.975] | [0.641, 0.9] | 7.27e-04 | 1.65e-02 | [0.596, 1.269] |
| Ishihara, B. P.2020 | Bariatric surgery | ever vs. never | 6 | 4896/334592 | 0.519[0.457,0.589] | 0.509[0.312, 0.832] | [0.433, 0.642] | 2.61e-24 | 7.06e-03 | [0.09, 2.88] |
| Yap, D. W. T.2022 | Obstructive sleep apnea | ever vs. never | 5 | 39969/5160124 | 0.992[0.968,1.017] | 1.308[0.981, 1.744] | [0.928, 0.978] | 5.42e-01 | 6.79e-02 | [0.426, 4.015] |
| Chen, S.2021 | Hyperthyroidism | ever vs. never | 2 | 1144/62488 | 1.117[1.073,1.163] | 1.26[0.906, 1.753] | [1.068, 1.158] | 8.11e-08 | 1.70e-01 | NA |
| Zheng, X.2022 | Heart failure | ever vs. never | 5 | 29064/2131777 | 1.157[1.099,1.218] | 1.226[0.919, 1.635] | [0.898, 1.029] | 2.27e-08 | 1.65e-01 | [0.409, 3.673] |
| Vojtechova, P.2009 | Atopy | ever vs. never | 4 | 1205/16258 | 1.085[0.914,1.287] | 1.177[0.894, 1.548] | [1.016, 3.559] | 3.52e-01 | 2.45e-01 | [0.442, 3.134] |
| Wang, T.2015 | Parkinson’s disease | ever vs. never | 7 | 2032/204032 | 1.047[0.971,1.13] | 1.094[0.91, 1.316] | [0.864, 1.151] | 2.33e-01 | 3.37e-01 | [0.648, 1.85] |
| Wang, Y.2020 | GDM | ever vs. never | 9 | 81368/1765235 | 1.051[0.987,1.118] | 1.082[0.889, 1.318] | [1.09, 1.39] | 1.22e-01 | 4.32e-01 | [0.54, 2.17] |
| Bonifazi, M.2013 | Systemic sclerosis | ever vs. never | 7 | 74/5256 | 1.056[0.843,1.324] | 1.056[0.843, 1.324] | [0.764, 2.404] | 6.34e-01 | 6.34e-01 | [0.786, 1.42] |
| Seretis, A.2019 | Hypertension | ever vs. never | 11 | 10479/398641 | 1.117[1.083,1.152] | 1.053[0.981, 1.131] | [0.972, 1.152] | 1.97e-12 | 1.54e-01 | [0.864, 1.284] |
| Vojtechova, P.2009 | Hay fever | ever vs. never | 6 | 7758/1156872 | 1.026[0.946,1.112] | 1.043[0.939, 1.159] | [0.912, 1.092] | 5.37e-01 | 4.33e-01 | [0.859, 1.267] |
| Ye, J.2022 | Endometriosis | ever vs. never | 11 | 65711/3850398 | 1.02[0.994,1.047] | 1.037[0.988, 1.088] | [0.963, 1.073] | 1.30e-01 | 1.44e-01 | [0.914, 1.176] |
| Vojtechova, P.2009 | Allergy | ever vs. never | 7 | 6794/1152686 | 1.009[0.944,1.078] | 1.009[0.944, 1.078] | [0.919, 1.069] | 8.02e-01 | 8.02e-01 | [0.924, 1.1] |
| Peng, C.2023 | Migraine | ever vs. never | 4 | 61084/374220 | 0.98[0.93,1.031] | 0.987[0.899, 1.083] | [0.886, 1.207] | 4.35e-01 | 7.82e-01 | [0.669, 1.456] |
| Najdi, N.2022 | Tubal ligation | ever vs. never | 6 | 70915/1520608 | 0.987[0.968,1.006] | 0.979[0.939, 1.021] | [0.784, 1.239] | 1.66e-01 | 3.19e-01 | [0.893, 1.073] |
| Tran, T. V.2023 | Hypothyroidism | ever vs. never | 10 | 20856/752661 | 0.951[0.921,0.982] | 0.964[0.903, 1.028] | [0.842, 1.022] | 2.25e-03 | 2.64e-01 | [0.811, 1.145] |
| Hassan, H.2023 | Hysterectomy with BSO | ever vs. never | 8 | 79005/1136999 | 0.943[0.907,0.98] | 0.944[0.842, 1.059] | [0.849, 0.999] | 2.71e-03 | 3.26e-01 | [0.645, 1.381] |
| Sun, M.2018 | PE | ever vs. never | 8 | 31983/1970690 | 0.889[0.836,0.946] | 0.934[0.825, 1.057] | [0.789, 0.939] | 1.93e-04 | 2.77e-01 | [0.66, 1.32] |
| Vojtechova, P.2009 | Asthma | ever vs. never | 10 | 9788/1320815 | 0.808[0.763,0.856] | 0.929[0.727, 1.188] | [0.921, 1.311] | 3.55e-13 | 5.58e-01 | [0.409, 2.113] |
| Li, Z.2022 | Polycystic ovary syndrome | ever vs. never | 4 | 23710/3546883 | 0.92[0.737,1.148] | 0.92[0.737, 1.148] | [0.64, 1.13] | 4.59e-01 | 4.59e-01 | [0.566, 1.496] |
| Sergentanis, T. N.2014 | IVF | ever vs. never | 6 | 14055/1445904 | 0.892[0.788,1.009] | 0.907[0.74, 1.112] | [0.618, 0.939] | 6.99e-02 | 3.48e-01 | [0.521, 1.579] |
| Heting, M.2023 | Levonorgestrel intrauterine system | ever vs. never | 3 | 4647/190475 | 0.809[0.744,0.881] | 0.796[0.567, 1.119] | [0.821, 1.03] | 1.01e-06 | 1.89e-01 | [0.011, 57.564] |
| ***Congenital factor*** | | | | | | | | | | |
| Xue, F.2007 | Paternal age | per 15 years | 3 | 15853/150312 | 1.085[1.008,1.167] | 1.085[1.008, 1.167] | [1.007, 1.228] | 3.03e-02 | 3.03e-02 | [0.674, 1.746] |
| Xue, F.2007 | Twin membership  (including monozygotic and dizygotic) | yes vs. no | 2 | 433/175924 | 0.918[0.856,0.986] | 0.918[0.856, 0.986] | [0.822, 1.002] | 1.88e-02 | 1.88e-02 | NA |

**Abbreviation：**ACEI/ARB : Angiotensin-converting enzyme inhibitor / angiotensin-receptor；BMI, body mass index; BMI iya, body mass index in young adulthood; CI, confidence interval; HRT, hormone replacement therapy; PA, physical activity; PoMP, postmenopausal; ELF-EMFs: extremely low-frequency electromagnetic fields；DII, dietary inflammatory index; SSBs, sugar-sweetened beverages; SFA, saturated fatty acids ;MUFA, monounsaturated fatty acids; PUFA, polyunsaturated fatty acids; ASBs, artificially sweetened beverages ;UVR, ultraviolet radiation; IA, Induced abortion;IGF-1,Insulin-like growth factor 1 ;IGFBP-3,IGF binding protein 3TG,total triglycerides; TC, total cholesterol；CRP,C-reactive protein; CAD, coronary artery disease; GDM, Gestational diabetes mellitus ;PE, Preeclampsia ;BSO, bilateral salpingo-oophorectom; NA, not available;

**Supplemental Table 4:** Evaluation of heterogeneity, small study effects and excess significance bias in the 281 meta-analyses investigating risk factors associated with breast cancer incidence - only cohort studies included.

| **Author, year** | **Exposure** | **Exposure contrast** | **Egger’s P** | **I^2^** | **Studies** | **Excess P-value** |
| --- | --- | --- | --- | --- | --- | --- |
|  |  |  |  |  |  |  |
| ***Dietary intake*** | | | | | | |
| Farvid, M. S.2020 | Fiber intake | highest vs. lowest | 0.224 | 6.124 | 17 | 0.699 |
| Zhang, L.2019 | Vegetable-fruit-soybean dietary pattern | highest vs. lowest | 0.725 | 0 | 12 | 0.623 |
| Shin, S.2023 | Alcohol | highest vs. lowest | 0.341 | 0 | 4 | 0.239 |
| Kazemi, A.2021 | Total meat intake | per 100 g/day | 0.001 | 70.622 | 25 | 0.000 |
| Li, N.2022 | Fiber intake | highest vs. lowest | 0.169 | 29.63 | 20 | 0.211 |
| Cai, X.2016 | Selenium | highest vs. lowest | 0.815 | 0 | 12 | 0.461 |
| Wang, Q.2020 | Tofu intake | highest vs. lowest | 0.029 | 57.336 | 14 | 0.096 |
| Malcomson, F. C.2023 | Adherence score | highest vs. lowest | 0.889 | 65.023 | 7 | 0.666 |
| Li, D.Y.2018 | DII | per 1 | 0.343 | 87.333 | 6 | 0.012 |
| Chen, J.Y.2016 | Wine Drinking | highest vs. lowest | 0.799 | 56.734 | 9 | 0.025 |
| Kazemi, A.2021 | Processed meat intake | per 50g /day | 0.170 | 63.227 | 17 | 0.079 |
| Pan, B.2023 | SSBs | per 250ml/day | 0.037 | 64.874 | 7 | 0.502 |
| Cao, Y.2016 | Total fat intake | highest vs. lowest | 0.026 | 46.568 | 20 | 0.001 |
| Anderson,J.J.2018 | Processed meat intake | highest vs. lowest | 0.137 | 69.121 | 8 | 0.203 |
| Rezaianzadeh, A.2018 | Red meat intake | highest vs. lowest | 0.240 | 7.301 | 8 | 0.026 |
| Farvid, M. S.2021 | Red meat intake | highest vs. lowest | 0.260 | 53.111 | 14 | 0.520 |
| Farvid, M. S.2021 | Processed meat intake | highest vs. lowest | 0.998 | 38.169 | 16 | 0.183 |
| Turati, F.2015 | Glycemic index | highest vs. lowest | 0.903 | 0 | 13 | 0.325 |
| Long, T.2022 | Glycemic index/Glycemic load | highest vs. lowest | 0.575 | 0 | 14 | 0.655 |
| Schlesinger, S.2017 | Glycemic index | per 10 units/day | 0.387 | 25.27 | 10 | 0.089 |
| Kazemi, A.2021 | Vegetable intake | per 100g /day | 0.001 | 58.911 | 14 | 0.001 |
| Kazemi, A.2021 | Fruit intake | per 100 g/day | 0.042 | 66.222 | 15 | 0.841 |
| Kazemi, A.2021 | Soy intake | per 30g /day | 0.590 | 0 | 7 | 0.497 |
| Wei, Y.2020 | Soy isoflavone | per 10 mg/day | 0.649 | 15.368 | 9 | 0.432 |
| Aune, D.2012 | Fruits and vegetables intake | per 200g /day | 0.316 | 0 | 6 | 0.822 |
| Li, Y. 2021 | Coffee intake | highest vs. lowest | 0.851 | 0 | 15 | 0.580 |
| Song, D.2019 | Vitamin D intake | highest vs. lowest | 0.736 | 0 | 12 | 0.858 |
| Kazemi, A.2021 | Cheese intake | per 30g /day | 0.219 | 75.285 | 10 | 0.381 |
| Aune, D.2012 | B-carotene | per 5000ug/day | 0.650 | 0 | 10 | 0.592 |
| Liu, F.2022 | Flavonols | highest vs. lowest | 0.372 | 0 | 6 | 0.476 |
| Aune, D.2012 | Fruit intake | per 100 g/day | 0.306 | 39.457 | 10 | 0.732 |
| Ghoreishy, S. M.2023 | Dietary calcium intake | per 350 mg/day | 0.013 | 64.949 | 6 | 0.000 |
| Zeng, J.2020 | Dietary folate intake | highest vs. lowest | 0.132 | 56.156 | 23 | 0.428 |
| Brennan, S. F.2010 | Prudent/healthy dietary pattern | highest vs. lowest | 0.317 | 12.242 | 8 | 0.617 |
| Aune, D.2012 | Fruit intake | highest vs. lowest | 0.377 | 7.76 | 10 | 0.665 |
| Malcomson, F. C.2023 | Adherence score | per 1-point increment | 0.785 | 82.809 | 5 | 0.875 |
| Hu, F.2012 | A-carotene | highest vs. lowest | 0.768 | 0 | 5 | 0.862 |
| Zeng, J.2020 | Vitamin B2 | highest vs. lowest | 0.171 | 42.502 | 10 | 0.709 |
| Aune, D.2012 | Fruits and vegetables intake | highest vs. lowest | 0.585 | 0 | 6 | 0.839 |
| Dong, J. Y.2011 | Total dairy food intake | highest vs. lowest | 0.036 | 41.967 | 10 | 0.194 |
| Ghoreishy, S. M2023. | Dietary calcium intake | highest vs. lowest | 0.033 | 58.844 | 7 | 0.013 |
| Zheng, J. S.2013 | Dietary PUFA intake | highest vs. lowest | 0.034 | 66.699 | 11 | 0.061 |
| Shin, S.2023 | Isoflavone intake | highest vs. lowest | 0.984 | 20.448 | 4 | 0.516 |
| Parra-Soto, S.2022 | Vegetarians | yes vs.no | 0.840 | 29.043 | 4 | 0.038 |
| Ba, D. M.2021 | Higher mushroom consumption | highest vs. lowest | 0.143 | 37.939 | 3 | 0.528 |
| Boyd, N. F.2003 | Total fat intake | highest vs. lowest | 0.042 | 53.954 | 14 | 0.275 |
| Shin, S.2023 | Green tea | highest vs. lowest | 0.283 | 0 | 3 | 0.713 |
| Cao, Y.2016 | Dietary MUFA intake | highest vs. lowest | 0.453 | 62.269 | 17 | 0.031 |
| Cao, Y.2016 | Dietary SFA intake | highest vs. lowest | 0.806 | 52.493 | 20 | 0.189 |
| Mullie, P.2016 | Glycemic load | highest vs. lowest | 0.052 | 65.233 | 12 | 0.836 |
| Li, C.2016 | Dietary cholesterol intake | highest vs. lowest | 0.582 | 5.18 | 6 | 0.809 |
| Kolahdouz M, R.2017 | Ruminant trans-fatty acids | highest vs. lowest | < 3 studies | 8.568 | 2 | NA |
| Chen, H.2021 | DII | highest vs. lowest | 0.510 | 34.067 | 6 | 0.061 |
| Chang, V. C.2019 | Dietary iron intake | highest vs. lowest | 0.543 | 61.376 | 5 | 0.021 |
| Anderson, J. J.2018 | Red meat intake | highest vs. lowest | 0.315 | 51.328 | 10 | 0.127 |
| Florez-Garcia, V. A.2023 | Cadmium | highest vs. lowest | 0.515 | 65.433 | 8 | 0.250 |
| Farvid, M. S.2021 | Total red and processed meat | highest vs. lowest | 0.537 | 33.182 | 12 | 0.266 |
| Cao, Y.2016 | Dietary PUFA intake | highest vs. lowest | 0.122 | 62.28 | 16 | 0.021 |
| Mullie, P.2016 | Glycemic index | highest vs. lowest | 0.086 | 38.94 | 13 | 0.016 |
| Shin, S.2023 | Unhealthy Dietary pattern | highest vs. lowest | 0.923 | 67.104 | 3 | 0.200 |
| Kim, Y.2020 | Dietary PUFA intake | highest vs. lowest | 0.263 | 65.139 | 13 | 0.078 |
| Li, N.2022 | Sugar intake | highest vs. lowest | 0.821 | 59.791 | 10 | 0.646 |
| Nie, X. C.2014 | Black tea consumption | highest vs. lowest | 0.076 | 55.305 | 7 | 0.044 |
| Kazemi, A.2021 | Egg intake | per 50g /day | 0.455 | 48.367 | 11 | 0.204 |
| Nindrea, R. D.2019 | Dietary PUFA intake | highest vs. lowest | 0.913 | 20.138 | 6 | 0.778 |
| Pan, B.2023 | ASBs | per 250ml/day | 0.529 | 57.605 | 3 | 0.803 |
| Hui, C.2013 | Total flavonoids | highest vs. lowest | 0.725 | 0 | 4 | 0.648 |
| Long, T.2022 | Glycemic load | highest vs. lowest | 0.970 | 51.51 | 14 | 0.410 |
| Yu, F.2014 | Tea consumption | per 3 cups | 0.614 | 19.251 | 15 | 0.461 |
| Wang, Y.2020 | Green tea | highest vs. lowest | 0.926 | 0 | 6 | 0.663 |
| Van Puyvelde, H.2023 | Choline | highest vs. lowest | 0.731 | 54.618 | 3 | 0.764 |
| Shin, S.2023 | Soyfoods intake | highest vs. lowest | 0.925 | 52.452 | 3 | 0.716 |
| Ye, X.2023 | Artificial Sweeteners | highest vs. lowest | 0.752 | 66.823 | 3 | 0.255 |
| Zhang, D.2020 | Vitamin C intake | highest vs. lowest | 0.347 | 0 | 14 | 0.198 |
| Brennan, S. F.2010 | Western/unhealthy dietary pattern | highest vs. lowest | 0.989 | 31.365 | 8 | 0.008 |
| Kazemi, A.2021 | Cereals intake | per 20g /day | 0.398 | 0 | 13 | 0.223 |
| Kazemi, A.2021 | Fruit juice intake | per 50g /day | 0.014 | 32.316 | 6 | 0.792 |
| Chan, A. L.2011 | Multivitamin supplement use | highest vs. lowest | 0.872 | 54.664 | 5 | 0.255 |
| Sun, S.2016 | Choline | highest vs. lowest | < 3 studies | 74.082 | 2 | NA |
| Kazemi, A.2021 | Fish intake | per 100g /day | 0.335 | 20.85 | 16 | 0.430 |
| Ren, X.2020 | Folate intake | per 100ug /day | 0.138 | 48.456 | 16 | 0.464 |
| Schlesinger, S.2017 | Sugar intake | per 10g /day | 0.456 | 36.498 | 4 | 0.821 |
| Zeng, J. 2020 | Vitamin B12 | highest vs. lowest | 0.616 | 10.952 | 13 | 0.031 |
| Ghoreishy, S. M. | Total calcium intake | per 350 mg/day | 0.502 | 0 | 3 | 0.694 |
| Kazemi, A.2021 | Milk intake | per 200g /day | 0.346 | 53.318 | 13 | 0.054 |
| Lei, L.2016 | Flavan-3-ols | highest vs. lowest | 0.296 | 14.116 | 4 | 0.686 |
| Li, N.2022 | Dietary carbohydrate intake | highest vs. lowest | 0.736 | 63.457 | 38 | 0.067 |
| Sun, S.2016 | Betaine | highest vs. lowest | < 3 studies | 0 | 2 | NA |
| Reng, Q.2022 | Dietary meat mutagens intake | highest vs. lowest | 0.591 | 22.317 | 3 | 0.688 |
| Zhao, T. T.2019 | Isoflavone intake | highest vs. lowest | 0.195 | 54.273 | 11 | 0.191 |
| Aune, D.2012 | Dietary total carotenoids | per 10000 µg/day | 0.743 | 64.522 | 3 | 0.747 |
| Van Puyvelde, H.2023 | Betaine | highest vs. lowest | 0.538 | 0 | 3 | 0.678 |
| Kazemi, A.2021 | Dairy intake | per 200g /day | 0.993 | 59.036 | 10 | 0.653 |
| Song, D.2019 | Vitamin D intake | per 400IU/d | 0.204 | 0 | 7 | 0.778 |
| Fabiani, R.2016 | Apple intake | highest vs. lowest | 0.138 | 0 | 3 | 0.729 |
| Ghoreishy, S. M.2023 | total calcium intake | highest vs. lowest | 0.776 | 0 | 3 | 0.684 |
| Ren, X.2020 | Folate intake | highest vs. lowest | 0.546 | 54.252 | 19 | 0.152 |
| Touvier, M.2015 | Total cholesterol | per 1 mmol/L | 0.879 | 66.315 | 13 | 0.038 |
| Xiao, Y.2018 | Whole grain intake | highest vs. lowest | 0.929 | 67.103 | 4 | 0.370 |
| Kazemi, A.2021 | Poultry intake | per 100g /day | 0.347 | 27.964 | 12 | 0.251 |
| Lafranconi, A.2018 | Coffee intake | highest vs. lowest | 0.538 | 4.608 | 15 | 0.537 |
| Zhou, Y.2016 | Linoleic acid intake | highest vs. lowest | 0.209 | 49.619 | 8 | 0.066 |
| Xin, Y.2015 | Total vegetable oils | highest vs. lowest | 0.956 | 65.901 | 5 | 0.368 |
| Van Puyvelde, H.2023 | Methionine | highest vs. lowest | 0.645 | 0 | 8 | 0.335 |
| Li, N.2022 | Whole grain intake | highest vs. lowest | 0.279 | 52.646 | 8 | 0.113 |
| Adani, G.2020 | Dietary acrylamide intake | highest vs. lowest | 0.321 | 0 | 10 | 0.823 |
| Aune, D.2012 | A-carotene | per 100ug /day | 0.147 | 44.572 | 6 | 0.252 |
| Parra-Soto, S.2022 | Pescatarians | highest vs. lowest | 0.967 | 36.608 | 5 | 0.787 |
| Han, X.2022 | Dietary vitamin A | highest vs. lowest | 0.371 | 0 | 8 | 0.239 |
| Hu, F.2012 | Total b-carotene | highest vs. lowest | 0.941 | 0 | 9 | 0.816 |
| Liu, F.2022 | Flavones | highest vs. lowest | 0.956 | 66.609 | 5 | 0.098 |
| Buck, K.2010 | Lignans and enterolignans | highest vs. lowest | 0.110 | 59.581 | 3 | 0.315 |
| Chang, V. C.2019 | Total iron intake | highest vs. lowest | < 3 studies | 82.787 | 2 | NA |
| Chen, H. 2018 | Dietary carrot intake | highest vs. lowest | < 3 studies | 53.338 | 2 | NA |
| Yang, J.2023 | Isoflavone intake | highest vs. lowest | 0.216 | 32.829 | 7 | 0.427 |
| Naghshi, Sina.2021 | Total nuts | highest vs. lowest | 0.886 | 0 | 3 | 0.733 |
| Zeng, J.2020 | Vitamin B6 | highest vs. lowest | 0.147 | 19.136 | 13 | 0.276 |
| Liu, X. O.2014 | Vegetable intake | highest vs. lowest | < 3 studies | 0 | 2 | NA |
| Zhao, T. T.2019 | Soyfoods intake | highest vs. lowest | 0.257 | 28.868 | 6 | 0.475 |
| Kazemi, A.2021 | Yogurt intake | per 200g /day | 0.522 | 71.111 | 6 | 0.231 |
| Shin, S.2023 | Healthy Dietary pattern | highest vs. lowest | 0.927 | 0 | 3 | 0.731 |
| Kazemi, A.2021 | Nut intake | per 28g /day | 0.561 | 27.851 | 5 | 0.373 |
| Liu, X.2013 | Cruciferous vegetables intake | highest vs. lowest | < 3 studies | 0 | 2 | NA |
| Markellos, C.2022 | Olive oil intake | highest vs. lowest | 0.534 | 77.967 | 3 | 0.347 |
| ***Imageological diagnosis*** | | | | | | |
| Bodewes, F. T. H.2022 | Breast density | highest vs. lowest | 0.212 | 3.311 | 3 | 0.475 |
| Qu, X.2013 | Bone mineral density | per 0.1 g/m2 | 0.283 | 31.69 | 7 | 0.016 |
| Qu, X.2013 | Bone mineral density | highest vs. lowest | 0.094 | 65.446 | 6 | 0.000 |
| Nagel, G.2017 | Bone mineral density | highest vs. lowest | 0.360 | 61.899 | 7 | 0.000 |
| Chen, J. H.2019 | Bone mineral density | per 0.1 g/m2 | 0.527 | 74.506 | 9 | 0.273 |
| ***Life behaviour risks*** | | | | | | |
| Warren, G. W2013 | Smoking | ever vs. never | 0.908 | 12.816 | 15 | 0.543 |
| Urbano, T.2021 | Light exposure at night | highest vs. lowest | 0.322 | 0 | 7 | 0.128 |
| Chan ,D. S.M.2019 | Physical activity | highest vs. lowest | 0.655 | 0 | 16 | 0.195 |
| Ramalho ,NM.2020 | Education level | highest vs. lowest | 0.827 | 84.586 | 18 | 0.621 |
| Zhou, J.2023 | Famine exposure | ever vs. never | 0.802 | 8.977 | 4 | 0.779 |
| Warren, G.W.2013 | Smoking | ever vs. never | 0.425 | 47.128 | 15 | 0.011 |
| Chan, D.S.M.2019 | Physical activity | highest vs. lowest | 0.042 | 32.158 | 21 | 0.255 |
| Neilson, H.K. | Physical activity | highest vs. lowest | 0.003 | 48.033 | 27 | 0.026 |
| Chen, X.2016 | Physical activity | highest vs. lowest | 0.009 | 27.222 | 38 | 0.001 |
| Chan, D.S.M.2019 | Physical activity | highest vs. lowest | 0.655 | 28.832 | 10 | 0.522 |
| Hidayat, K.2020 | Physical activity | highest vs. lowest | 0.506 | 0 | 4 | 0.437 |
| Armenta-Guirado, B. I.2023 | Lifestyle Quality Indices | highest vs. lowest | 0.097 | 67.781 | 17 | 0.026 |
| Xu, C.2022 | Negative Emotions | ever vs. never | 0.004 | 81.237 | 9 | 0.000 |
| Weinmann, S.2022 | Flight attendants | ever vs. never | 0.577 | 0 | 4 | 0.519 |
| Khuder, S. A.2000 | Passive smoking | ever vs. never | 0.669 | 0 | 3 | 0.552 |
| Lee, J.2021 | Sedentary work | highest vs. lowest | 0.216 | 40.063 | 13 | 0.122 |
| Xiao, W.2022 | Occupational Exposure-organic solvents | ever vs. never | 0.541 | 60.581 | 7 | 0.006 |
| Kim, A. S.2018 | Passing smoking | ever vs. never | 0.827 | 0 | 5 | 0.526 |
| Chong, F.2021 | Sedentary behavior | per 1h/day | 0.512 | 65.488 | 8 | 0.231 |
| Hidayat, K.2020 | Physical activity at a young age | highest vs. lowest | 0.178 | 63.346 | 9 | 0.007 |
| Hiller, T.W.R.2020 | Time in the Sun | highest vs. lowest | 0.069 | 65.178 | 4 | 0.159 |
| Cong, X.2023 | TCDD | ever vs. never | 0.247 | 49.157 | 5 | 0.291 |
| Chen, C.2014 | Passive smoking | ever vs. never | < 3 studies | 83.948 | 2 | 0.526 |
| Lin, Y.2013 | Striking life events | ever vs. never | 0.018 | 66.082 | 3 | 0.000 |
| Chong, F.2021 | Sedentary behavior | highest vs. lowest | 0.213 | 61.968 | 8 | 0.729 |
| Jia, Y.2017 | Depression | ever vs. never | 0.174 | 59.054 | 11 | 0.161 |
| Urbano, T.2021 | Light exposure at night | highest vs. lowest | 0.735 | 0 | 4 | 0.713 |
| Kamdar, B.B.2013 | Long-term night-shift work | highest vs. lowest | 0.696 | 37.434 | 3 | 0.756 |
| Yang, Y.2013 | Passive smoking | ever vs. never | 0.798 | 41.969 | 10 | 0.407 |
| Kamdar, B.B.2013 | Short-term night-shift work | highest vs. lowest | < 3 studies | 0 | 2 | NA |
| Wong, A.T.Y.2021 | Long sleep duration | highest vs. lowest | 0.073 | 10.046 | 15 | 0.797 |
| Wong, A.T.Y.2021 | Short sleep duration | highest vs. lowest | 0.384 | 6.575 | 15 | 0.034 |
| Van, N. T. H.2021 | Night-shift work | highest vs. lowest | 0.335 | 4.879 | 12 | 0.295 |
| Takkouche , B.2005 | Hair dye | ever vs. never | < 3 studies | 73.617 | 2 | NA |
| Shi, Yun.2015 | Physical activity | highest vs. lowest | 0.617 | 32.01 | 3 | 0.720 |
| Heikkilä, K.2013 | Work stress | highest vs. lowest | 0.236 | 0 | 11 | 0.728 |
| Chen, Yuheng.2018 | Long sleep duration | highest vs. lowest | 0.213 | 39.469 | 8 | 0.429 |
| Chen, Yuheng.2018 | Short sleep duration | highest vs. lowest | 0.978 | 20.578 | 8 | 0.103 |
| Chan, D. S. M.2019 | Walking | highest vs. lowest | 0.366 | 0 | 4 | 0.723 |
| ***Environment*** | | | | | | |
| Wei, W.2021 | NO2 | per 10 µg/m3 | 0.080 | 58.292 | 11 | 0.004 |
| Wei, W.2021 | PM10 | per 10 µg/m3 | 0.078 | 74.422 | 6 | 0.135 |
| Wei, W.2021 | PM2.5 | per 10 µg/m3 | 0.518 | 17.395 | 10 | 0.850 |
| Praud, D.2023 | NO2 | per 10 µg/m3 | 0.568 | 31.302 | 7 | 0.209 |
| Hiller, T. W. R.2020 | UVR | highest vs. lowest | 0.944 | 59.788 | 6 | 0.378 |
| ***Past gynaecological history*** | | | | | | |
| Li, C.2021 | Parity | ever vs. never | 0.724 | 0 | 3 | 0.341 |
| Ji, L. W.2019 | Age at first use of oral contraceptives | highest vs. lowest | 0.613 | 74.306 | 10 | 0.195 |
| Deng, Y.2018 | IA | ever vs. never | 0.243 | 80.027 | 24 | 0.013 |
| Guo, J.2015 | Spontaneous abortion | ever vs. never | 0.947 | 23.872 | 12 | 0.459 |
| ***Anthropometric indices*** | | | | | | |
| Neil-Sztramko, S. E.2017 | BMI>=25 | high vs. low, postmenopausal | 0.291 | 0 | 18 | 0.392 |
| Hidayat, K.2018 | BMI iya | per 5 kg/m2 | 0.000 | 81.253 | 24 | 0.000 |
| Byun, D.2022 | BMI | per 5 kg/m2 | 0.120 | 67.392 | 21 | 0.008 |
| Namazi, N.2019 | Fat mass | highest vs. lowest | 0.894 | 68.895 | 7 | 0.021 |
| Hao, Y.2021 | Weight gain | highest vs. lowest | 0.558 | 62.317 | 16 | 0.666 |
| Chen, Y.2017 | BMI | highest vs. lowest | 0.226 | 78.896 | 28 | 0.199 |
| Neil-Sztramko, S. E.2017 | BMI <25 | high vs. low, postmenopausal | 0.011 | 39.226 | 18 | 0.047 |
| Xue, F.2007 | Birth length | highest vs. lowest | 0.862 | 0 | 3 | 0.701 |
| Dehesh, T.2023 | BMI >=30 | highest vs. lowest | 0.741 | 84.765 | 12 | 0.017 |
| Xu, X.2009 | Birth weight | highest vs. lowest | 0.022 | 0 | 4 | 0.456 |
| Liu, K.2018 | BMI | per 5 kg/m2 | 0.610 | 75.531 | 12 | 0.504 |
| Chan, D. S. M.2019 | Weight loss | highest vs. lowest | 0.289 | 0 | 16 | 0.432 |
| Neil-Sztramko, S. E.2017 | BMI >=30 | highest vs. lowest | 0.971 | 0 | 4 | 0.694 |
| Zhou, W.2020 | Birth weight | highest vs. lowest | 0.058 | 43.803 | 8 | 0.166 |
| Xu, X.2009 | Birth weight | per 1 kg | 0.981 | 37.232 | 5 | 0.431 |
| Amadou, A.2013 | Waist-to-hip ratio | per 0.1 | 0.077 | 0 | 3 | 0.671 |
| Amadou, A.2013 | Height | per 10 cm | 0.161 | 0 | 6 | 0.798 |
| ***Biomarkers*** | | | | | | |
| Key, T. J.2010 | IGF1 concentrations | highest vs. lowest | 0.616 | 0 | 16 | 0.695 |
| Amerizadeh, A.2022 | Serum TG levels | highest vs. lowest | 0.314 | 0 | 11 | 0.530 |
| Nouri, M.2022 | Serum TG levels | highest vs. lowest | 0.194 | 0 | 9 | 0.466 |
| Ren, X.2020 | Plasma folate | highest vs. lowest | < 3 studies | 67.881 | 2 | NA |
| Renehan, A. G.2004 | IGF1 concentrations | highest vs. lowest | 0.562 | 0 | 4 | 0.778 |
| Renehan, A. G.2004 | IGFBP-3 | highest vs. lowest | 0.614 | 0 | 3 | 0.700 |
| Chang, V. C.2019 | Serum/plasma iron | highest vs. lowest | 0.352 | 69.071 | 3 | 0.551 |
| Song, D.2019 | Blood vitamin D | highest vs. lowest | 0.643 | 30.719 | 6 | 0.109 |
| Lou, M. W. C.2023 | CRP | highest vs. lowest | 0.055 | 61.041 | 9 | 0.379 |
| Wang, M.2016 | Plasma prolactin levels | highest vs. lowest | 0.685 | 0 | 6 | 0.569 |
| Walker, K.2011 | Circulating E2 levels | highest vs. lowest | 0.571 | 19.681 | 7 | 0.351 |
| Ren, X.2020 | Plasma folate | per 5ng/ml | < 3 studies | 70.269 | 2 | NA |
| Song, D.20119 | Blood vitamin D | per 5 nmol/L | 0.695 | 85.14 | 4 | 0.018 |
| Nouri, M.2022 | HDL–C | highest vs. lowest | 0.696 | 0 | 8 | 0.677 |
| Ma, H. Q.2016 | Serum triglycerides | highest vs. lowest | 0.199 | 45.588 | 8 | 0.187 |
| Nouri, M.2022 | Serum TC levels | highest vs. lowest | 0.491 | 69.562 | 13 | 0.364 |
| Xu, J.2017 | Urinary 6-sulfatoxymelatonin levels | highest vs. lowest | 0.806 | 34.091 | 6 | 0.762 |
| Amerizadeh, A.2022 | HDL–C | highest vs. lowest | 0.926 | 35.594 | 8 | 0.409 |
| Amerizadeh, A.2022 | Serum TC levels | highest vs. lowest | 0.954 | 74.293 | 14 | 0.424 |
| Nouri, M.2022 | Serum apolipoprotein A levels | highest vs. lowest | 0.165 | 83.531 | 3 | 0.377 |
| Amerizadeh, A2022. | LDL–C | highest vs. lowest | 0.760 | 0 | 6 | 0.802 |
| Wong, A. T. Y.2021 | Urinary aMT6s | highest vs. lowest | 0.418 | 20.527 | 7 | 0.560 |
| Nouri, M.2022 | HDL–C | per 1 mmol/L | 0.035 | 58.27 | 5 | 0.378 |
| Han, X.2022 | Circulating high levels of vitamin A | highest vs. lowest | 0.569 | 29.845 | 3 | 0.817 |
| ***Use of medical/hormonal therapy*** | | | | | | |
| Karasneh, R. A.2017 | Cardiac glycosides use | ever vs. never | 0.324 | 0 | 6 | 0.838 |
| Thakur, A. A.20118 | Calcium channel blockers | ever vs. never | 0.186 | 0 | 3 | 0.628 |
| Simin, J.2020 | Antibiotic use | ever vs. never | < 3 studies | 0 | 2 | NA |
| Li, Y. Y.2020 | Bisphosphonates | ever vs. never | 0.855 | 15.09 | 7 | 0.629 |
| Leung, J. C. N.2022 | Antipsychotic use | ever vs. never | 0.265 | 74.571 | 4 | 0.007 |
| Gao, Z.2022 | Antipsychotic use | ever vs. never | 0.941 | 81.655 | 4 | 0.355 |
| Zhuang, Y.2022 | Antidepressant use | ever vs. never | 0.433 | 66.214 | 7 | 0.543 |
| Bakierzynska, M.2023 | Aspirin intake | ever vs. never | 0.076 | 82.013 | 28 | 0.014 |
| Peng, R.2020 | Bisphosphonates | ever vs. never | 0.464 | 44.92 | 7 | 0.208 |
| Chen, Y.2023 | Thiazolidinediones | ever vs. never | 0.244 | 0 | 5 | 0.561 |
| Chen, Y.2023 | Insulins | ever vs. never | 0.043 | 74.517 | 11 | 0.575 |
| Yang, J,2023 | Beta blockers | ever vs. never | 0.635 | 93.051 | 9 | 0.888 |
| Ni, X. J.2012 | Postmenopausal hormone therapy | ever vs. never | < 3 studies | 74.182 | 2 | NA |
| Ni, H.2017 | Diuretic use | ever vs. never | 0.854 | 71.989 | 6 | 0.826 |
| Chen, Y.2023 | Insulin secretagogues | ever vs. never | 0.850 | 7.95 | 4 | 0.684 |
| Undela, K.2012 | Statin use | ever vs. never | 0.447 | 9.575 | 13 | 0.285 |
| Du, X.2012 | Insulin glargine | ever vs. never | 0.077 | 0 | 5 | 0.642 |
| Santucci, C.2021 | Aspirin intake | ever vs. never | 0.389 | 72.117 | 20 | 0.001 |
| Qiao, Y.2018 | Aspirin intake | ever vs. never | 0.761 | 69.731 | 20 | 0.005 |
| Tang, G. H.2018 | Metformin | ever vs. never | 0.645 | 0 | 11 | 0.003 |
| Du, R.2018 | Thiazolidinedione | ever vs. never | 0.203 | 75.432 | 7 | 0.277 |
| Ni, H.2017 | ACEI/ARB | ever vs. never | 0.436 | 6.587 | 9 | 0.077 |
| Indrakusuma, Aabp.2022 | Antipsychotic use | ever vs. never | < 3 studies | 92.707 | 2 | NA |
| Chen, Y. | Biguanides | ever vs. never | 0.252 | 99.072 | 6 | 0.717 |
| ***Pre-existing medical conditions and interventions*** | | | | | | |
| Yao, X.2023 | Atrial fibrillation | ever vs. never | 0.656 | 0 | 5 | 0.545 |
| Hardefeldt, P. J.2012 | Antibody | ever vs. never | 0.025 | 65.786 | 8 | 0.000 |
| Wu, D.2023 | Obstructive sleep apnea | ever vs. never | 0.655 | 99.46 | 9 | 0.005 |
| Shi, T.2018 | Periodontal disease | ever vs. never | 0.079 | 21.202 | 7 | 0.004 |
| Guo, M.2019 | Metabolic syndrome | ever vs. never | 0.002 | 76.861 | 17 | 0.000 |
| Tran, T. V.2023 | Hyperthyroidism | ever vs. never | 0.399 | 28.832 | 12 | 0.348 |
| Wilson, R. B.2023 | Bariatric surgery | ever vs. never | 0.946 | 96.511 | 13 | 0.622 |
| Hardefeldt, P. J.2012 | Autoimmune thyroiditis | ever vs. never | 0.148 | 0 | 4 | 0.017 |
| Hardefeldt, P. J.2012 | Goitre | ever vs. never | 0.879 | 0 | 4 | 0.163 |
| Wei, L.2021 | Sleep-disordered breathing | ever vs. never | 0.036 | 94.576 | 8 | 0.000 |
| Shao, J.2018 | Periodontal disease | ever vs. never | 0.093 | 51.455 | 11 | 0.082 |
| Xiong, F.2023 | Diabetes | ever vs. never | 0.092 | 98.515 | 46 | 0.977 |
| Chen, S.2021 | Hypothyroidism | ever vs. never | 0.137 | 0 | 5 | 0.811 |
| Chen, Hsin-Hao.2023 | CAD | ever vs. never | 0.105 | 66.937 | 5 | 0.033 |
| Ishihara, B. P.2020 | Bariatric surgery | ever vs. never | 0.940 | 92.033 | 6 | 0.784 |
| Yap, D. W. T.2022 | Obstructive sleep apnea | ever vs. never | 0.074 | 96.847 | 5 | 0.000 |
| Chen, S.2021 | Hyperthyroidism | ever vs. never | < 3 studies | 70.276 | 2 | NA |
| Zheng, X.2022 | Heart failure | ever vs. never | 0.680 | 95.411 | 5 | 0.605 |
| Vojtechova, P.2009 | Atopy | ever vs. never | 0.136 | 43.585 | 4 | 0.248 |
| Wang, T.2015 | Parkinson’s disease | ever vs. never | 0.465 | 73.094 | 7 | 0.000 |
| Wang, Y.2020 | GDM | ever vs. never | 0.702 | 88.631 | 9 | 0.001 |
| Bonifazi, M.2013 | Systemic sclerosis | ever vs. never | 0.802 | 0 | 7 | 0.698 |
| Seretis, A.2019 | Hypertension | ever vs. never | 0.065 | 58.995 | 11 | 0.948 |
| Vojtechova, P.2009 | Hay fever | ever vs. never | 0.238 | 7.319 | 6 | 0.725 |
| Ye, J.2022 | Endometriosis | ever vs. never | 0.311 | 51.807 | 11 | 0.295 |
| Vojtechova, P.2009 | Allergy | ever vs. never | 0.067 | 0 | 7 | 0.678 |
| Peng, C.2023 | Migraine | ever vs. never | 0.661 | 66.996 | 4 | 0.362 |
| Najdi, N.2022 | Tubal ligation | ever vs. never | 0.867 | 20.428 | 6 | 0.802 |
| Tran, T. V.2023 | Hypothyroidism | ever vs. never | 0.364 | 59.079 | 10 | 0.237 |
| Hassan, H.2023 | Hysterectomy with BSO | ever vs. never | 0.996 | 85.922 | 8 | 0.001 |
| Sun, M.2018 | PE | ever vs. never | 0.436 | 61.167 | 8 | 0.229 |
| Vojtechova, P.2009 | Asthma | ever vs. never | 0.416 | 91.325 | 10 | 0.958 |
| Li, Z.2022 | Polycystic ovary syndrome | ever vs. never | 0.056 | 0 | 4 | 0.685 |
| Sergentanis, T. N.2014 | IVF | ever vs. never | 0.905 | 51.092 | 6 | 0.129 |
| Heting, M.2023 | Levonorgestrel intrauterine system | ever vs. never | 0.864 | 92.916 | 3 | 0.793 |
| ***Congenital factor*** | | | | | | |
| Xue, F.2007 | Paternal age | per 15 years | 0.331 | 0 | 3 | 0.345 |
| Xue, F.2007 | Twin membership  (including monozygotic and dizygotic) | yes vs. no | < 3 studies | 0 | 2 | NA |

**Abbreviation：**ACEI/ARB : Angiotensin-converting enzyme inhibitor / angiotensin-receptor；BMI, body mass index; BMI iya, body mass index in young adulthood; CI, confidence interval; HRT, hormone replacement therapy; PA, physical activity; PoMP, postmenopausal; ELF-EMFs: extremely low-frequency electromagnetic fields；DII, dietary inflammatory index; SSBs, sugar-sweetened beverages; SFA, saturated fatty acids ;MUFA, monounsaturated fatty acids; PUFA, polyunsaturated fatty acids; ASBs, artificially sweetened beverages ;UVR, ultraviolet radiation; IA, Induced abortion;IGF-1,Insulin-like growth factor 1 ;IGFBP-3,IGF binding protein 3TG,total triglycerides; TC, total cholesterol；CRP,C-reactive protein; CAD, coronary artery disease; GDM, Gestational diabetes mellitus ;PE, Preeclampsia ;BSO, bilateral salpingo-oophorectom; NA, not available;

**Key:** All statistical tests were two-sided

**Supplemental Table 5:** A Measurement Tool to Assess Systematic Reviews (AMSTAR-2) Summary quality assessment for all included systematic review

| **Questions**  **Author, year** | **1** | **2** | **3** | **4** | **5** | **6** | **7** | **8** | **9** | **10** | **11** | **12** | **13** | **14** | **15** | **16** | **Score** |
| --- | --- | --- | --- | --- | --- | --- | --- | --- | --- | --- | --- | --- | --- | --- | --- | --- | --- |
| Zhou, J.2023 | Yes | Yes | Yes | Yes | Yes | Yes | Yes | Yes | Yes | No | Yes | Yes | Yes | Yes | Yes | Yes | **High** |
| Ye, X.2023 | Yes | Yes | Yes | Yes | No | Yes | Yes | Yes | No | No | Yes | No | Yes | Yes | Yes | Yes | **Low** |
| Yao, X.2023 | Yes | Yes | Yes | Yes | Yes | Yes | Yes | Yes | Yes | Yes | Yes | Yes | Yes | Yes | Yes | Yes | **High** |
| Yang, J.2023 | Yes | Yes | Yes | Yes | Yes | Yes | Yes | Yes | Yes | Yes | Yes | Yes | No | Yes | Yes | Yes | **Low** |
| Yang, J.2023 | Yes | Yes | Yes | Yes | Yes | Yes | Yes | Yes | Yes | Yes | Yes | Yes | Yes | Yes | Yes | Yes | **High** |
| Xiong, F. 2023 | Yes | Yes | Yes | No | Yes | Yes | Yes | Yes | Yes | Yes | Yes | Yes | No | Yes | Yes | No | **Critically low** |
| Wilson, R. B.2023 | Yes | Yes | Yes | Yes | Yes | Yes | Yes | Yes | Partial yes | No | Yes | Yes | Yes | Yes | Yes | Yes | **High** |
| Wang, Y. T.2023 | Yes | Yes | Yes | Yes | Yes | Yes | Yes | Yes | Yes | No | Yes | Yes | Yes | Yes | Yes | Yes | **High** |
| Van Puyvelde, H.2023 | Yes | Yes | Yes | Yes | No | No | Yes | Yes | Yes | No | Yes | Yes | Yes | Yes | Yes | Yes | **Moderate** |
| Shin, S.2023 | Yes | Yes | Yes | Yes | Yes | Yes | Yes | Yes | Yes | No | Yes | Yes | No | Yes | Yes | Yes | **Low** |
| Armenta-G, B. I.2023 | Yes | Yes | Yes | Yes | Yes | Yes | No | Yes | Yes | Yes | Yes | Yes | No | Yes | Yes | Yes | **Critically low** |
| Praud, D.2023 | Yes | Yes | Yes | Yes | Yes | Yes | Yes | Yes | Yes | Yes | Yes | Yes | No | Yes | Yes | Yes | **Low** |
| Peng, C.2023 | Yes | Yes | Yes | Yes | Yes | Yes | Yes | Yes | No | Yes | Yes | No | Yes | Yes | Yes | Yes | **Low** |
| Pan, B.2023 | Yes | Yes | Yes | Yes | Yes | Yes | Yes | Yes | Partial yes | No | Yes | Yes | No | Yes | Yes | Yes | **Low** |
| Tran, T. V.2023 | Yes | Yes | Yes | Yes | No | No | Yes | Yes | Yes | Yes | Yes | Yes | No | Yes | Yes | Yes | **Low** |
| Malcomson, F. C.2023 | Yes | Yes | Yes | Yes | Yes | Yes | Yes | Yes | Yes | Yes | Yes | Yes | No | Yes | Yes | Yes | **Low** |
| Lou, M. W. C.2023 | Yes | Yes | Yes | Yes | Yes | Yes | Yes | Yes | Yes | Yes | Yes | Yes | No | Yes | No | Yes | **Critically low** |
| Lian, Y.2023 | Yes | No | Yes | Yes | No | Yes | Yes | Yes | Yes | Yes | Yes | Yes | Yes | Yes | Yes | Yes | **Low** |
| Heting, M.2023 | Yes | No | Yes | Yes | Yes | Yes | Yes | Yes | No | No | Yes | No | No | Yes | No | Yes | **Critically low** |
| Hassan, H.2023 | Yes | Yes | Yes | Yes | Yes | Yes | Yes | Yes | Yes | Yes | Yes | Yes | No | Yes | No | Yes | **Critically low** |
| Ghoreishy, S. M2023 | Yes | No | Yes | Yes | No | Yes | Yes | Yes | No | Yes | Yes | No | Yes | Yes | Yes | Yes | **Critically low** |
| Florez-G, V.A.2023 | Yes | Yes | Yes | Yes | Yes | Yes | Yes | Yes | Partial yes | Yes | Yes | Yes | No | Yes | Yes | Yes | **Low** |
| Fitzpatrick, D.2023 | Yes | No | Yes | Yes | Yes | Yes | Yes | No | Yes | Yes | Yes | Yes | No | Yes | Yes | Yes | **Critically low** |
| Dehesh, T.2023 | Yes | Yes | Yes | Yes | Yes | Yes | Yes | Yes | No | No | Yes | No | No | Yes | Yes | Yes | **Critically low** |
| de Oliveira, V.A.2023 | Yes | No | Yes | Yes | Yes | Yes | Yes | Yes | No | No | Yes | No | No | Yes | Yes | Yes | **Critically low** |
| Cong, X.2023 | Yes | No | Yes | Yes | Yes | Yes | Yes | Yes | Yes | Yes | Yes | Yes | Yes | Yes | Yes | Yes | **Low** |
| Chen, Y.2023 | Yes | Yes | Yes | Yes | Yes | Yes | Yes | Yes | Yes | No | Yes | Yes | No | Yes | No | Yes | **Critically low** |
| Chen, Hsin-Hao2023 | Yes | Yes | Yes | Yes | Yes | Yes | Yes | Yes | Yes | No | Yes | Yes | No | Yes | Yes | Yes | **Low** |
| Bakierzynska, M.2023 | Yes | Yes | Yes | Yes | No | Yes | Yes | Yes | Partial yes | No | Yes | Yes | No | Yes | Yes | Yes | **Low** |
| Li, Z.2022 | Yes | No | Yes | Yes | No | No | Yes | Yes | Yes | Yes | Yes | Yes | Yes | Yes | Yes | Yes | **Low** |
| Zhuang, Y.2022 | Yes | Yes | Yes | Yes | No | Yes | Yes | Yes | Yes | Yes | Yes | Yes | Yes | Yes | Yes | Yes | **Moderate** |
| Zheng, X.2022 | Yes | No | Yes | Yes | Yes | Yes | Yes | Yes | Yes | Yes | Yes | Yes | No | Yes | No | Yes | **Critically low** |
| Zhang, J.2022 | Yes | No | Yes | Yes | Yes | Yes | Yes | Yes | Yes | No | Yes | Yes | Yes | Yes | Yes | Yes | **Low** |
| Ye, J.2022 | Yes | No | Yes | Yes | No | Yes | Yes | Yes | Yes | No | Yes | Yes | Yes | Yes | Yes | Yes | **Low** |
| Yap, D. W. T.2022 | Yes | Yes | Yes | Yes | Yes | Yes | Yes | Yes | Partial yes | Yes | Yes | Yes | No | Yes | Yes | Yes | **Low** |
| Xu, C.2022 | Yes | Yes | Yes | Yes | Yes | Yes | Yes | Yes | Yes | No | Yes | Yes | Yes | Yes | Yes | Yes | **Moderate** |
| Xiao, W.2022 | Yes | No | Yes | Yes | Yes | Yes | Yes | Yes | Yes | No | Yes | Yes | Yes | Yes | Yes | Yes | **Low** |
| van Weers, S.2022 | Yes | No | Yes | Yes | No | No | Yes | Yes | Partial yes | No | Yes | Yes | No | Yes | Yes | Yes | **Critically low** |
| Reng, Q.2022 | Yes | No | Yes | Yes | Yes | Yes | Yes | Yes | No | No | Yes | No | Yes | Yes | Yes | Yes | **Critically low** |
| Weinmann, S.2022 | Yes | No | Yes | No | Yes | Yes | Yes | Yes | Yes | No | Yes | Yes | No | No | No | Yes | **Critically low** |
| Parra-Soto, S.2022 | Yes | Yes | Yes | Yes | Yes | Yes | Yes | Yes | Yes | Yes | Yes | Yes | No | Yes | No | Yes | **Critically low** |
| Nouri, M.2022 | Yes | Yes | Yes | Yes | Yes | Yes | Yes | Yes | Partial yes | No | Yes | Yes | No | Yes | Yes | Yes | **Low** |
| Najdi, N.2022 | Yes | No | Yes | Yes | Yes | Yes | Yes | Yes | Yes | Yes | Yes | Yes | No | Yes | Yes | Yes | **Critically low** |
| Manouchehri, E.2022 | Yes | No | Yes | Yes | Yes | Yes | Yes | Yes | Partial yes | Yes | Yes | Yes | No | Yes | Yes | Yes | **Critically low** |
| Markellos, C.2022 | Yes | No | Yes | Yes | Yes | Yes | Yes | Yes | Yes | No | Yes | Yes | No | Yes | Yes | Yes | **Critically low** |
| Long, T.2022 | Yes | Yes | Yes | Yes | No | Yes | Yes | Yes | Yes | Yes | Yes | Yes | No | Yes | Yes | Yes | **Low** |
| Liu, H.2022 | Yes | No | Yes | Yes | No | No | Yes | Yes | Yes | Yes | Yes | Yes | No | Yes | Yes | Yes | **Critically low** |
| Liu, F.2022 | Yes | Yes | Yes | Yes | Yes | Yes | Yes | Yes | Partial yes | Yes | Yes | Yes | Yes | Yes | Yes | Yes | **High** |
| Li, N.2022 | Yes | Yes | Yes | Yes | Yes | Yes | Yes | Yes | Yes | No | Yes | Yes | Yes | Yes | Yes | Yes | **Moderate** |
| Khoramdad, M.2022 | Yes | No | No | Yes | Yes | Yes | Yes | Yes | Yes | Yes | Yes | Yes | Yes | Yes | Yes | Yes | **Low** |
| Kacimi, S. E. O.2022 | Yes | No | Yes | Yes | Yes | Yes | Yes | Yes | Partial yes | No | Yes | Yes | Yes | Yes | Yes | Yes | **Low** |
| Islam, M. A.2022 | Yes | No | Yes | Yes | No | No | Yes | Yes | Yes | No | Yes | Yes | Yes | Yes | Yes | Yes | **Low** |
| Han, X.2022 | Yes | No | Yes | Yes | Yes | Yes | Yes | Yes | Yes | No | Yes | Yes | Yes | Yes | Yes | Yes | **Low** |
| Han, M.2022 | Yes | No | Yes | Yes | Yes | Yes | Yes | Yes | Yes | Yes | Yes | Yes | No | Yes | Yes | Yes | **Critically low** |
| Gao, Z.2022 | Yes | Yes | Yes | Yes | Yes | Yes | Yes | Yes | Yes | No | Yes | Yes | Yes | Yes | Yes | Yes | **Moderate** |
| Bodewes, F. T. H.2022 | Yes | Yes | Yes | Yes | Yes | Yes | Yes | Yes | Partial yes | No | Yes | Yes | No | Yes | Yes | Yes | **Low** |
| Gamboa-Loira, B.2022 | Yes | No | Yes | Yes | No | No | Yes | Yes | Yes | Yes | Yes | Yes | No | Yes | Yes | Yes | **Critically low** |
| Byun, D.2022 | Yes | No | Yes | Yes | Yes | Yes | Yes | No | Yes | No | Yes | Yes | No | Yes | Yes | Yes | **Critically low** |
| Amerizadeh, A.2022 | Yes | No | Yes | Yes | Yes | Yes | Yes | Yes | Yes | Yes | Yes | Yes | Yes | Yes | Yes | Yes | **Low** |
| Zhang, H.2021 | Yes | No | Yes | Partial yes | No | No | Yes | Yes | No | No | Yes | No | No | Yes | Yes | Yes | **Critically low** |
| Wong, A. T. Y.2021 | Yes | No | Yes | Yes | Yes | Yes | Yes | No | Partial yes | No | Yes | Yes | No | Yes | No | Yes | **Critically low** |
| Wei, W.2021 | Yes | No | Yes | Yes | Yes | Yes | Yes | Yes | Partial yes | No | Yes | Yes | No | Yes | Yes | Yes | **Critically low** |
| Wei, L.2021 | Yes | No | Yes | Yes | No | Yes | Yes | Yes | Yes | No | Yes | Yes | Yes | Yes | Yes | Yes | **Low** |
| Urbano, T.2021 | Yes | No | Yes | Yes | Yes | Yes | Yes | Yes | Yes | Yes | Yes | Yes | Yes | Yes | Yes | Yes | **Low** |
| Wong, A. T. Y.2021 | Yes | No | Yes | Yes | Yes | No | Yes | Yes | Yes | Yes | Yes | Yes | No | Yes | No | Yes | **Critically low** |
| Van, N. T. H.2021 | Yes | Yes | Yes | Yes | Yes | Yes | Yes | Yes | Partial yes | No | Yes | Yes | No | Yes | Yes | Yes | **Low** |
| Nourmo, H.2021 | Yes | No | Yes | Yes | Yes | Yes | Yes | Yes | No | No | Yes | No | No | Yes | Yes | Yes | **Critically low** |
| Michels, N.2021 | Yes | No | Yes | Yes | Yes | No | Yes | Yes | Yes | No | Yes | Yes | No | Yes | Yes | Yes | **Critically low** |
| Naghshi, Sina2021 | Yes | No | Yes | Yes | No | Yes | Yes | Yes | Yes | Yes | Yes | Yes | Yes | Yes | Yes | Yes | **Low** |
| Li, C.2021 | Yes | No | Yes | Yes | Yes | Yes | Yes | Yes | Yes | Yes | Yes | Yes | Yes | Yes | Yes | Yes | **Low** |
| Lee, J.2021 | Yes | Yes | Yes | Yes | Yes | No | Yes | Yes | Yes | No | Yes | Yes | No | Yes | Yes | Yes | **Low** |
| Keefe, David.2021 | Yes | Yes | Yes | Yes | Yes | Yes | Yes | Yes | No | No | Yes | No | No | Yes | Yes | Yes | **Critically low** |
| Kazemi, A.2021 | Yes | Yes | Yes | Yes | No | Yes | Yes | Yes | Partial yes | Yes | Yes | Yes | No | Yes | Yes | Yes | **Low** |
| Hao, Y.2021 | Yes | No | Yes | Yes | Yes | No | Yes | Yes | No | Yes | Yes | No | Yes | Yes | Yes | Yes | **Critically low** |
| Farvid, M. S.2021 | Yes | No | Yes | Yes | No | No | Yes | Yes | No | No | Yes | No | No | Yes | Yes | Yes | **Critically low** |
| Chong, F.2021 | Yes | No | Yes | Yes | No | Yes | Yes | Yes | Yes | Yes | Yes | Yes | Yes | Yes | Yes | Yes | **Low** |
| Chen, H.2021 | Yes | No | Yes | Yes | Yes | Yes | Yes | Yes | Yes | No | Yes | Yes | No | Yes | Yes | Yes | **Critically low** |
| Barańska, A.2021 | Yes | No | Yes | Yes | Yes | Yes | Yes | Yes | Yes | No | Yes | Yes | No | Yes | Yes | Yes | **Critically low** |
| Ba, D. M.2021 | Yes | No | Yes | Yes | Yes | Yes | Yes | Yes | Yes | No | Yes | Yes | Yes | Yes | Yes | Yes | **Low** |
| Arafat, H. M.2021 | Yes | Yes | Yes | Yes | Yes | Yes | Yes | Yes | Yes | No | Yes | Yes | Yes | Yes | No | Yes | **Low** |
| Zhou, W.2020 | Yes | No | Yes | Yes | Yes | Yes | Yes | Yes | Yes | No | Yes | Yes | Yes | Yes | Yes | Yes | **Low** |
| Zhang, D.2020 | Yes | No | Yes | Yes | Yes | Yes | Yes | Yes | No | No | Yes | No | No | Yes | Yes | Yes | **Critically low** |
| Zeng, J.2020 | Yes | No | Yes | Yes | Yes | Yes | Yes | Yes | No | No | Yes | No | Yes | Yes | Yes | Yes | **Critically low** |
| Wu, Y.2020 | Yes | No | Yes | Yes | Yes | No | Yes | Yes | No | No | Yes | No | Yes | Yes | Yes | Yes | **Critically low** |
| Wei, Y.2020 | Yes | No | Yes | Yes | No | No | Yes | Yes | No | Yes | Yes | No | Yes | Yes | Yes | Yes | **Critically low** |
| Wang, Y.2020 | Yes | No | Yes | Yes | Yes | No | Yes | Yes | Yes | Yes | Yes | Yes | Yes | Yes | Yes | Yes | **Low** |
| Wang, Y.2020 | Yes | No | Yes | Yes | Yes | Yes | Yes | Yes | Partial yes | Yes | Yes | Yes | Yes | Yes | Yes | Yes | **Low** |
| Wang, Q.2020 | Yes | No | Yes | Yes | Yes | Yes | Yes | Yes | Yes | No | Yes | Yes | Yes | Yes | Yes | Yes | **Low** |
| Song, H. J.2020 | Yes | Yes | Yes | Yes | Yes | Yes | Yes | Yes | Yes | Yes | Yes | Yes | No | Yes | Yes | Yes | **Low** |
| Simin, J.2020 | Yes | No | Yes | Yes | Yes | No | Yes | Yes | Yes | No | Yes | Yes | No | Yes | Yes | Yes | **Critically low** |
| Ren, X.2020 | Yes | No | Yes | Yes | Yes | No | Yes | Yes | Yes | No | Yes | Yes | No | Yes | Yes | Yes | **Critically low** |
| Ramalho, N.M.2020 | Yes | No | Yes | Yes | Yes | Yes | Yes | Yes | Yes | Yes | Yes | Yes | Yes | Yes | Yes | Yes | **Low** |
| Peng, R.2020 | Yes | Yes | Yes | Yes | Yes | Yes | Yes | Yes | Yes | No | Yes | Yes | No | Yes | Yes | Yes | **Low** |
| Okekunle, A. P.2020 | Yes | Yes | Yes | Yes | Yes | Yes | Yes | Yes | Yes | Yes | Yes | Yes | Yes | Yes | Yes | Yes | **Moderate** |
| Li, M.2020 | Yes | Yes | Yes | Yes | Yes | Yes | Yes | Yes | Yes | No | Yes | Yes | No | Yes | Yes | Yes | **Low** |
| Kim, Y.2020 | Yes | No | Yes | Yes | Yes | Yes | Yes | Yes | Yes | Yes | Yes | Yes | Yes | Yes | Yes | Yes | **Low** |
| Khatami, A.2020 | Yes | No | Yes | Yes | Yes | No | Yes | Yes | Yes | No | Yes | Yes | Yes | Yes | Yes | Yes | **Low** |
| Jin, Q.2020 | Yes | No | Yes | Yes | Yes | Yes | Yes | Yes | Yes | No | Yes | Yes | Yes | Yes | Yes | Yes | **Low** |
| Hiller, T. W. R.2020 | Yes | No | Yes | Yes | Yes | Yes | Yes | Yes | Yes | No | Yes | Yes | Yes | Yes | Yes | Yes | **Low** |
| Hidayat, K.2020 | Yes | No | Yes | Yes | Yes | Yes | Yes | Yes | Yes | Yes | Yes | Yes | Yes | Yes | Yes | Yes | **Low** |
| Adani, G.2020 | Yes | No | Yes | Yes | No | No | Yes | Yes | No | Yes | Yes | No | Yes | Yes | Yes | Yes | **Critically low** |
| Zhao, T. T.2019 | Yes | No | Yes | Yes | Yes | Yes | Yes | Yes | Yes | Yes | Yes | Yes | No | Yes | Yes | Yes | **Critically low** |
| Zhang, L.2019 | Yes | No | Yes | Yes | No | No | Yes | Yes | Yes | Yes | Yes | Yes | Yes | Yes | Yes | Yes | **Low** |
| Yu, S.2019 | Yes | No | Yes | Yes | Yes | Yes | Yes | Yes | Yes | No | Yes | Yes | Yes | Yes | Yes | Yes | **Low** |
| Xiping, Z.2019 | Yes | No | Yes | Yes | No | No | Yes | Yes | No | Yes | Yes | No | No | Yes | Yes | Yes | **Critically low** |
| Vishwakarma, G.2019 | Yes | No | Yes | No | No | No | Yes | Yes | No | No | Yes | No | No | Yes | Yes | Yes | **Critically low** |
| Yoon, Y. S.2019 | Yes | No | Yes | Yes | Yes | Yes | Yes | Yes | Yes | Yes | Yes | Yes | Yes | Yes | Yes | Yes | **Low** |
| Song, D.2019 | Yes | No | Yes | Yes | Yes | Yes | Yes | Yes | Yes | Yes | Yes | Yes | Yes | Yes | Yes | Yes | **Low** |
| Seretis, A.2019 | Yes | No | Yes | Yes | Yes | Yes | Yes | Yes | Yes | No | Yes | Yes | No | Yes | Yes | No | **Critically low** |
| Ren, C.2019 | Yes | Yes | Yes | Yes | Yes | Yes | Yes | Yes | No | No | Yes | No | No | Yes | Yes | Yes | **Critically low** |
| Nindrea, R. D.2019 | Yes | No | Yes | Yes | No | Yes | Yes | Yes | Yes | No | Yes | Yes | No | No | Yes | Yes | **Critically low** |
| Nindrea, R. D.2019 | Yes | No | Yes | Yes | Yes | Yes | Yes | Yes | Yes | No | Yes | Yes | No | Yes | Yes | Yes | **Critically low** |
| Nindrea, R. D.2019 | Yes | No | Yes | Yes | No | No | Yes | Yes | Yes | No | Yes | Yes | No | Yes | Yes | Yes | **Critically low** |
| Mao, Jie.2019 | Yes | No | Yes | Yes | No | No | Yes | Yes | Yes | Yes | Yes | Yes | No | Yes | Yes | Yes | **Critically low** |
| Namazi, N.2019 | Yes | Yes | Yes | Yes | No | Yes | Yes | Yes | Partial yes | No | Yes | Yes | Yes | Yes | Yes | Yes | **Moderate** |
| Ji, L. W.2019 | Yes | No | Yes | Yes | Yes | Yes | Yes | Yes | Yes | No | Yes | Yes | Yes | Yes | Yes | Yes | **Low** |
| Hossain, S.2019 | Yes | No | Yes | Yes | Yes | Yes | Yes | Yes | Partial yes | Yes | Yes | Yes | No | Yes | Yes | Yes | **Critically low** |
| Guo, M.2019 | Yes | No | Yes | Yes | Yes | Yes | Yes | Yes | Yes | Yes | Yes | Yes | Yes | Yes | Yes | Yes | **Low** |
| Chen, X.2019 | Yes | No | Yes | Yes | Yes | Yes | Yes | Yes | Yes | No | Yes | Yes | No | Yes | Yes | Yes | **Critically low** |
| Chen, J. H.2019 | Yes | No | Yes | Yes | Yes | Yes | Yes | Yes | Yes | No | Yes | Yes | Yes | Yes | Yes | Yes | **Low** |
| Chang, V. C.2019 | Yes | No | Yes | Yes | Yes | Yes | Yes | Yes | Yes | Yes | Yes | Yes | Yes | Yes | Yes | Yes | **Low** |
| Chan, D. S. M.2019 | Yes | No | Yes | Yes | No | No | Yes | Yes | Yes | Yes | Yes | Yes | Yes | Yes | Yes | Yes | **Low** |
| Thakur, A. A.2018 | Yes | No | Yes | Yes | No | No | Yes | Yes | Partial yes | No | Yes | Yes | Yes | Yes | Yes | Yes | **Low** |
| Tang, G. H.2018 | Yes | No | Yes | Yes | Yes | Yes | Yes | Yes | Partial yes | No | Yes | Yes | No | Yes | Yes | Yes | **Critically low** |
| Sun, M.2018 | Yes | No | Yes | Yes | Yes | Yes | Yes | Yes | Partial yes | No | Yes | Yes | Yes | Yes | Yes | Yes | **Low** |
| Shao, J.2018 | Yes | No | Yes | Yes | Yes | Yes | Yes | Yes | Partial yes | No | Yes | Yes | Yes | Yes | Yes | Yes | **Low** |
| Rezaianzadeh, A.2018 | Yes | No | Yes | Yes | Yes | Yes | Yes | Yes | Partial yes | No | Yes | Yes | No | Yes | Yes | Yes | **Critically low** |
| Qiao, Y.2018 | Yes | No | Yes | Yes | No | Yes | Yes | Yes | Yes | Yes | Yes | Yes | No | Yes | Yes | Yes | **Critically low** |
| Liu, K.2018 | Yes | No | Yes | Yes | Yes | Yes | Yes | Yes | Yes | Yes | Yes | Yes | Yes | Yes | Yes | Yes | **Low** |
| Li, Dongyang.2018 | Yes | No | Yes | Yes | Yes | No | Yes | Yes | Yes | No | Yes | Yes | Yes | Yes | Yes | Yes | **Low** |
| Lafranconi, A.2018 | Yes | No | Yes | Yes | Yes | Yes | Yes | Yes | Yes | Yes | Yes | Yes | No | Yes | Yes | Yes | **Critically low** |
| Kim, A. S.2018 | Yes | No | Yes | Yes | Yes | No | Yes | Yes | Yes | Yes | Yes | Yes | No | Yes | Yes | Yes | **Critically low** |
| Hidayat, K.2018 | Yes | No | Yes | Yes | Yes | Yes | Yes | Yes | Yes | No | Yes | Yes | Yes | Yes | Yes | Yes | **Low** |
| Du, R.2018 | Yes | No | Yes | Yes | Yes | Yes | Yes | Yes | No | Yes | Yes | No | No | Yes | Yes | Yes | **Critically low** |
| Deng, Y.2018 | Yes | No | Yes | Yes | Yes | Yes | Yes | Yes | Partial yes | No | Yes | Yes | No | Yes | Yes | Yes | **Critically low** |
| Cordina-D, E.2018 | Yes | No | Yes | No | No | No | Yes | Yes | No | No | Yes | No | No | Yes | No | Yes | **Critically low** |
| Chen, H.2018 | Yes | No | Yes | Yes | Yes | Yes | Yes | Yes | Yes | No | Yes | Yes | Yes | Yes | Yes | Yes | **Low** |
| Xu, J.2017 | Yes | No | Yes | Yes | Yes | Yes | Yes | Yes | No | Yes | Yes | No | No | Yes | Yes | Yes | **Critically low** |
| Schlesinger,S.2017 | Yes | No | Yes | Yes | Yes | Yes | Yes | Yes | No | Yes | Yes | No | No | Yes | Yes | Yes | **Critically low** |
| Nindrea, R. D.2017 | Yes | No | Yes | Yes | No | No | Yes | Yes | No | No | Yes | No | No | Yes | No | Yes | **Critically low** |
| Ni, H. 2017 | Yes | No | Yes | Yes | Yes | Yes | Yes | Yes | Yes | No | Yes | Yes | Yes | Yes | Yes | Yes | **Low** |
| Nagel, G.2017 | Yes | No | Yes | Yes | Yes | Yes | Yes | Yes | Yes | Yes | Yes | Yes | Yes | Yes | Yes | Yes | **Low** |
| Kolahdouz M.R.2017 | Yes | No | Yes | Yes | Yes | Yes | Yes | Yes | Yes | Yes | Yes | Yes | Yes | Yes | Yes | Yes | **Low** |
| Karasneh, R. A.2017 | Yes | No | Yes | Yes | Yes | Yes | Yes | Yes | Partial yes | Yes | Yes | Yes | No | Yes | Yes | Yes | **Critically low** |
| Jia, Y.2017 | Yes | No | Yes | Yes | Yes | Yes | Yes | Yes | Partial yes | Yes | Yes | Yes | No | Yes | Yes | Yes | **Critically low** |
| Chen, Y.2017 | Yes | No | Yes | Yes | Yes | Yes | Yes | Yes | Partial yes | Yes | Yes | Yes | No | Yes | Yes | Yes | **Critically low** |
| Neil-S S. E.2017 | Yes | No | Yes | Yes | Yes | Yes | Yes | Yes | Yes | Yes | Yes | Yes | No | Yes | No | Yes | **Critically low** |
| Zhou, Y.2016 | Yes | No | Yes | Yes | Yes | No | Yes | Yes | Yes | No | Yes | Yes | Yes | Yes | Yes | Yes | **Low** |
| Wang, M.2016 | Yes | No | Yes | Yes | Yes | Yes | Yes | Yes | Yes | Yes | Yes | Yes | Yes | Yes | Yes | Yes | **Low** |
| Sun, Shanwen.2016 | Yes | No | Yes | Yes | Yes | Yes | Yes | Yes | Yes | Yes | Yes | Yes | Yes | Yes | Yes | Yes | **Low** |
| Neilson, H. K.2016 | Yes | Yes | Yes | Yes | Yes | No | Yes | Yes | Yes | Yes | Yes | Yes | Yes | Yes | Yes | Yes | **Moderate** |
| Mullie, P.2016 | Yes | No | Yes | Yes | Yes | Yes | Yes | Yes | Yes | Yes | Yes | Yes | Yes | Yes | Yes | Yes | **Low** |
| Li, C.2016 | Yes | No | Yes | Yes | Yes | Yes | Yes | Yes | Yes | No | Yes | Yes | Yes | Yes | Yes | Yes | **Low** |
| Lei, L.2016 | Yes | No | Yes | Yes | Yes | Yes | Yes | Yes | Yes | Yes | Yes | Yes | Yes | Yes | Yes | Yes | **Low** |
| Gong, Wei-Jing2016 | Yes | No | Yes | Yes | Yes | Yes | Yes | Yes | Yes | Yes | Yes | Yes | Yes | Yes | Yes | Yes | **Low** |
| Fabiani, R.2016 | Yes | No | Yes | Yes | Yes | Yes | Yes | Yes | Yes | Yes | Yes | Yes | Yes | Yes | Yes | Yes | **Low** |
| Chen, Jia-Yan2016 | Yes | No | Yes | Yes | Yes | Yes | Yes | Yes | Yes | No | Yes | Yes | No | Yes | Yes | No | **Critically low** |
| Cao, Y.2016 | Yes | No | Yes | Yes | Yes | Yes | Yes | Yes | Yes | Yes | Yes | Yes | Yes | Yes | Yes | Yes | **Low** |
| Cai, X.2016 | Yes | No | Yes | Yes | No | Yes | Yes | Yes | Yes | No | Yes | Yes | Yes | Yes | Yes | No | **Low** |
| Bae, J. M.2016 | Yes | No | Yes | Yes | Yes | Yes | Yes | Yes | Yes | Yes | Yes | Yes | No | Yes | Yes | Yes | **Critically low** |
| Zhang, J.2015 | Yes | No | Yes | Yes | Yes | Yes | Yes | Yes | Yes | Yes | Yes | Yes | Yes | Yes | Yes | Yes | **Low** |
| Xin, Y.2015 | Yes | No | Yes | Yes | Yes | Yes | Yes | Yes | Yes | No | Yes | Yes | No | Yes | Yes | Yes | **Critically low** |
| Wu, Y. C.2015 | Yes | No | Yes | Yes | Yes | Yes | Yes | Yes | Partial yes | No | Yes | Yes | Yes | Yes | Yes | Yes | **Low** |
| Wang, T.2015 | Yes | No | Yes | Yes | Yes | Yes | Yes | Yes | Partial yes | No | Yes | Yes | No | Yes | Yes | Yes | **Critically low** |
| Touvier, M.2015 | Yes | No | Yes | Yes | Yes | No | Yes | Yes | Yes | Yes | Yes | Yes | Yes | Yes | Yes | Yes | **Low** |
| Shi, Yun.2015 | Yes | No | Yes | Yes | Yes | Yes | Yes | Yes | Yes | Yes | Yes | Yes | Yes | Yes | Yes | Yes | **Low** |
| Larsson, S. C.2015 | Yes | No | Yes | Yes | No | No | Yes | Yes | No | No | Yes | No | No | Yes | Yes | Yes | **Critically low** |
| Hu, F.2015 | Yes | No | Yes | Yes | Yes | Yes | Yes | Yes | No | Yes | Yes | No | Yes | Yes | Yes | Yes | **Critically low** |
| He, X. Y.2015 | Yes | No | Yes | Yes | No | Yes | Yes | Yes | Yes | No | Yes | Yes | Yes | Yes | Yes | Yes | **Low** |
| Guo, J.2015 | Yes | No | Yes | Yes | Yes | Yes | Yes | Yes | No | No | Yes | No | Yes | Yes | Yes | Yes | **Critically low** |
| de Pedro, M.2015 | Yes | No | Yes | Yes | No | No | Yes | Yes | No | No | Yes | No | No | Yes | Yes | Yes | **Critically low** |
| Chen, Z.2015 | Yes | No | Yes | Yes | Yes | Yes | Yes | Yes | No | No | Yes | No | No | Yes | Yes | Yes | **Critically low** |
| Bae, J. M.2015 | Yes | No | Yes | Yes | No | No | Yes | Yes | Yes | Yes | Yes | Yes | Yes | Yes | Yes | Yes | **Low** |
| Zhao, G.2014 | Yes | No | Yes | No | No | No | Yes | Yes | No | No | Yes | No | No | Yes | Yes | Yes | **Critically low** |
| Yu, F.2014 | Yes | No | Yes | Yes | Yes | Yes | Yes | Yes | Partial yes | Yes | Yes | Yes | Yes | Yes | Yes | Yes | **Low** |
| Yang, Wan-Shui.2014 | Yes | No | Yes | Yes | Yes | Yes | Yes | Yes | Partial yes | Yes | Yes | Yes | Yes | Yes | Yes | Yes | **Low** |
| Sergentanis, T. N.2014 | Yes | No | Yes | Yes | Yes | Yes | Yes | Yes | Yes | No | Yes | Yes | No | No | Yes | Yes | **Critically low** |
| Park, J. H.2014 | Yes | No | Yes | Yes | Yes | Yes | Yes | Yes | No | Yes | Yes | No | No | Yes | Yes | Yes | **Critically low** |
| Nie, X. C.2014 | Yes | No | Yes | Yes | Yes | Yes | Yes | Yes | No | Yes | Yes | No | No | Yes | Yes | Yes | **Critically low** |
| Lv, M.2014 | Yes | No | Yes | Yes | Yes | Yes | Yes | Yes | Yes | Yes | Yes | Yes | Yes | Yes | Yes | Yes | **Low** |
| Liu, X. O.2014 | Yes | No | Yes | Yes | Yes | No | Yes | Yes | Yes | Yes | Yes | Yes | No | Yes | Yes | Yes | **Critically low** |
| Chen, C.2014 | Yes | No | Yes | Yes | Yes | Yes | Yes | Yes | Yes | Yes | Yes | Yes | Yes | Yes | Yes | Yes | **Low** |
| Zheng, J. S.2013 | Yes | No | Yes | Yes | Yes | No | Yes | Yes | Yes | Yes | Yes | Yes | Yes | Yes | Yes | Yes | **Low** |
| Yang, Y.2013 | Yes | No | Yes | Yes | Yes | Yes | Yes | Yes | Yes | Yes | Yes | Yes | Yes | Yes | Yes | Yes | **Low** |
| Warren, G. W.2013 | Yes | No | Yes | Yes | No | No | Yes | Yes | No | No | Yes | No | No | Yes | Yes | Yes | **Critically low** |
| Wu, W.2013 | Yes | No | Yes | Yes | Yes | Yes | Yes | Yes | No | No | Yes | No | Yes | Yes | Yes | Yes | **Critically low** |
| Song, J. K.2013 | Yes | No | Yes | Yes | Yes | Yes | Yes | Yes | No | No | Yes | No | No | Yes | Yes | Yes | **Critically low** |
| Qu, X.2013 | Yes | No | Yes | Yes | Yes | Yes | Yes | Yes | Partial yes | Yes | Yes | Yes | No | Yes | Yes | Yes | **Critically low** |
| Liu, X.2013 | Yes | No | Yes | Yes | No | Yes | Yes | Yes | No | No | Yes | No | No | Yes | Yes | Yes | **Critically low** |
| Lin, Y.2013 | Yes | No | Yes | Yes | Yes | Yes | Yes | Yes | No | No | Yes | No | No | Yes | Yes | Yes | **Critically low** |
| Kim, J. S.2013 | Yes | No | Yes | Yes | Yes | Yes | Yes | Yes | No | Yes | Yes | No | No | Yes | Yes | Yes | **Critically low** |
| Kamdar, B. B.2013 | Yes | No | Yes | Yes | Yes | Yes | Yes | Yes | No | No | Yes | No | No | Yes | Yes | Yes | **Critically low** |
| Heikkilä, K.2013 | Yes | No | Yes | No | No | No | Yes | Yes | No | Yes | Yes | No | No | Yes | Yes | Yes | **Critically low** |
| Hui, C.2013 | Yes | No | Yes | Yes | No | No | Yes | Yes | No | Yes | Yes | No | No | Yes | Yes | Yes | **Critically low** |
| Gao, Y.2013 | Yes | No | Yes | Yes | Yes | Yes | Yes | Yes | Partial yes | Yes | Yes | Yes | No | Yes | Yes | Yes | **Critically low** |
| Bonifazi, M.2013 | Yes | No | Yes | Yes | Yes | Yes | Yes | Yes | Partial yes | Yes | Yes | Yes | No | Yes | Yes | Yes | **Critically low** |
| Amadou, A.2013 | Yes | No | Yes | Yes | No | Yes | Yes | Yes | Yes | No | Yes | Yes | No | Yes | Yes | Yes | **Critically low** |
| Undela, K.2012 | Yes | No | Yes | Yes | Yes | Yes | Yes | Yes | Yes | No | Yes | Yes | No | Yes | Yes | Yes | **Critically low** |
| Ni, X. J.2012 | Yes | No | Yes | No | No | Yes | Yes | Yes | No | No | Yes | No | No | Yes | Yes | Yes | **Critically low** |
| Hu, F.2012 | Yes | No | Yes | Yes | No | Yes | Yes | Yes | No | No | Yes | No | Yes | Yes | Yes | Yes | **Critically low** |
| Du, X.2012 | Yes | No | Yes | Yes | No | Yes | Yes | Yes | No | No | Yes | No | No | Yes | Yes | Yes | **Critically low** |
| Aune, D.2012 | Yes | Yes | Yes | Yes | No | Yes | Yes | Yes | No | Yes | Yes | No | No | Yes | Yes | Yes | **Critically low** |
| Aune, D.2012 | Yes | Yes | Yes | Yes | Yes | Yes | Yes | Yes | No | No | Yes | No | No | Yes | Yes | Yes | **Critically low** |
| Angelousi, A.G.2012 | Yes | No | Yes | Yes | No | Yes | Yes | Yes | Yes | No | Yes | Yes | No | Yes | Yes | Yes | **Critically low** |
| Walker, K.2011 | Yes | No | Yes | Yes | No | Yes | Yes | Yes | No | No | Yes | No | No | Yes | Yes | Yes | **Critically low** |
| Dong, J. Y.2011 | Yes | No | Yes | Yes | Yes | Yes | Yes | Yes | Yes | Yes | Yes | Yes | Yes | Yes | Yes | Yes | **Low** |
| Chan, A. L.2011 | Yes | No | Yes | Yes | Yes | Yes | Yes | Yes | No | Yes | Yes | No | No | Yes | Yes | Yes | **Critically low** |
| Key, T. J.2010 | Yes | No | Yes | Yes | No | No | Yes | Yes | No | Yes | Yes | No | No | Yes | No | Yes | **Critically low** |
| Buck, K.2010 | Yes | No | Yes | Yes | Yes | Yes | Yes | Yes | No | Yes | Yes | No | No | Yes | Yes | Yes | **Critically low** |
| Brennan, S. F.2010 | Yes | No | Yes | Yes | Yes | Yes | Yes | Yes | No | Yes | Yes | No | No | Yes | Yes | Yes | **Critically low** |
| Xu, X.2009 | Yes | No | Yes | Yes | Yes | Yes | Yes | Yes | No | No | Yes | No | No | Yes | Yes | Yes | **Critically low** |
| Vojtechova, P.2009 | Yes | No | Yes | Yes | Yes | Yes | Yes | Yes | Partial yes | No | Yes | Yes | No | Yes | Yes | No | **Critically low** |
| Sadri, G.2007 | Yes | No | Yes | Yes | No | Yes | Yes | Yes | No | No | Yes | No | No | Yes | Yes | No | **Critically low** |
| Xue, F.2007 | Yes | No | Yes | Yes | No | No | Yes | Yes | No | Yes | Yes | No | No | Yes | Yes | Yes | **Critically low** |
| Takkouche, B.2005 | Yes | No | Yes | Yes | Yes | Yes | Yes | Yes | Partial yes | Yes | Yes | Yes | No | Yes | Yes | Yes | **Critically low** |
| Renehan, A. G.2004 | Yes | No | Yes | Yes | Yes | Yes | Yes | Yes | Partial yes | No | Yes | Yes | Yes | Yes | No | Yes | **Critically low** |
| Boyd, N. F.2003 | Yes | No | Yes | Yes | No | No | Yes | Yes | Partial yes | No | Yes | Yes | No | Yes | Yes | No | **Critically low** |
| Khuder, S. A.2000 | Yes | No | Yes | Yes | Yes | Yes | Yes | Yes | No | No | Yes | No | No | Yes | Yes | No | **Critically low** |

1. Did the research questions and inclusion criteria for the review include the components of PICO? 2. Did the report of the review contain an explicit statement that the review methods were established prior to the conduct of the review and did the report justify any significant deviations from the protocol 3. Did the review authors explain their selection of the study designs for inclusion in the review? 4. Did the review authors use a comprehensive literature search strategy? 5. Did the review authors perform study selection in duplicate? 6. Did the review authors perform data extraction in duplicate? 7. Did the review authors provide a list of excluded studies and justify the exclusions? 8. Did the review authors describe the included studies in adequate detail? 9. Did the review authors use a satisfactory technique for assessing the risk of bias (RoB) in individual studies that were included in the review? 10. Did the review authors report on the sources of funding for the studies included in the review? 11. If meta-analysis was performed did the review authors use appropriate methods for statistical combination of results? 12. If meta-analysis was performed, did the review authors assess the potential impact of RoB in individual studies on the results of the meta-analysis or other evidence synthesis? 13. Did the review authors account for RoB in individual studies when interpreting/ discussing the results of the review? 14. Did the review authors provide a satisfactory explanation for, and discussion of, any heterogeneity observed in the results of the review? 15. If they performed quantitative synthesis did the review authors carry out an adequate investigation of publication bias (small study bias) and discuss its likely impact on the results of the review? 16. Did the review authors report any potential sources of conflict of interest, including any funding they received for conducting the review? `

**Supplemental Table 6:** Details of evidence grading for meta-analyses of risk factors for breast cancer incidence— only cohort studies included*

| **Exposure** | **Exposure contrast** | **N^*^** | **Sample size Cases/Cohort** | **Largest study**^#^ | **Random effects summary**  **Es (95% CI) ^¥^** | **Random**  **P-value**^\|\|^ | **95% Prediction interval** | **Egger’s P**^∞^ | **I^2^ (%)** | **Excess significance**  **P-value**^α^ | **Evidence Grading** |
| --- | --- | --- | --- | --- | --- | --- | --- | --- | --- | --- | --- |
| **Strong evidence** | | | | | | | | | | | |
| ***Imageological diagnosis*** | | | | | | | | | | | |
| Breast density | highest vs. lowest category | 7 | 1356/109050 | [2.67, 3.37] | 2.889[2.569,3.249] | 3.06e-70 | [1.246, 6.702] | 2.12e-01 | 3.311 | 4.75e-01 | Strong |
| ***Use of medical/hormonal therapy*** | | | | | | | | | | | |
| Cardiac glycosides use | ever vs. never | 6 | 53971/2339718 | [1.321, 1.461] | 1.388[1.327, 1.452] | 7.36e-46 | [1.302, 1.48] | 3.24e-01 | 0 | 8.38e-01 | Strong |
| ***Pre-existing medical conditions and interventions*** | | | | | | | | | | | |
| Atrial fibrillation | ever vs. never | 5 | 11632/442702 | [1.111, 1.211] | 1.179[1.139, 1.221] | 1.36e-20 | [1.115, 1.248] | 6.56e-01 | 0 | 5.45e-01 | Strong |
| ***Dietary intake*** | | | | | | | | | | | |
| Vegetable-fruit-soybean dietary pattern | highest vs. lowest category | 12 | 19398/757364 | [0.795, 1.018] | 0.874[0.83, 0.921] | 4.89e-07 | [0.824, 0.928] | 7.25e-01 | 0 | 6.23e-01 | Strong |
| ***Anthropometric indices*** | | | | | | | | | | | |
| BMI>=25 | high vs. low, postmenopausal | 18 | 28671/1156200 | [0.77, 0.96] | 0.858[0.808, 0.912] | 6.53e-07 | [0.804, 0.916] | 2.91e-01 | 0 | 3.92e-01 | Strong |
| **Highly suggestive evidence** | | | | | | | | | | | |
| ***Life behaviour*** | | | | | | | | | | | |
| Education level | highest vs. lowest category | 18 | 194654/10233064 | [1.139, 1.289] | 1.221[1.144, 1.303] | 2.19e-09 | [0.959, 1.554] | 8.27e-01 | 84.586 | 6.21e-01 | Highly suggestive |
| Physical activity | highest vs. lowest category | 61 | 258977/11213814 | [0.91, 0.987] | 0.876[0.851, 0.901] | 1.97e-20 | [0.754, 1.016] | 6.22e-03 | 57.223 | 9.82e-07 | Highly suggestive |
| Smoking | ever vs. never | 16 | 62396/2044596 | [1.029, 1.112] | 1.107 [1.075, 1.14] | 1.57e-11 | [1.029, 1.192] | 3.99e-01 | 31.376 | 3.58e-03 | Highly suggestive |
| Light exposure at night | highest vs. lowest category | 9 | 46760/793292 | [1.008, 1.25] | 1.106[1.065, 1.149] | 2.29e-07 | [1.056, 1.158] | 7.77e-01 | 0 | 2.91e-02 | Highly suggestive |
| ***Anthropometric indices*** | | | | | | | | | | | |
| BMI iya | per 5 kg/m2 | 24 | 33606/2000548 | [0.923, 0.973] | 0.86[0.819, 0.903] | 1.30e-09 | [0.718, 1.03] | 2.19e-04 | 81.253 | 3.65e-09 | Highly suggestive |
| ***Use of medical/hormonal therapy*** | | | | | | | | | | | |
| Antipsychotic use | ever vs. never | 14 | 71679/3430242 | [1.119, 1.289] | 1.204[1.12, 1.293] | 3.95e-07 | [0.935, 1.549] | 5.02e-01 | 79.2 | 1.20e-01 | Highly suggestive |
| Calcium channel blockers | ever vs. never | 3 | 2697/166459 | [1.21, 1.42] | 1.306[1.207, 1.412] | 3.02e-11 | [0.784, 2.174] | 1.86e-01 | 0 | 6.28e-01 | Highly suggestive |
| Antibiotic use | ever vs. never； | 2 | 18678/2158968 | [1.09, 1.17] | 1.132[1.093, 1.172] | 5.55e-12 | < 3 studies | < 3 studies | 0 | NA | Highly suggestive |
| **Suggestive evidence** | | | | | | | | | | | |
| ***Dietary intake*** | | | | | | | | | | | |
| Alcohol | highest vs. lowest category | 4 | 1251/159858 | [1.16, 2.67] | 1.842[1.426, 2.379] | 2.87e-06 | [1.05, 3.23] | 3.41e-01 | 0 | 2.39e-01 | Suggestive |
| Total meat intake | per 100 g/day | 25 | 190014/3979157 | [0.932, 1.052] | 1.083[1.033, 1.135] | 9.54e-04 | [0.922, 1.272] | 1.28e-03 | 70.622 | 2.31e-10 | Suggestive |
| Red meat intake | per 100 g/day | 23 | 90742/3564476 | [1.007, 1.11] | 1.083[1.035, 1.134] | 6.10e-04 | [0.92, 1.275] | 5.36e-02 | 58.011 | 2.61e-02 | Suggestive |
| Fruit intake | per 100 g/day | 19 | 971972343888 | [0.918, 0.968] | 0.968[0.949, 0.987] | 9.79e-04 | [0.907, 1.032] | 5.02e-02 | 65.369 | 3.39e-01 | Suggestive |
| Fiber intake | highest vs. lowest category | 24 | 122005/4169698 | [0.914, 0.971] | 0.933[0.903, 0.964] | 3.02e-05 | [0.853, 1.02] | 3.64e-01 | 46.143 | 1.52e-02 | Suggestive |
| Selenium | ever vs. never | 12 | 7558/142580 | [0.519, 0.919] | 0.893[0.849, 0.939] | 1.14e-05 | [0.843, 0.946] | 8.15e-01 | 0 | 4.61e-01 | Suggestive |
| Tofu intake | highest vs. lowest category | 14 | 17075/136740 | [0.708, 0.982] | 0.769[0.674, 0.878] | 9.66e-05 | [0.512, 1.157] | 2.85e-02 | 57.336 | 9.55e-02 | Suggestive |
| Adherence score | highest vs. lowest category | 7 | 72420/1449105 | [0.626, 0.869] | 0.736[0.646, 0.839] | 4.33e-06 | [0.506, 1.07] | 8.89e-01 | 65.023 | 6.66e-01 | Suggestive |
| ***Imageological diagnosis*** | | | | | | | | | | | |
| Bone mineral density | highest vs. lowest category | 9 | 4445/219806 | [1.17, 1.463] | 1.662[1.282, 2.155] | 1.24e-04 | [0.734, 3.764] | 2.56e-01 | 73.498 | 1.30e-01 | Suggestive |
| ***Life behaviour*** | | | | | | | | | | | |
| Famine exposure | ever vs. never | 44 | 10844/185341 | [0.818, 1.444] | 1.294[1.174, 1.425] | 1.99e-07 | [0.968, 1.729] | 8.02e-01 | 8.977 | 7.79e-01 | Suggestive |
| Lifestyle Quality Indices | highest vs. lowest category | 17 | 43794/1170291 | [0.795, 1.217] | 0.772[0.711, 0.838] | 7.47e-10 | [0.595, 1.002] | 9.65e-02 | 67.781 | 2.57e-02 | Suggestive |
| ***Anthropometric indices*** | | | | | | | | | | | |
| BMI | highest vs. lowest category | 28 | 51544/5048361 | [0.712, 0.972] | 1.209[1.083, 1.35] | 7.16e-04 | [0.741, 1.973] | 2.26e-01 | 78.896 | 1.99e-01 | Suggestive |
| Fat mass | highest vs. lowest category | 7 | 5956/265841 | [1.43, 1.79] | 1.445[1.226, 1.703] | 1.16e-05 | [0.883, 2.364] | 8.94e-01 | 68.895 | 2.07e-02 | Suggestive |
| Weight gain | highest vs. lowest category | 16 | 20536/864957 | [0.549, 1.11] | 1.415[1.273, 1.574] | 1.35e-10 | [0.996, 2.012] | 5.58e-01 | 62.317 | 6.66e-01 | Suggestive |
| BMI | per 5 kg/m2 | 32 | 59213/4132649 | [1.02, 1.04] | 0.94[0.912, 0.969] | 6.10e-05 | [0.813, 1.087] | 2.73e-03 | 92.378 | 9.33e-15 | Suggestive |
| BMI<25 | high vs. low, postmenopausal | 18 | 29860/1221731 | [0.867, 1.047] | 0.853[0.785, 0.927] | 1.80e-04 | [0.674, 1.078] | 1.05e-02 | 39.226 | 4.67e-02 | Suggestive |
| ***Biomarkers*** | | | | | | | | | | | |
| IGF1 | highest vs. lowest category | 17 | 5717/17070 | [1.065, 1.675] | 1.252[1.133, 1.383] | 9.68e-06 | [1.124, 1.395] | 6.62e-01 | 0 | 7.64e-01 | Suggestive |
| ***Use of medical/hormonal therapy*** | | | | | | | | | | | |
| Aspirin intake | ever vs. never | 64 | 134883/5415706 | [1.04, 1.25] | 0.936[0.901, 0.972] | 6.31e-04 | [0.743, 1.179] | 7.60e-02 | 81.264 | 2.77e-08 | Suggestive |
| ***Pre-existing medical conditions and interventions*** | | | | | | | | | | | |
| Antibody | ever vs. never | 8 | 1687/7727 | [1.104, 3.523] | 2.522[1.666, 3.816] | 1.21e-05 | [0.747, 8.517] | 2.50e-02 | 65.786 | 2.44e-06 | Suggestive |
| Periodontal disease | ever vs. never | 12 | 7424/342916 | [1.059, 1.201] | 1.235[1.108, 1.376] | 1.39e-04 | [0.954, 1.599] | 5.19e-02 | 52.106 | 2.84e-02 | Suggestive |
| Metabolic Syndrome | ever vs. never | 17 | 25569/1182195 | [0.904, 0.992] | 1.198[1.08, 1.328] | 6.25e-04 | [0.824, 1.74] | 1.55e-03 | 76.861 | 5.08e-09 | Suggestive |
| Hyperthyroidism | ever vs. never | 13 | 17433/4399480 | [1.118, 1.358] | 1.15[1.082, 1.223] | 7.44e-06 | [0.998, 1.327] | 2.06e-01 | 36.486 | 1.25e-01 | Suggestive |
| Bariatric Surgery | ever vs. never | 14 | 28114/2508424 | [0.459, 0.606] | 0.565[0.444, 0.721] | 4.06e-06 | [0.211, 1.518] | 9.13e-01 | 96.817 | 7.80e-01 | Suggestive |
| **Weak evidence** | | | | | | | | | | | |
| ***Dietary intake*** | | | | | | | | | | | |
| DII | per 1‐point | 6 | 15007/172704 | [1.008, 1.759] | 1.325[1.043, 1.683] | 2.14e-02 | [0.586, 2.996] | 3.43e-01 | 87.333 | 1.22e-02 | Weak |
| Wine Drinking | highest vs. lowest category | 9 | 10220/541052 | [0.654, 1.492] | 1.222[1.037, 1.439] | 1.66e-02 | [0.779, 1.917] | 7.99e-01 | 56.734 | 2.53e-02 | Weak |
| Processed meat intake | per 50 g/day | 17 | 92323/2522068 | [0.438, 1.219] | 1.178[1.04, 1.333] | 9.77e-03 | [0.788, 1.761] | 1.70e-01 | 63.227 | 7.91e-02 | Weak |
| SSBs | per 250mg/day | 7 | 14886/455697 | [0.968, 1.038] | 1.171[1.004, 1.365] | 4.38e-02 | [0.779, 1.76] | 3.66e-02 | 64.874 | 5.02e-01 | Weak |
| Processed meat intake | highest vs. lowest category | 20 | 60327/2350966 | [0.942, 1.122] | 1.08[1.026, 1.137] | 3.16e-03 | [0.906, 1.287] | 3.90e-01 | 59.682 | 2.03e-01 | Weak |
| Glycemic index/Glycemic load | highest vs. lowest category | 14 | 66280/1162453 | [0.988, 1.119] | 1.049[1.013, 1.086] | 7.10e-03 | [1.009, 1.09] | 5.75e-01 | 0 | 6.55e-01 | Weak |
| Glycemic index | highest vs. lowest category | 17 | 80902/2912944 | [0.955, 1.087] | 1.047[1.007, 1.089] | 2.23e-02 | [0.934, 1.174] | 1.87e-01 | 43.595 | 2.56e-01 | Weak |
| Glycemic index | per 10 units/day | 10 | 36826/1110003 | [1.008, 1.118] | 1.037[1.003, 1.072] | 3.37e-02 | [0.965, 1.115] | 3.87e-01 | 25.27 | 8.87e-02 | Weak |
| Total fat intake | highest vs. lowest category | 27 | 44079/1797892 | [0.968, 1.209] | 1.104[1.035, 1.177] | 2.47e-03 | [0.883, 1.38] | 9.68e-04 | 62.017 | 1.14e-03 | Weak |
| Vegetable intake | Per 100 g/day | 14 | 76875/1550521 | [0.91, 0.99] | 0.974[0.952, 0.996] | 1.87e-02 | [0.909, 1.043] | 1.49e-03 | 58.911 | 5.98e-04 | Weak |
| Soy intake | per 30 g/day | 7 | 4122/155528 | [0.691, 1.171] | 0.967[0.94, 0.994] | 1.63e-02 | [0.932, 1.002] | 5.90e-01 | 0 | 4.97e-01 | Weak |
| Soy isoflavone | per 10mg/day | 9 | 10229/631498 | [0.896, 1.057] | 0.967[0.945, 0.99] | 5.18e-03 | [0.926, 1.01] | 6.49e-01 | 15.368 | 4.32e-01 | Weak |
| Coffee intake | per 10mg/day | 21 | 99917/2927384 | [0.917, 1.019] | 0.961[0.928, 0.996] | 2.83e-02 | [0.884, 1.046] | 6.02e-01 | 23.349 | 6.00e-01 | Weak |
| Vitamin D intake | highest vs. lowest category | 12 | 24608/886437 | [0.943, 1.142] | 0.955[0.915, 0.997] | 3.41e-02 | [0.91, 1.002] | 7.36e-01 | 0 | 8.58e-01 | Weak |
| Cheese intake | per 30 g/day | 10 | 46744/1419872 | [0.948, 1.018] | 0.952[0.909, 0.996] | 3.30e-02 | [0.839, 1.079] | 2.19e-01 | 75.285 | 3.81e-01 | Weak |
| B-carotene | per 5000ug/day | 10 | 18191/825911 | [0.908, 1.018] | 0.95[0.914, 0.988] | 9.55e-03 | [0.908, 0.994] | 6.50e-01 | 0 | 5.92e-01 | Weak |
| Flavonols | per 5000ug/day | 6 | 6325/515174 | [0.884, 1.034] | 0.942[0.897, 0.99] | 1.79e-02 | [0.879, 1.01] | 3.72e-01 | 0 | 4.76e-01 | Weak |
| Dietary calcium intake | per 350mg/day | 6 | 47823/1575204 | [0.97, 0.99] | 0.937[0.888, 0.988] | 1.66e-02 | [0.818, 1.073] | 1.33e-02 | 64.949 | 1.52e-06 | Weak |
| Dietary folate intake | highest vs. lowest category | 23 | 42208/1191707 | [0.832, 1.012] | 0.934[0.88, 0.991] | 2.39e-02 | [0.754, 1.156] | 1.32e-01 | 56.156 | 4.28e-01 | Weak |
| Prudent/healthy dietary pattern | highest vs. lowest category | 8 | 13885/465891 | [0.68, 1.19] | 0.928[0.871, 0.989] | 2.04e-02 | [0.83, 1.037] | 3.17e-01 | 12.242 | 6.17e-01 | Weak |
| Fruit intake | highest vs. lowest category | 12 | 25958/1264887 | [0.93, 1.01] | 0.948[0.907, 0.991] | 1.77e-02 | [0.877, 1.025] | 2.84e-01 | 11.672 | 6.42e-01 | Weak |
| Adherence score | per 1‐point | 5 | 68990/1441887 | [0.876, 0.938] | 0.917[0.863, 0.974] | 5.15e-03 | [0.742, 1.134] | 7.85e-01 | 82.809 | 8.75e-01 | Weak |
| A-carotene | highest vs. lowest category | 5 | 11447/363730 | [0.832, 1.06] | 0.913[0.848, 0.982] | 1.45e-02 | [0.81, 1.028] | 7.68e-01 | 0 | 8.62e-01 | Weak |
| Vitamin B2 | highest vs. lowest category | 10 | 20100/622953 | [0.882, 1.042] | 0.899[0.819, 0.986] | 2.46e-02 | [0.712, 1.134] | 1.71e-01 | 42.502 | 7.09e-01 | Weak |
| Fruits and vegetables intake | highest vs. lowest category | 6 | 5206/188188 | [0.76, 1.09] | 0.89[0.802, 0.989] | 3.01e-02 | [0.768, 1.033] | 5.85e-01 | 0 | 8.39e-01 | Weak |
| Total dairy food intake | highest vs. lowest category | 10 | 19339/662579 | [0.882, 1.042] | 0.876[0.795, 0.965] | 7.22e-03 | [0.694, 1.106] | 3.63e-02 | 41.967 | 1.94e-01 | Weak |
| Dietary calcium intake | highest vs. lowest category | 7 | 47911/1579901 | [0.912, 1.071] | 0.873[0.77, 0.989] | 3.34e-02 | [0.63, 1.208] | 3.27e-02 | 58.844 | 1.27e-02 | Weak |
| Vegetarians | yes vs. no | 4 | 471/558626 | [0.539, 0.99] | 0.849[0.722, 0.998] | 4.68e-02 | [0.503, 1.431] | 8.40e-01 | 29.043 | 3.84e-02 | Weak |
| Higher Mushroom Consumption: | highest vs. lowest category | 3 | 11105/100773 | [0.77, 1.03] | 0.83[0.705, 0.977] | 2.54e-02 | [0.175, 3.941] | 1.43e-01 | 37.939 | 5.28e-01 | Weak |
| ***Life behaviour*** | | | | | | | | | | | |
| Negative Emotions | ever vs. never | 9 | 2080/129621 | [0.968, 1.078] | 1.826[1.249, 2.668] | 1.87e-03 | [0.568, 5.869] | 4.34e-03 | 81.237 | 3.61e-06 | Weak |
| Flight attendants | yes vs. nor | 4 | 680/22410 | [1.324, 1.693] | 1.425[1.321, 1.537] | 4.31e-20 | [1.207, 1.683] | 5.77e-01 | 0 | 5.19e-01 | Weak |
| Sedentary work | yes vs. no | 13 | 82317/3318327 | [0.669, 1.487] | 1.141[1.04, 1.252] | 5.40e-03 | [0.899, 1.448] | 2.16e-01 | 40.063 | 1.22e-01 | Weak |
| Occupational exposure-organic solvents | ever vs. never | 7 | 27481/1291458 | [1.07, 1.13] | 1.026[1.026, 1.192] | 8.30e-03 | [0.908, 1.347] | 5.41e-01 | 60.581 | 6.19e-03 | Weak |
| Physical activity at a young age | highest vs. lowest category | 9 | 11879/450019 | [0.773, 0.952] | 0.797[0.797, 0.976] | 1.46e-02 | [0.671, 1.159] | 1.78e-01 | 63.346 | 6.63e-03 | Weak |
| Time in the Sun | highest vs. lowest category | 4 | 4019/144807 | [0.758, 0.958] | 0.726[0.726, 0.924] | 1.16e-03 | [0.498, 1.347] | 6.85e-02 | 65.178 | 1.59e-01 | Weak |
| ***Environment*** | | | | | | | | | | | |
| NO2 | per 10 ug/m3 | 12 | 245619/8899009 | [1.003, 1.017] | 1.02[1.008, 1.033] | 1.54e-03 | [0.984, 1.058] | 1.07e-01 | 62.934 | 3.37e-02 | Weak |
| ***Anthropometric indices*** | | | | | | | | | | | |
| weight length | highest vs. lowest category | 3 | 612/6112 | [0.808, 2.429] | 1.708[1.266, 2.304] | 4.63e-04 | [0.245, 11.904] | 8.62e-01 | 0 | 7.01e-01 | Weak |
| BMI>= 30 | highest vs. lowest category | 16 | 25554/1541116 | [0.766, 1.017] | 1.198[1.03, 1.392] | 1.88e-02 | [0.703, 2.039] | 8.00e-01 | 79.765 | 1.57e-01 | Weak |
| Birth Weight | highest vs. lowest category | 12 | 14706/3724312 | [1.023, 1.332] | 1.132[1.011, 1.267] | 3.12e-02 | [0.883, 1.452] | 2.31e-02 | 29.897 | 1.59e-01 | Weak |
| Weight loss | highest vs. lowest category | 16 | 19351/1035071 | [0.63, 1.114] | 0.896[0.834, 0.963] | 2.68e-03 | [0.828, 0.969] | 2.89e-01 | 0 | 4.32e-01 | Weak |
| ***Biomarkers*** | | | | | | | | | | | |
| Serum/plasma iron | highest vs. lowest category | 3 | 4231/377740 | [0.993, 1.273] | 1.364[1.012, 1.838] | 4.18e-02 | [0.05, 37.523] | 3.52e-01 | 69.071 | 5.51e-01 | Weak |
| Plasma prolactin levels | highest vs. lowest category | 6 | 6283/12173 | [0.99, 1.29] | 1.174[1.051, 1.311] | 4.49e-03 | [1.004, 1.373] | 6.85e-01 | 0 | 5.69e-01 | Weak |
| Serum TG levels | highest vs. lowest category | 17 | 33360/1584748 | [0.859, 0.966] | 0.943[0.909, 0.979] | 1.84e-03 | [0.907, 0.982] | 2.61e-01 | 0 | 4.53e-01 | Weak |
| ***Past gynaecological history*** | | | | | | | | | | | |
| Parity | parous vs. nulliparous | 3 | 5278/2464049 | [0.793, 1.191] | 0.903[0.824, 0.991] | 3.14e-02 | [0.496, 1.646] | 7.24e-01 | 0 | 3.41e-01 | Weak |
| ***Use of medical/hormonal therapy*** | | | | | | | | | | | |
| Bisphosphonates | ever vs. never | 8 | 30745/1595461 | [0.818, 0.916] | 0.885[0.814, 0.962] | 4.08e-03 | [0.703, 1.114] | 5.76e-01 | 60.122 | 5.64e-01 | Weak |
| Thiazolidinediones use | ever vs. never | 12 | 7359/1866323 | [0.787, 0.968] | 0.88[0.786, 0.986] | 2.71e-02 | [0.649, 1.193] | 2.29e-01 | 57.398 | 3.15e-01 | Weak |
| Insulins | ever vs. never | 16 | 21727/5703067 | [0.892, 1.052] | 0.86[0.777, 0.952] | 3.53e-03 | [0.643, 1.149] | 4.65e-02 | 63.731 | 6.25e-01 | Weak |
| ***Pre-existing medical conditions and interventions*** | | | | | | | | | | | |
| Obstructive sleep apnea | ever vs. never | 8 | 8300/1398113 | [0.93, 0.99] | 1.357[1.077, 1.709] | 9.68e-03 | [0.615, 2.994] | 3.61e-02 | 94.576 | 1.96e-08 | Weak |
| Autoimmune thyroiditis | ever vs. never | 4 | 646/1085 | [1.779, 4.1] | 2.923[2.133, 4.005] | 2.46e-11 | [1.464, 5.836] | 1.48e-01 | 0 | 1.69e-02 | Weak |
| Goitre | ever vs. never | 4 | 549/1009 | [1.021, 4.87] | 2.824[2.037, 3.914] | 4.61e-10 | [1.379, 5.783] | 8.79e-01 | 0 | 1.63e-01 | Weak |
| Sleep-disordered breathing | ever vs. never | 11 | 233285/7935618 | [0.928, 0.978] | 1.457[1.159, 1.83] | 1.24e-03 | [0.614, 3.459] | 6.70e-01 | 99.658 | 6.89e-03 | Weak |
| Diabetes | ever vs. never | 46 | 241406/17326476 | [1.106, 1.15] | 1.155[1.04, 1.282] | 7.10e-03 | [0.594, 2.244] | 9.21e-02 | 98.515 | 9.77e-01 | Weak |
| CAD | ever vs. never | 5 | 19075/1109286 | [0.641, 0.9] | 0.87[0.776, 0.975] | 1.65e-02 | [0.596, 1.269] | 1.05e-01 | 66.937 | 3.26e-02 | Weak |
| ***congenital factor*** | | | | | | | | | | | |
| Paternal age | per 15 years | 3 | 15853/150312 | [1.007, 1.228] | 1.085[1.008, 1.167] | 3.03e-02 | [0.674, 1.746] | 3.31e-01 | 0 | 3.45e-01 | Weak |
| Twin membership (including monozygotic and dizygotic) | highest vs. lowest category | 2 | 433/175924 | [0.822, 1.002] | 0.918[0.856, 0.986] | 1.88e-02 | < 3 studies | < 3 studies | 0 | NA | Weak |

**Abbreviation：**ACEI/ARB : Angiotensin-converting enzyme inhibitor / angiotensin-receptor；BMI, body mass index; BMI iya, body mass index in young adulthood; CI, confidence interval; HRT, hormone replacement therapy; PA, physical activity; PoMP, postmenopausal; ELF-EMFs: extremely low-frequency electromagnetic fields；DII, dietary inflammatory index; SSBs, sugar-sweetened beverages; SFA, saturated fatty acids ;MUFA, monounsaturated fatty acids; PUFA, polyunsaturated fatty acids; ASBs, artificially sweetened beverages ;UVR, ultraviolet radiation; IA, Induced abortion;IGF-1,Insulin-like growth factor 1 ;IGFBP-3,IGF binding protein 3TG,total triglycerides; TC, total cholesterol；CRP,C-reactive protein; CAD, coronary artery disease; GDM, Gestational diabetes mellitus ;PE, Preeclampsia ;BSO, bilateral salpingo-oophorectom; NA, not available;

**Key:**

*only meta-analyses meeting at least weak grade of evidence listed

**^*^** Number of studies

^#^ 95% confidence interval of largest study (smallest standard error) in each meta-analysis

**^¥^** Random effects refer to summary risk ratio (95% confidence interval) using the random-effects model

^||^ P value of summary random effects estimate

^∞^ P-value from the Egger’s regression asymmetry test

^α^ P value of the excess statistical significance test

All statistical tests were two-sided

Small study effect is based on the P-value from the Egger’s regression asymmetry test (P>0.1) where the random effects summary estimate was larger compared to the point estimate of the largest study in a meta-analysis

**Summary of evidence grading criteria:

| Weak | P<0.05**^\|\|^** |
| --- | --- |
| Suggestive | P<10^-3^**^\|\|^**; >1,000 cases |
| Highly suggestive | P<10^-6^**^\|\|^**; >1,000 cases; P<0.05 of the largest study in a meta-analysis |
| Strong | P<10^-6^**^\|\|^**; >1,000 cases; P<0.05 of the largest study in a meta-analysis; I^2^<50%; no small study effect^¶^; prediction interval excludes the null value; no excess significance bias^†^ |

**Supplemental Table 7:** Details of evidence grading for meta-analyses of risk factors for breast cancer incidence— all study types included*

| **Exposure** | **Exposure contrast** | **N^*^** | **Sample size Cases/Cohort** | **Largest study**^#^ | **Random effects summary**  **Es (95% CI) ^¥^** | **Random**  **P-value**^\|\|^ | **95% Prediction interval** | **Egger’s P**^∞^ | **I^2^ (%)** | **Excess significance P-value**^α^ | **Evidence Grading** |
| --- | --- | --- | --- | --- | --- | --- | --- | --- | --- | --- | --- |
| **Strong evidence** | | | | | | | | | | | |
| ***Pre-existing medical conditions and interventions*** | | | | | | | | | | | |
| Autoimmune thyroiditis | ever vs. never | 5 | 1958/10331 | [0.762, 2.831] | 2.705[2.134, 3.428] | 1.80e-16 | [1.6, 4.573] | 8.63e-01 | 16.594 | 7.84e-01 | Strong |
| Atrial fibrillation | ever vs. never | 5 | 11632/442702 | [1.111, 1.211] | 1.179 [1.139, 1.221] | 1.36e-20 | [1.115, 1.248] | 6.56e-01 | 0 | 5.45e-01 | Strong |
| ***Life behaviour*** | | | | | | | | | | | |
| light exposure at night | highest vs. lowest category | 18 | 125813/859026 | [1.008, 1.25] | 1.125[1.092, 1.159] | 8.29e-15 | [1.089, 1.162] | 3.49e-01 | 0 | 7.17e-02 | Strong |
| ***Use of medical/hormonal therapy*** | | | | | | | | | | | |
| cardiac glycosides use | ever vs. never | 9 | 84770/4314852 | [1.321, 1.461] | 1.332[1.237, 1.434] | 2.21e-14 | [1.147, 1.547] | 3.35e-01 | 22.256 | 9.31e-01 | Strong |
| oral progestagen | ever vs. never | 6 | 1630/44572 | [0.991, 1.38] | 1.283[1.186, 1.389] | 6.06e-10 | [1.076, 1.531] | 9.65e-01 | 25.87 | 8.06e-01 | Strong |
| ***Past gynaecological history*** | | | | | | | | | | | |
| The number of births | >=2 vs.1 | 33 | 85733/232870 | [0.741, 0.841] | 0.788[0.747, 0.832] | 2.52e-18 | [0.651, 0.955] | 8.65e-01 | 49.756 | 2.45e-01 | Strong |
| ***Dietary intake*** | | | | | | | | | | | |
| vegetable-fruit-soybean dietary pattern | ever vs. never | 12 | 19398/757364 | [0.795, 1.018] | 0.874[0.83, 0.921] | 4.89e-07 | [0.824, 0.928] | 7.25e-01 | 0 | 6.23e-01 | Strong |
| ***Anthropometric indices*** | | | | | | | | | | | |
| weight gain | highest vs. lowest category | 16 | 16984/563420 | [1.351, 3.42] | 1.548[1.399, 1.713] | 2.95e-17 | [1.156, 2.071] | 5.42e-02 | 46.971 | 4.90e-01 | Strong |
| ***Biomarkers*** | | | | | | | | | | | |
| Sex hormone binding globulin | highest vs. lowest category | 21 | 5355/15970 | [0.441, 0.841] | 0.648[0.576, 0.73] | 8.93e-13 | [0.521, 0.808] | 7.28e-02 | 9.719 | 7.55e-01 | Strong |
| **Highly suggestive evidence** | | | | | | | | | | | |
| ***Virus infection*** | | | | | | | | | | | |
| HPV | ever vs. never | 46 | 5323/7647 | [1.99, 4.52] | 5.796[4.22, 7.961] | 1.99e-27 | [1.385, 24.257] | 2.93e-02 | 51.098 | 2.13e-02 | Highly suggestive |
| ***Imageological diagnosis*** | | | | | | | | | | | |
| breast density | highest vs. lowest category | 9 | 3237/115630 | [2.67, 3.37] | 2.382 [2.033, 2.79] | 6.43e-27 | [1.553, 3.653] | 1.77e-02 | 55.175 | 9.86e-01 | Highly suggestive |
| ***Life behaviour*** | | | | | | | | | | | |
| education level | highest vs. lowest category | 25 | 197518/10508938 | [1.139, 1.289] | 1.203[1.121, 1.29] | 2.74e-07 | [0.912, 1.586] | 5.35e-01 | 84.743 | 1.25e-01 | Highly suggestive |
| physical activity at a young age | highest vs. lowest category | 29 | 41986/516105 | [0.773, 0.952] | 0.817 [0.762, 0.875] | 1.13e-08 | [0.608, 1.097] | 1.78e-04 | 78.575 | 3.49e-05 | Highly suggestive |
| physical activity | highest vs. lowest category | 115 | 421002/11652526 | [0.91, 0.987] | [0.805, 0.856] | 4.89e-33 | [0.65, 1.06] | 5.38e-05 | 80.718 | 0.00e+00 | Highly suggestive |
| occupational exposure-organic solvents | ever vs. never | 24 | 79687/1590334 | [1.07, 1.13] | 1.18[1.11, 1.255] | 1.18e-07 | [0.939, 1.483] | 9.11e-03 | 76.53 | 1.12e-06 | Highly suggestive |
| ***Past gynaecological history*** | | | | | | | | | | | |
| menopause onset >50 years | ever vs. never | 8 | 2854/14959 | [1.497, 2.098] | 1.864 [1.462, 2.375] | 4.87e-07 | [0.826, 4.202] | 6.35e-01 | 83.573 | 2.13e-01 | Highly suggestive |
| first pregnancy age | highest vs. lowest category | 17 | 3658/13098 | [1.471, 2.081] | 1.669[1.4, 1.991] | 1.21e-08 | [0.88, 3.167] | 3.24e-05 | 79.576 | 1.82e-11 | Highly suggestive |
| age at first birth (AFB) | highest vs. lowest category | 19 | 186204/407178 | [1.222, 1.312] | 1.248 [1.178, 1.321] | 3.19e-14 | [1.038, 1.5] | 6.55e-01 | 72.437 | 1.19e-01 | Highly suggestive |
| ***Anthropometric indices*** | | | | | | | | | | | |
| body fatness at a young age | 5kg/m2 increase of BMI | 24 | 33606/2000548 | [0.923, 0.973] | 0.86 [0.819, 0.903] | 1.30e-09 | [0.718, 1.03] | 2.19e-04 | 81.253 | 3.65e-09 | Highly suggestive |
| ***Use of medical/hormonal therapy*** | | | | | | | | | | | |
| aspirin intake | ever vs. never | 67 | 309495/7320951 | [0.621, 0.951] | 0.914 [0.884, 0.946] | 1.98e-07 | [0.728, 1.148] | 4.19e-03 | 82.311 | 4.48e-10 | Highly suggestive |
| NSAID | ever vs. never | 22 | 91552/442067 | [0.82, 0.88] | 0.808[0.753, 0.867] | 3.45e-09 | [0.591, 1.105] | 2.22e-02 | 88.782 | 4.64e-04 | Highly suggestive |
| **Suggestive evidence** | | | | | | | | | | | |
| ***Virus infection*** | | | | | | | | | | | |
| EBV | ever vs. never | 35 | 3881/7536 | [0.812, 1.301] | 4.779[3.158, 7.231] | 1.34e-13 | [0.718, 31.824] | 1.41e-06 | 75.966 | 8.12e-05 | Suggestive |
| ***Dietary intake*** | | | | | | | | | | | |
| DII | highest vs. lowest category | 14 | 24543/328657 | [0.912, 1.071] | 1.371[1.173, 1.601] | 6.99e-05 | [0.78, 2.409] | 6.84e-03 | 87.093 | 6.66e-03 | Suggestive |
| Wine Drinking | highest vs. lowest category | 26 | 21149/565695 | [0.654, 1.492] | 1.37[1.207, 1.556] | 1.22e-06 | [0.834, 2.251] | 1.17e-01 | 66.286 | 2.56e-03 | Suggestive |
| red meat intake | highest vs. lowest category | 33 | 97061/3577057 | [1.007, 1.11] | 1.143[1.082, 1.208] | 1.63e-06 | [0.908, 1.439] | 2.52e-03 | 67.949 | 2.01e-04 | Suggestive |
| fat intake | highest vs. lowest category | 94 | 109717/3552833 | [0.943, 1.163] | 1.094[1.056, 1.134] | 7.06e-07 | [0.89, 1.346] | 8.05e-06 | 71.379 | 1.44e-15 | Suggestive |
| fruit intake | per 100 g/day | 19 | 97197/2343888 | [0.918, 0.968] | 0.968[0.949, 0.987] | 9.79e-04 | [0.907, 1.032] | 5.02e-02 | 65.369 | 3.39e-01 | Suggestive |
| fiber intake | highest vs. lowest category | 25 | 1122580/4172085 | [0.914, 0.971] | 0.93[0.899, 0.962] | 2.93e-05 | [0.842, 1.027] | 3.64e-01 | 52.221 | 1.53e-01 | Suggestive |
| adherence score | per 1‐point | 7 | 72420/1449105 | [0.876, 0.938] | 0.897[0.85, 0.947] | 7.50e-05 | [0.756, 1.065] | 9.68e-01 | 81.58 | 6.89e-01 | Suggestive |
| fruit intake | highest vs. lowest category | 29 | 41889/1308829 | [0.93, 1.01] | 0.876[0.827, 0.927] | 5.96e-06 | [0.709, 1.082] | 4.28e-02 | 59.078 | 7.33e-02 | Suggestive |
| folate intake | highest vs. lowest category | 37 | 51120/1398103 | [0.832, 1.012] | 0.867[0.803, 0.935] | 2.39e-04 | [0.594, 1.264] | 1.98e-03 | 74.57 | 9.08e-02 | Suggestive |
| vitamin C intake | highest vs. lowest category | 44 | 120647/2076170 | [0.918, 1.129] | 0.852[0.795, 0.914] | 6.63e-06 | [0.598, 1.214] | 6.82e-03 | 80.486 | 6.80e-11 | Suggestive |
| soy intake | highest vs. lowest category | 24 | 29382/521578 | [0.864, 1.123] | 0.85[0.783, 0.921] | 8.33e-05 | [0.606, 1.191] | 3.56e-01 | 76.103 | 1.65e-02 | Suggestive |
| total b-carotene | highest vs. lowest category | 35 | 14162/1012872 | [0.851, 1.27] | 0.839[0.777, 0.905] | 5.41e-06 | [0.6, 1.173] | 1.61e-03 | 63.026 | 8.54e-02 | Suggestive |
| vegetable intake | highest vs. lowest category | 47 | 48835/522137 | [0.89, 1.31] | 0.79[0.733, 0.853] | 1.18e-09 | [0.533, 1.171] | 1.55e-04 | 77.036 | 9.93e-04 | Suggestive |
| Vegetable Oils | highest vs. lowest category | 27 | 39078/572653 | [0.951, 1.051] | 0.782[0.682, 0.895] | 3.82e-04 | [0.447, 1.368] | 9.79e-03 | 82.867 | 0.00e+00 | Suggestive |
| Lifestyle Quality Indices | highest vs. lowest category | 17 | 43794/1170291 | [0.795, 1.217] | 0.772[0.711, 0.838] | 7.47e-10 | [0.595, 1.002] | 9.65e-02 | 67.781 | 2.57e-02 | Suggestive |
| Tofu intake | highest vs. lowest category | 14 | 17075/136740 | [0.708, 0.982] | 0.769[0.674, 0.878] | 9.66e-05 | [0.512, 1.157] | 2.85e-02 | 57.336 | 9.55e-02 | Suggestive |
| Isoflavone intake | highest vs. lowest category | 46 | 56276/1907714 | [0.91, 1.1] | 0.762[0.704, 0.824] | 1.16e-11 | [0.479, 1.212] | 9.47e-09 | 83.163 | 5.87e-08 | Suggestive |
| adherence score | highest vs. lowest category | 7 | 72420/1449105 | [0.626, 0.869] | 0.736[0.646, 0.839] | 4.33e-06 | [0.506, 1.07] | 8.89e-01 | 65.023 | 6.66e-01 | Suggestive |
| choline | highest vs. lowest category | 10 | 28120/664839 | [0.929, 1.079] | 0.688[0.565, 0.836] | 1.78e-04 | [0.347, 1.361] | 1.90e-03 | 91.857 | 1.58e-08 | Suggestive |
| Higher Mushroom Consumption | highest vs. lowest category | 9 | 17069/391275 | [0.85, 1.13] | 0.665[0.529, 0.835] | 4.62e-04 | [0.321, 1.375] | 6.14e-02 | 81.695 | 1.64e-02 | Suggestive |
| soy protein | highest vs. lowest category | 5 | 3034/78805 | [0.673, 1.172] | 0.646[0.507, 0.824] | 4.26e-04 | [0.32, 1.306] | 9.40e-03 | 44.742 | 1.67e-03 | Suggestive |
| ***Imageological diagnosis*** | | | | | | | | | | | |
| bone mineral density | highest vs. lowest category | 13 | 5511/222331 | [1.17, 1.463] | 1.707[1.351, 2.156] | 7.30e-06 | [0.732, 3.98] | 5.92e-01 | 83.23 | 3.34e-01 | Suggestive |
| ***Environment*** | | | | | | | | | | | |
| NO2 | per 10 ug/m3 | 17 | 257334/8922782 | [1.003, 1.017] | 1.023[1.011, 1.035] | 2.26e-04 | [0.987, 1.06] | 1.09e-02 | 56.369 | 2.99e-02 | Suggestive |
| PAHs | highest vs. lowest category | 16 | 23741/63740 | [1.353, 4.516] | 1.245[1.112, 1.395] | 1.55e-04 | [0.86, 1.804] | 3.29e-02 | 60.026 | 4.48e-05 | Suggestive |
| ***Life behaviour*** | | | | | | | | | | | |
| Negative Emotions | ever vs. never | 10 | 2080/129621 | [0.968, 1.078] | 1.902[1.313, 2.754] | 6.66e-04 | [0.605, 5.975] | 1.28e-03 | 79.723 | 1.41e-08 | Suggestive |
| passive smoking | ever vs. never | 69 | 359650/1670507 | [0.918, 1.086] | 1.475[1.377, 1.581] | 1.80e-28 | [0.942, 2.311] | 2.29e-13 | 86.202 | 0.00e+00 | Suggestive |
| smoking | ever vs. never | 88 | 140414/2423747 | [0.969, 1.055 | 1.352[1.251, 1.461] | 2.49e-14 | [0.764, 2.391] | 1.41e-03 | 96.131 | 5.66e-11 | Suggestive |
| famine exposure | ever vs. never | 8 | 12595/202489 | [0.818, 1.444] | 1.215[1.118, 1.321] | 4.78e-06 | [0.994, 1.486] | 5.87e-01 | 40.247 | 8.39e-01 | Suggestive |
| sedentary work | highest vs. lowest category | 30 | 111239/3382206 | [0.669, 1.487] | 1.127[1.055, 1.205] | 4.37e-04 | [0.876, 1.451] | 1.73e-01 | 48.891 | 1.34e-01 | Suggestive |
| night-shift work | ever vs. never | 48 | 142656/7381545 | [1.009, 1.179] | 1.075[1.03, 1.122] | 9.38e-04 | [0.867, 1.333] | 2.78e-02 | 73.124 | 4.59e-02 | Suggestive |
| Time in the Sun | highest vs. lowest category | 9 | 10406/158397 | [0.758, 0.958] | 0.838[0.768, 0.913] | 5.34e-05 | [0.637, 1.1] | 5.17e-02 | 73.37 | 4.44e-02 | Suggestive |
| ***Anthropometric indices*** | | | | | | | | | | | |
| fat mass | highest vs. lowest category | 7 | 5956/265841 | [1.43, 1.79] | 1.445[1.226, 1.703] | 1.16e-05 | [0.883, 2.364] | 8.94e-01 | 68.895 | 2.07e-02 | Suggestive |
| BMI | highest vs. lowest category | 43 | 58607/5069018 | [0.712, 0.972] | 1.25[1.151, 1.358] | 1.37e-07 | [0.801, 1.951] | 2.23e-01 | 77.084 | 3.16e-02 | Suggestive |
| BMI>=30 | ever vs. never | 45 | 99211/1709570 | [0.766, 1.017] | 1.222[1.118, 1.336] | 1.02e-05 | [0.762, 1.961] | 9.68e-01 | 83.065 | 2.16e-04 | Suggestive |
| height | per 10 cm | 14 | 9730/247706 | [0.981, 1.081] | 1.032[1.015, 1.049] | 2.27e-04 | [0.988, 1.077] | 6.07e-01 | 36.95 | 3.94e-01 | Suggestive |
| BMI | per 5 kg/m2 | 33 | 59213/4132649 | [0.997, 1.109] | 0.934[0.904, 0.966] | 5.78e-05 | [0.794, 1.098] | 2.00e-02 | 90.877 | 2.55e-09 | Suggestive |
| BMI<25 | high vs. low, postmenopausal | 31 | 50127/1314494 | [0.867, 1.047] | 0.788[0.72, 0.862] | 2.14e-07 | [0.538, 1.155] | 2.11e-03 | 62.88 | 3.08e-02 | Suggestive |
| ***Biomarkers*** | | | | | | | | | | | |
| Antibody | highest vs. lowest category | 8 | 1687/7727 | [1.104, 3.523] | 2.522[1.666, 3.816] | 1.21e-05 | [0.747, 8.517] | 2.50e-02 | 65.786 | 2.44e-06 | Suggestive |
| u Cadmium | highest vs. lowest category | 5 | 1416/6499 | [1.325, 4.916] | 2.226[1.445, 3.429] | 2.81e-04 | [0.513, 9.663] | 1.47e-01 | 70.557 | 4.62e-02 | Suggestive |
| IGF-1 | highest vs. lowest category | 19 | 6122/17874 | [1.065, 1.675] | 1.267[1.15, 1.397] | 1.87e-06 | [1.141, 1.407] | 3.93e-01 | 0 | 8.28e-01 | Suggestive |
| blood vitamin D | per 5 nmol/L | 40 | 24291/125215 | [1.003, 1.053] | 0.943[0.926, 0.96] | 1.12e-10 | [0.847, 1.05] | 8.35e-04 | 90.959 | 6.22e-03 | Suggestive |
| Adiponectin | highest vs. lowest category | 14 | 11051/24192 | [0.687, 0.975] | 0.749[0.636, 0.881] | 4.92e-04 | [0.452, 1.242] | 2.07e-02 | 58.993 | 5.42e-02 | Suggestive |
| blood vitamin D | highest vs. lowest category | 50 | 31316/165866 | [1.021, 1.361] | 0.555[0.468, 0.658] | 1.18e-11 | [0.183, 1.684] | 7.07e-05 | 93.105 | 3.78e-10 | Suggestive |
| ***Use of medical/hormonal therapy*** | | | | | | | | | | | |
| Oral Contraceptive>5years | ever vs. never | 4 | 6385/9326 | [0.814, 3.061] | 2.662[1.796, 3.944] | 1.08e-06 | [0.634, 11.174] | 2.70e-01 | 45.302 | 9.50e-01 | Suggestive |
| antibiotic use | ever vs. never | 8 | 31397/2240174 | [1.09, 1.17] | 1.179[1.077, 1.291] | 3.59e-04 | [0.887, 1.568] | 7.18e-02 | 90.674 | 7.78e-02 | Suggestive |
| Oral Contraceptive | ever vs. never | 70 | 65804/575605 | [0.89, 1.034] | 1.142[1.073, 1.215] | 2.68e-05 | [0.744, 1.751] | 6.84e-02 | 90.46 | 1.69e-11 | Suggestive |
| bisphosphonates | ever vs. never | 12 | 269153/2999920 | [0.885, 0.955] | 0.861[0.806, 0.92] | 9.75e-06 | [0.709, 1.046] | 2.59e-01 | 67.31 | 5.02e-03 | Suggestive |
| antipsychotic use | ever vs. never | 36 | 358692/5405662 | [1.119, 1.289] | 1.082[1.045, 1.119] | 6.58e-06 | [0.919, 1.273] | 3.33e-01 | 79.648 | 2.30e-02 | Suggestive |
| ***Pre-existing medical conditions and interventions*** | | | | | | | | | | | |
| obstructive sleep apnea | ever vs. never | 12 | 233375/7942160 | [0.928, 0.978] | 1.494[1.229, 1.817] | 5.69e-05 | [0.697, 3.204] | 6.06e-01 | 99.661 | 7.30e-02 | Suggestive |
| Periodontal disease | ever vs. never | 12 | 7424/342916 | [1.059, 1.201] | 1.235[1.108, 1.376] | 1.39e-04 | [0.954, 1.599] | 5.19e-02 | 52.106 | 2.84e-02 | Suggestive |
| Diabetes | ever vs. never | 69 | 263906/18281388 | [1.106, 1.15] | 1.198[1.099, 1.305] | 3.69e-05 | [0.629, 2.282] | 7.85e-02 | 97.848 | 9.85e-01 | Suggestive |
| Metabolic Syndrome | ever vs. never | 17 | 25569/1182195 | [0.904, 0.992] | 1.198[1.08, 1.328] | 6.25e-04 | [0.824, 1.74] | 1.55e-03 | 76.861 | 5.08e-09 | Suggestive |
| progestagen-releasing IUDs | ever vs. never | 5 | 7033/53369 | [0.488, 1.589] | 1.184[1.078, 1.3] | 4.23e-04 | [0.902, 1.554] | 2.38e-01 | 48.519 | 4.65e-01 | Suggestive |
| hyperthyroidism | ever vs. never | 24 | 160698/4676384 | [1.118, 1.358] | 1.122[1.066, 1.181] | 1.02e-05 | [0.976, 1.29] | 2.96e-01 | 44.095 | 1.97e-01 | Suggestive |
| hypertension | ever vs. never | 27 | 39693/459825 | [0.972, 1.152] | 1.112[1.046, 1.182] | 6.25e-04 | [0.872, 1.418] | 8.07e-01 | 67.971 | 4.21e-01 | Suggestive |
| bariatric surgery | ever vs. never | 16 | 28734/2534151 | [0.459, 0.606] | 0.583[0.462, 0.738] | 6.56e-06 | [0.218, 1.562] | 8.52e-01 | 96.591 | 8.73e-01 | Suggestive |
| ***Past gynaecological history*** | | | | | | | | | | | |
| age at marriage | highest vs. lowest category | 9 | 3147/6461 | [1.031, 1.661] | 2.172[1.472, 3.205] | 9.30e-05 | [0.579, 8.152] | 2.72e-02 | 85.712 | 1.79e-02 | Suggestive |
| marital status | ever vs. never | 63 | 28638/12657 | [0.812, 0.972] | 1.285 [1.151, 1.435] | 8.04e-06 | [0.649, 2.547] | 5.24e-03 | 82.302 | 2.16e-11 | Suggestive |
| abortion | ever vs. never | 35 | 14875/299619 | [0.8, 1.08] | 1.221[1.098, 1.357] | 2.30e-04 | [0.716, 2.082] | 1.66e-02 | 82.473 | 3.15e-04 | Suggestive |
| parity | ever vs. never | 81 | 107961/3051814 | [0.793, 1.191] | 0.891[0.838, 0.947] | 1.93e-04 | [0.597, 1.328] | 2.09e-01 | 78.682 | 0.00e+00 | Suggestive |
| **Weak evidence** | | | | | | | | | | | |
| ***Dietary intake*** | | | | | | | | | | | |
| unhealthy Dietary pattern | highest vs. lowest category | 9 | 4185/113894 | [1.028, 1.699] | 1.442[1.06, 1.961] | 1.98e-02 | [0.522, 3.984] | 5.37e-01 | 81.542 | 3.37e-02 | Weak |
| DII | per 1‐point | 6 | 15007/172704 | [1.008, 1.759] | 1.325[1.043, 1.683] | 2.14e-02 | [0.586, 2.996] | 3.43e-01 | 87.333 | 1.22e-02 | Weak |
| dietary cholesterol intake | highest vs. lowest category | 9 | 8668/356119 | [0.977, 1.157] | 1.287[1.061, 1.563] | 1.06e-02 | [0.734, 2.258] | 8.60e-02 | 71.078 | 1.70e-01 | Weak |
| drinker dietary pattern | highest vs. lowest category | 4 | 2645/66183 | [1.06, 1.52] | 1.217[1.048, 1.413] | 9.94e-03 | [0.811, 1.827] | 3.38e-01 | 12.033 | 9.77e-02 | Weak |
| processed meat intake | per 50 g/day | 17 | 92323/2522068 | [0.438, 1.219] | 1.178[1.04, 1.333] | 9.77e-03 | [0.788, 1.761] | 1.70e-01 | 63.227 | 7.91e-02 | Weak |
| SSBs | per 250mg/day | 7 | 14886/455697 | [0.968, 1.038] | 1.171[1.004, 1.365] | 4.38e-02 | [0.779, 1.76] | 3.66e-02 | 64.874 | 5.02e-01 | Weak |
| heme iron intake | highest vs. lowest category | 6 | 19471/394937 | [1.032, 1.191] | 1.124[1.038, 1.217] | 3.82e-03 | [0.921, 1.372] | 6.29e-01 | 38.601 | 4.96e-01 | Weak |
| Western/unhealthy dietary pattern | highest vs. lowest category | 15 | 20538/501844 | [0.709, 1.329] | 1.118[1.001, 1.248] | 4.74e-02 | [0.756, 1.653] | 1.20e-01 | 69.888 | 8.55e-02 | Weak |
| red meat intake | per 100 g/day | 20 | 118261/2689560 | [1.043, 2.211] | 1.104[1.032, 1.181] | 4.00e-03 | [0.885, 1.377] | 1.85e-03 | 60.181 | 5.64e-03 | Weak |
| processed meat intake | highest vs. lowest category | 20 | 60327/2350966 | [0.942, 1.122] | 1.08[1.026, 1.137] | 3.16e-03 | [0.906, 1.287] | 3.90e-01 | 59.682 | 2.03e-01 | Weak |
| dietary MUFA intake | highest vs. lowest category | 39 | 58203/2267808 | [0.88, 1.16] | 1.077[1.004, 1.156] | 3.87e-02 | [0.786, 1.477] | 6.20e-02 | 70.576 | 2.00e-02 | Weak |
| total meat intake | per 100 g/day | 14 | 54318/1273567 | [0.921, 1.041] | 1.068[1.012, 1.128] | 1.73e-02 | [0.908, 1.257] | 7.89e-03 | 75.531 | 3.10e-14 | Weak |
| Glycemic index | highest vs. lowest category | 23 | 89489/2940967 | [0.955, 1.087] | 1.051[1, 1.103] | 4.86e-02 | [0.879, 1.256] | 4.83e-01 | 62.287 | 5.55e-02 | Weak |
| Glycemic index/Glycemic load | highest vs. lowest category | 14 | 66280//1162453 | [0.988, 1.119] | 1.049[1.013, 1.086] | 7.10e-03 | [1.009, 1.09] | 5.75e-01 | 0 | 6.55e-01 | Weak |
| Glycemic index | per 10 units/day | 10 | 36826/1110003 | [1.008, 1.118] | 1.037[1.003, 1.072] | 3.37e-02 | [0.965, 1.115] | 3.87e-01 | 25.27 | 8.87e-02 | Weak |
| folate intake | per 100 ug/day | 27 | 42475/1093823 | [0.95, 1.03] | 0.984[0.974, 0.995] | 5.73e-03 | [0.942, 1.029] | 3.70e-03 | 72.158 | 1.19e-01 | Weak |
| vegetable intake | per 100 g/day | 14 | 76875/1550521 | [0.91, 0.99] | 0.974[0.952, 0.996] | 1.87e-02 | [0.909, 1.043] | 1.49e-03 | 58.911 | 5.98e-04 | Weak |
| soy intake | per 30 g/day | 7 | 4122/155528 | [0.691, 1.171] | 0.967[0.94, 0.994] | 1.63e-02 | [0.932, 1.002] | 5.90e-01 | 0 | 4.97e-01 | Weak |
| soy isoflavone | per 10mg/day | 9 | 10229/631498 | [0.896, 1.057] | 0.967[0.945, 0.99] | 5.18e-03 | [0.926, 1.01] | 6.49e-01 | 15.368 | 4.32e-01 | Weak |
| fruits and vegetables intake | per 200 g/day | 6 | 6220/226880 | [0.894, 1.044] | 0.966[0.933, 1] | 4.77e-02 | [0.92, 1.014] | 3.16e-01 | 0 | 8.22e-01 | Weak |
| coffee intake | highest vs. lowest category | 29 | 115874/3019649 | [0.917, 1.019] | 0.957[0.922, 0.994] | 2.28e-02 | [0.854, 1.073] | 8.84e-01 | 35.01 | 3.79e-01 | Weak |
| methionine | highest vs. lowest category | 16 | 32200/780908 | [0.912, 1.071] | 0.953[0.91, 0.998] | 4.12e-02 | [0.906, 1.002] | 2.36e-01 | 0 | 1.97e-01 | Weak |
| cheese intake | per 30 g/day | 10 | 46744/1419872 | [0.948, 1.018] | 0.952[0.909, 0.996] | 3.30e-02 | [0.839, 1.079] | 2.19e-01 | 75.285 | 3.81e-01 | Weak |
| dietary b-carotene | per 5000ug/day | 10 | 18191/825911 | [0.908, 1.018] | 0.95[0.914, 0.988] | 9.55e-03 | [0.908, 0.994] | 6.50e-01 | 0 | 5.92e-01 | Weak |
| calcium intake | per 350mg/day | 6 | 86605/2761892 | [0.976, 0.992] | 0.934[0.883, 0.987] | 1.57e-02 | [0.807, 1.081] | 1.32e-02 | 70.289 | 9.83e-07 | Weak |
| dietary folate intake | highest vs. lowest category | 24 | 42596/1192483 | [0.832, 1.012] | 0.929[0.875, 0.987] | 1.63e-02 | [0.747, 1.155] | 7.71e-02 | 56.336 | 4.65e-01 | Weak |
| vitamin B2 | highest vs. lowest category | 10 | 20100/622953 | [0.882, 1.042] | 0.899[0.819, 0.986] | 2.46e-02 | [0.712, 1.134] | 1.71e-01 | 42.502 | 7.09e-01 | Weak |
| prudent/healthy dietary pattern | highest vs. lowest category | 16 | 23107/507826 | [0.68, 1.19] | 0.899[0.815, 0.992] | 3.43e-02 | [0.622, 1.299] | 8.93e-01 | 74.003 | 2.29e-01 | Weak |
| fruits and vegetables intake | highest vs. lowest category | 6 | 5206/188188 | [0.76, 1.09] | 0.890[0.802, 0.989] | 3.01e-02 | [0.768, 1.033] | 5.85e-01 | 0 | 8.39e-01 | Weak |
| tea consumption | highest vs. lowest category | 46 | 54984/1693694 | [0.667, 0.988] | 0.886[0.819, 0.959] | 2.73e-03 | [0.56, 1.401] | 8.52e-01 | 83.907 | 6.87e-01 | Weak |
| calcium intake | highest vs. lowest category | 7 | 286693/766589 | [0.929, 1.03] | 0.88[0.785, 0.987] | 2.92e-02 | [0.648, 1.196] | 1.91e-02 | 63.764 | 1.07e-02 | Weak |
| whole grain intake | highest vs. lowest category | 15 | 18442/342107 | [0.834, 0.993] | 0.878[0.801, 0.961] | 4.95e-03 | [0.667, 1.154] | 4.23e-03 | 70.591 | 2.98e-13 | Weak |
| a-carotene | highest vs. lowest category | 16 | 23668/390569 | [0.67, 1.08] | 0.877[0.805, 0.954] | 2.43e-03 | [0.676, 1.137] | 1.47e-01 | 50.083 | 3.86e-01 | Weak |
| betaine | highest vs. lowest category | 10 | 31120/664839 | [0.892, 1.052] | 0.863[0.769, 0.968] | 1.16e-02 | [0.612, 1.216] | 3.65e-02 | 72.704 | 2.21e-01 | Weak |
| Selenium | highest vs. lowest category | 14 | 8398/144625 | [0.519, 0.919] | 0.858[0.768, 0.958] | 6.59e-03 | [0.664, 1.108] | 2.80e-01 | 31.478 | 2.71e-01 | Weak |
| total dairy food intake | highest vs. lowest category | 12 | 19339/662579 | [0.882, 1.042] | 0.854[0.765, 0.952] | 4.53e-03 | [0.631, 1.155] | 2.66e-02 | 54.574 | 2.65e-03 | Weak |
| vegetarians | yes vs. no | 4 | 471/558626 | [0.539, 0.99] | 0.849[0.722, 0.998] | 4.68e-02 | [0.503, 1.431] | 8.40e-01 | 29.043 | 3.84e-02 | Weak |
| Flavones | highest vs. lowest category | 9 | 12073/437523 | [0.912, 1.071] | 0.843[0.734, 0.968] | 1.53e-02 | [0.539, 1.318] | 2.41e-01 | 85.487 | 8.52e-02 | Weak |
| dietary vitamin A | highest vs. lowest category | 19 | 26775/1088168 | [0.831, 1.201] | 0.841[0.757, 0.935] | 1.36e-03 | [0.586, 1.207] | 1.52e-01 | 61.211 | 2.10e-01 | Weak |
| Flavonols | highest vs. lowest category | 11 | 13135/529214 | [0.884, 1.034] | 0.837[0.747, 0.939] | 2.41e-03 | [0.582, 1.205] | 1.06e-01 | 76.161 | 5.25e-01 | Weak |
| dietary carrot intake | highest vs. lowest category | 10 | 16535/163605 | [0.667, 1.038] | 0.809[0.709, 0.923] | 1.59e-03 | [0.545, 1.202] | 1.96e-02 | 71.511 | 3.64e-02 | Weak |
| healthy Dietary pattern | highest vs. lowest category | 10 | 4285114168 | [0.75, 1.23] | 0.622[0.441, 0.879] | 7.10e-03 | [0.19, 2.036] | 8.20e-02 | 88.16 | 5.24e-01 | Weak |
| Healthy eating index | highest vs. lowest category | 4 | 1024/2688 | [0.611, 1.18] | 0.485[0.271, 0.867] | 1.46e-02 | [0.042, 5.581] | 3.77e-02 | 71.613 | 2.53e-01 | Weak |
| ***Life behaviour*** | | | | | | | | | | | |
| striking life events | ever vs. never | 7 | 3326/99870 | [1.01, 1.24] | 1.505[1.152, 1.965] | 2.69e-03 | [0.65, 3.483] | 1.53e-01 | 92.545 | 1.15e-06 | Weak |
| Occupation | ever vs. never | 18 | 3926/12203 | [0.448, 1.259] | 1437[1.095, 1.885] | 8.89e-03 | [0.484, 4.267] | 1.80e-01 | 81.879 | 3.41e-02 | Weak |
| flight attendants | ever vs. never | 4 | 680/22410 | [1.324, 1.693] | 1.425[1.321, 1.537] | 4.31e-20 | [1.207, 1.683] | 5.77e-01 | 0 | 5.19e-01 | Weak |
| Radial scars | ever vs. never | 5 | 2521/22801 | [1.068, 1.789] | 1.355[1.039, 1.766] | 2.47e-02 | [0.543, 3.378] | 4.38e-01 | 79.796 | 4.22e-05 | Weak |
| place of residence | ever vs. never | 9 | 5488/13465 | [1.223, 1.552] | 1.264[1.075, 1.486] | 4.57e-03 | [0.776, 2.06] | 7.41e-01 | 71.406 | 1.46e-01 | Weak |
| sedentary behavior | per 1h/d | 8 | 17048/462782 | [1, 1.02] | 1.011[1, 1.023] | 4.73e-02 | [0.98, 1.044] | 5.12e-01 | 65.488 | 2.31e-01 | Weak |
| ***Environment*** | | | | | | | | | | | |
| Cadmium | ever vs. never | 17 | 24017/399191 | [0.81, 1] | 1.163[1.021, 1.325] | 2.31e-02 | [0.719, 1.881] | 7.85e-02 | 77.236 | 2.52e-02 | Weak |
| ***Virus infection*** | | | | | | | | | | | |
| BLV | ever vs. never | 8 | 826/1724 | [0.691, 1.486] | 2.902[1.548, 5.442] | 8.92e-04 | [0.346, 24.326] | 1.14e-01 | 84.988 | 5.18e-03 | Weak |
| ***Biomarkers*** | | | | | | | | | | | |
| IGFBP-3 | highest vs. lowest category | 5 | 951/2687 | [0.732, 1.766] | 1.468[1.085, 1.986] | 1.27e-02 | [0.81, 2.66] | 4.29e-01 | 8.914 | 5.38e-01 | Weak |
| circulating resistin levels | highest vs. lowest category | 8 | 1803/3902 | [0.813, 1.222] | 1.267[1.064, 1.508] | 7.84e-03 | [0.83, 1.934] | 1.46e-01 | 44.106 | 1.53e-03 | Weak |
| serum/plasma iron | highest vs. lowest category | 4 | 4858/379833 | [0.993, 1.273] | 1.258[1.016, 1.559] | 3.56e-02 | [0.544, 2.912] | 4.11e-01 | 58.909 | 6.26e-01 | Weak |
| plasma prolactin levels | highest vs. lowest category | 7 | 6388/12380 | [0.99, 1.29] | 1.16[1.017, 1.323] | 2.72e-02 | [0.927, 1.452] | 5.82e-01 | 7.592 | 5.39e-01 | Weak |
| CRP | highest vs. lowest category | 15 | 11961/292728 | [0.9, 1.201] | 1.146[1.035, 1.269] | 8.71e-03 | [0.863, 1.523] | 2.69e-02 | 41.813 | 3.56e-01 | Weak |
| resistin | per 1 ng/ml | 8 | 1505/2885 | [0.958, 1.028] | 1.089[1.008, 1.176] | 3.01e-02 | [0.878, 1.351] | 1.61e-02 | 82.78 | 2.31e-02 | Weak |
| HDL–C | highest vs. lowest category | 20 | 23022/839656 | [0.911, 1.214] | 0.889[0.801, 0.987] | 2.78e-02 | [0.617, 1.282] | 3.07e-01 | 64.657 | 1.84e-01 | Weak |
| adiponectin | per 5 µg/ml | 14 | 3735/7704 | [0.905, 1.006] | 0.846[0.753, 0.951] | 5.07e-03 | [0.594, 1.207] | 6.67e-03 | 81.869 | 1.05e-02 | Weak |
| lignans and enterolignans | highest vs. lowest category | 21 | 17897/166348 | [0.794, 1.112] | 0.849[0.751, 0.96] | 9.17e-03 | [0.532, 1.356] | 1.06e-01 | 66.731 | 3.23e-06 | Weak |
| serum PLP levels | highest vs. lowest category | 5 | 2442/4885 | [0.633, 1.302] | 0.803[0.66, 0.977] | 2.84e-02 | [0.579, 1.112] | 1.01e-01 | 0.827 | 4.18e-01 | Weak |
| a-carotene in blood | per 10 µg/dL | 10 | 3840/14549 | [0.542, 1.361] | 0.820[0.725, 0.928] | 1.70e-03 | [0.676, 0.995] | 2.78e-01 | 7.296 | 4.88e-01 | Weak |
| b-carotene in blood | per 50 µg/dL | 11 | 3879/14666 | [0.402, 1.431] | 0.751[0.582, 0.968] | 2.69e-02 | [0.391, 1.442] | 9.46e-01 | 42.704 | 3.24e-01 | Weak |
| total carotenoids in blood | per 100 µg/dL | 7 | 2837/12245 | [0.583, 1.012] | 0.791[0.648, 0.966] | 2.17e-02 | [0.452, 1.386] | 8.74e-01 | 53.559 | 5.66e-01 | Weak |
| lutein in blood | per 25 µg/dL | 4 | 1514/4116 | [0.387, 1.01] | 0.681[0.519, 0.894] | 5.67e-03 | [0.375, 1.238] | 5.98e-01 | 0 | 8.93e-01 | Weak |
| ***Past gynaecological history*** | | | | | | | | | | | |
| menopausal status | ever vs. never | 24 | 5300/49345 | [0.708, 2.139] | 1.363[1.095, 1.696] | 5.63e-03 | [0.483, 3.842] | 2.45e-04 | 91.938 | 1.05e-02 | Weak |
| age at last birth (ALB) | ever vs. never | 14 | 130608/1315601 | [1.079, 1.269] | 1.22[1.045, 1.423] | 1.18e-02 | [0.754, 1.973] | 2.60e-01 | 65.866 | 8.16e-02 | Weak |
| paternal age,dose-response | ever vs. never | 7 | 19181/157580 | [1.007, 1.228] | 1.131[1.021, 1.254] | 1.85e-02 | [0.884, 1.448] | 6.58e-01 | 40.772 | 6.16e-02 | Weak |
| at-term pregnancies | ever vs. never | 4 | 554/2851 | [0.6, 1.04] | 0.803[0.66, 0.977] | 2.83e-02 | [0.523, 1.235] | 3.55e-01 | 0 | 8.35e-01 | Weak |
| ***Use of medical/hormonal therapy*** | | | | | | | | | | | |
| Postmenopausal hormone therapy | ever vs. never | 7 | 2555/442632 | [0.892, 1.072] | 1.245[1.009, 1.536] | 4.07e-02 | [0.667, 2.327] | 8.52e-02 | 69.624 | 6.13e-01 | Weak |
| Calcium channel blockers | ever vs. never | 11 | 29150/292038 | [1.21, 1.42] | 1.136[1.021, 1.264] | 1.89e-02 | [0.806, 1.603] | 1.56e-01 | 85.716 | 8.58e-01 | Weak |
| Beta Blockers | ever vs. never | 17 | 23378/391158 | [0.894, 1.026] | 1.12[1.01, 1.242] | 3.24e-02 | [0.766, 1.638] | 7.50e-01 | 86.703 | 7.97e-01 | Weak |
| Thiazolidinediones | ever vs. never | 12 | 57014/2678400 | [0.787, 0.968] | 0.88[0.786, 0.986] | 2.71e-02 | [0.649, 1.193] | 2.29e-01 | 57.398 | 3.15e-01 | Weak |
| insulins | ever vs. never | 12 | 22890/6239843 | [0.892, 1.052] | 0.873[0.781, 0.975] | 1.56e-02 | [0.621, 1.226] | 9.71e-02 | 78.044 | 3.13e-01 | Weak |
| incretin-based medicines | ever vs. never | 2 | 2453/1133605 | [0.561, 0.871] | 0.777[0.608, 0.994] | 4.44e-02 | < 3 studies | < 3 studies | 44.299 | NA | Weak |
| ***Pre-existing medical conditions and interventions*** | | | | | | | | | | | |
| serum 25(OH)D deficient | ever vs. never | 5 | 1306/2796 | [0.649, 1.48] | 2.472[1.334, 4.579] | 4.01e-03 | [0.276, 22.171] | 1.80e-01 | 82.542 | 6.15e-03 | Weak |
| Goiter | ever vs. never | 6 | 8960/17634 | [0.761, 1.311] | 1.903[1.133, 3.196] | 1.50e-02 | [0.32, 11.301] | 5.33e-01 | 86.331 | 1.29e-01 | Weak |
| Sleep-disordered breathing | ever vs. never | 8 | 8300/1398113 | [0.93, 0.99] | 1.357[1.077, 1.709] | 9.68e-03 | [0.615, 2.994] | 3.61e-02 | 94.576 | 1.96e-08 | Weak |
| tonsils removed | ever vs. never | 8 | 2671/7566 | [0.811, 5.03] | 1.246[1.06, 1.465] | 7.62e-03 | [0.865, 1.797] | 7.92e-01 | 32.54 | 5.07e-01 | Weak |
| Schizophrenia | ever vs. never | 15 | 54228/3408696 | [0.715, 1.123] | 1.174[1.042, 1.322] | 8.20e-03 | [0.738, 1.869] | 3.36e-01 | 89.055 | 2.68e-01 | Weak |
| hypothyroidism | ever vs. never | 18 | 112031/1150664 | [0.939, 1.046] | 0.91[0.843, 0.981] | 1.45e-02 | [0.699, 1.184] | 5.09e-01 | 81.681 | 1.55e-02 | Weak |
| CAD | ever vs. never | 5 | 16527/1109286 | [0.641, 0.9] | 0.87[0.776, 0.975] | 1.65e-02 | [0.596, 1.269] | 1.05e-01 | 66.937 | 3.26e-02 | Weak |
| Migraine | ever vs. never | 9 | 73354/397984 | [0.886, 1.207] | 0.821[0.721, 0.936] | 3.09e-03 | [0.52, 1.297] | 1.41e-01 | 88.745 | 2.58e-01 | Weak |
| ***Anthropometric indices*** | | | | | | | | | | | |
| birth length | highest vs. lowest category | 7 | 3093/15236 | [0.821, 1.341] | 1.312[1.098, 1.568] | 2.76e-03 | [0.904, 1.905] | 1.06e-01 | 22.862 | 7.44e-01 | Weak |
| BMI:20-25 | ever vs. never | 15 | 7593/22362 | [0.949, 1.23] | 1.187[1.064, 1.325] | 2.13e-03 | [0.863, 1.634] | 9.58e-01 | 49.545 | 4.22e-01 | Weak |
| birth weight | highest vs. lowest category | 8 | 9084/3352606 | [1.023, 1.332] | 1.184[1.058, 1.325] | 3.26e-03 | [1.029, 1.362] | 1.43e-01 | 0 | 6.10e-01 | Weak |
| waist-to-hip ratio | per 0.1 | 12 | 8344/149996 | [0.929, 1.099] | 1.093[1.035, 1.155] | 1.35e-03 | [0.905, 1.322] | 7.42e-01 | 77.955 | 8.38e-01 | Weak |
| birth weight | per 1 kg | 16 | 20480/3760273 | [1.01, 1.2] | 1.066[1.017, 1.118] | 7.77e-03 | [0.946, 1.202] | 3.22e-01 | 32.736 | 1.54e-01 | Weak |
| weight loss | highest vs. lowest category | 16 | 19351/1035071 | [0.63, 1.114] | 0.896[0.834, 0.963] | 2.68e-03 | [0.828, 0.969] | 2.89e-01 | 0 | 4.32e-01 | Weak |
| ***Imageological diagnosis*** | | | | | | | | | | | |
| High BPE | highest vs. lowest category | 9 | 495/1820 | [0.417, 1.699] | 3.582[1.516, 8.464] | 3.63e-03 | [0.186, 68.867] | 6.70e-02 | 88.852 | 3.31e-01 | Weak |
| Moderate BPE | highest vs. lowest category | 8 | 1035/6033 | [0.987, 2.969] | 2.883[1.515, 5.488] | 1.26e-03 | [0.367, 22.656] | 5.34e-02 | 78.332 | 3.54e-01 | Weak |
| ELF-EMFs | highest vs. lowest category | 27 | 119715/500898 | [1.118, 1.652] | 1.067[1.018, 1.118] | 6.62e-03 | [0.906, 1.256] | 3.14e-01 | 53.662 | 1.45e-01 | Weak |

**Key:**

*only meta-analyses meeting at least weak grade of evidence listed

**^*^** Number of studies

^#^ 95% confidence interval of largest study (smallest standard error) in each meta-analysis

**^¥^** Random effects refer to summary risk ratio (95% confidence interval) using the random-effects model

^||^ P value of summary random effects estimate

^∞^ P-value from the Egger’s regression asymmetry test

^α^ P value of the excess statistical significance test

All statistical tests were two-sided

Small study effect is based on the P-value from the Egger’s regression asymmetry test (P>0.1) where the random effects summary estimate was larger compared to the point estimate of the largest study in a meta-analysis

**Supplemental Table 8.** Evaluation of overlapping associations between included articles in the umbrella review according to the same exposure and outcome.

**（1）**

|  | *Wang et al.,2023** | *Ishihara et al., 2020* |
| --- | --- | --- |
| Primary studies |  |  |
| Christou., et al., 2008 | X | X |
| Mackenzie., et al., 2018 | X | X |
| Lazzati., et al., 2022 | X |  |
| Tao., et al., 2020 | X |  |
| Doumouras., et al., 2022 | X |  |
| Aravaini., et al., 2018 | X | X |
| Hassinger., et al., 2019 | X |  |
| Fergelson., et al., 2020 | X |  |
| Aminian., et al., 2022 | X |  |
| Anveden., et al., 2017 | X | X |
| Khalid., et al., 2021 | X |  |
| Tsui., et al., 2021 | X |  |
| Adams., et al., 2009 | X | X |
| McCawley., et al., 2009 |  | X |
| N | 19 | |
| Rows (r) | 14 | |
| Columns (c) | 2 | |
| Corrected Covered Area= (N-r)/rc-r | 35.7% very high CCA. * This article was selected due to the higher quality according to AMSTAR-2 tool. | |

**（2）**

|  | *Han, M. et al.,2022** | *Chen, S. et al., 2021* |
| --- | --- | --- |
| Primary studies |  |  |
| Kalache., et al., 1982 | X |  |
| Brinton., et al., 1984 | X | X |
| Smyth, et al., 1996 | X |  |
| Talamini., et al.,1997 | X | X |
| Simon., et al., 2002 | X |  |
| Turken., et al., 2003 | X |  |
| Freitas., et al., 2003 | X |  |
| Weng., et al., 2018 | X | X |
| Bach., et al., 2020 | X |  |
| Giani., et al., 1996 | X |  |
| Szychta., et al., 2013 | X |  |
| Moseson, et al., 1993 |  | X |
| Chen., et al., 2013 |  | X |
| Søgaard., et al., 2016 |  | X |
| N | 17 | |
| Rows (r) | 14 | |
| Columns (c) | 2 | |
| Corrected Covered Area= (N-r)/rc-r | 21.4% very high CCA. *This article was selected due to the higher quality according to AMSTAR-2 tool. | |

**（3）**

|  | *Pedro, M. et al.,2015** | *González-Pérez. et al., 2003* |
| --- | --- | --- |
| Primary studies |  |  |
| Rosenberg., et al., 1995 | X | X |
| Harris ., et al., 1995 | X | X |
| Harris., et al., 1996 | X | X |
| Neugut., et al.,1998 | X |  |
| Coogan., et al., 1999 | X | X |
| Langman., et al., 2000 | X | X |
| Cotterchio., et al., 2001 | X | X |
| Meier., et al., 2002 | X | X |
| Moorman., et al., 2003 | X |  |
| Terry., et al., 2004 | X |  |
| Rahme., et al., 2005 | X |  |
| Zhang., et al., 2005 | X |  |
| Swde., et al.,2005 | X |  |
| Harris., et al.,2006 | X |  |
| Kirsh., et al.,2007 | X |  |
| DavisyMirick, et al.,2007 | X |  |
| Slattery., et al.,2007 | X |  |
| Cronin-Fenton., et al.,2010 | X |  |
| Brasky., et al.,2010 | X |  |
| Ashok., et al.,2011 | X |  |
| Sharpe., et al.,2000 |  | X |
| Harris., et al., 1999 |  | X |
| N | 29 | |
| Rows (r) | 22 | |
| Columns (c) | 2 | |
| Corrected Covered Area= (N-r)/rc-r | 31.8 % very high CCA. *This article was selected due to the higher quality according to AMSTAR-2 tool. | |

**（4）**

|  | *Wu. et al.,2023* | *Yap. et al., 2022** |
| --- | --- | --- |
| Primary studies |  |  |
| Brenner., et al., 2018 | X |  |
| Campos-Rodriguez., et al.,2013 | X |  |
| Chang., et al., 2014 | X | X |
| Choi., et al.,2019 | X | X |
| Gozal., et al., 2019 | X |  |
| Jara., et al.,2020 | X | X |
| Justeau., et al., 2020 | X |  |
| Kendzerska., et al.,2014 | X |  |
| Sillah., et al.,2018 | X | X |
| Sillah., et al., 2019 | X |  |
| Gozal., et al., 2016 |  | X |
| Huang., et al., 2021 |  | X |
| N | 16 | |
| Rows (r) | 12 | |
| Columns (c) | 2 | |
| Corrected Covered Area= (N-r)/rc-r | 33.3% very high CCA. *This article was selected due to the higher quality according to AMSTAR-2 tool. | |

**（5）**

|  | *Santucci. et al.,2021* | *Qiao. et al., 2018** |
| --- | --- | --- |
| Primary studies |  |  |
| Paganini-Hill., et al., 1989 | X | X |
| Thun., et al.,1993 | X |  |
| Schreinemachers., et al., 1994 | X | X |
| Harris., et al.,1999 | X | X |
| Marshall., et al., 2005 | X | X |
| Gallicchio., et al.,2007 | X | X |
| Gill., et al., 2007 | X | X |
| Jacobs., et al.,2007 | X | X |
| Friis., et al.,2008 | X | X |
| Gierach., et al., 2008 | X | X |
| Ready., et al., 2008 | X | X |
| Siemes., et al., 2008 | X | X |
| Eliassen., et al.,2009 | X | X |
| Bardia., et al.,2011 | X | X |
| Bosco., et al.,2011 | X | X |
| Brasky., et al.,2014 | X | X |
| Cao., et al.,2016 | X | X |
| Clarke., et al.,2017 | X |  |
| Tsoi., et al.,2018 | X |  |
| Rodríguez., et al.,2004 | X | X |
| Rahme., et al.,2005 | X | X |
| Cronin-Fenton., et al.,2010 | X | X |
| Harris., et al.,1996 | X |  |
| Neugut., et al.,1998 | X | X |
| Cotterchio., et al.,2001 | X | X |
| Moorman., et al.,2003 | X | X |
| Terry., et al.,2004 | X | X |
| Swede., et al.,2005 | X | X |
| Zhang., et al.,2005 | X | X |
| Harris., et al.,2006 | X | X |
| Slattery., et al.,2007 | X | X |
| Brasky., et al.,2010 | X | X |
| Dierssen-Sotos., et al.,2016 | X | X |
| Iqbal., et al.,2017 |  | X |
| Cui., et al.,2014 |  | X |
| Slattery., et al.,2007 |  | X |
| Kim., et al.,2015 |  | X |
| Hollestein., et al.,2014 |  | X |
| N | 66 | |
| Rows (r) | 38 | |
| Columns (c) | 2 | |
| Corrected Covered Area= (N-r)/rc-r | 73.6% very high CCA. *This article was selected due to the higher quality according to AMSTAR-2 tool. | |

**（6）**

|  | *Peng. et al.,2020** | *Li. et al., 2020* |
| --- | --- | --- |
| Primary studies |  |  |
| Bae., et al., 2019 |  | X |
| Cardwell., et al.,2011 | X | X |
| Chiang., et al., 2012 | X | X |
| Chlebowski., et al.,2010 | X | X |
| Fournier., et al.,2017 | X | X |
| Hue., et al.,2014 | X |  |
| Lee., et al., 2012 | X | X |
| Monsees., et al.,2011 | X |  |
| Newcomb., et al.,2010 | X | X |
| Rennert., et al., 2010 | X | X |
| Vestergaard., et al., 2011 | X | X |
| Vinogradova., et al., 2013 | X | X |
| N | 21 | |
| Rows (r) | 12 | |
| Columns (c) | 2 | |
| Corrected Covered Area= (N-r)/rc-r | 42.9% very high CCA. *This article was selected due to the higher quality according to AMSTAR-2 tool. | |

**（7）**

|  | *Li. et al.,2020** | *Vishwakarma. et al., 2019** |
| --- | --- | --- |
| Primary studies |  |  |
| Adami., et al., 1978 | X |  |
| Ewertz., et al.,1986 | X |  |
| Gajalakshmi., et al., 1991 | X | X |
| Laing., et al.,1993 | X |  |
| Kvikstad., et al., 1994 | X |  |
| Rao., et al.,1994 |  | X |
| Rookus., et al., 1994 | X |  |
| White., et al.,1994 | X |  |
| Budiningsih., et al.,1999 | X |  |
| Wakai., et al., 2000 | X |  |
| Price., et al., 2001 | X |  |
| Li., et al., 2003 | X |  |
| Gilani and Kamal., et al.,2004 | X |  |
| Oran., et al.,2004 | X |  |
| Faheem., et al.,2007 | X |  |
| Mahouri., et al.,2007 | X |  |
| Samson., et al.,2007 |  | X |
| Lotfi and Shobairi., et al.,2008 | X |  |
| Peled., et al.,2008 | X |  |
| Datta., et al.,2009 |  | X |
| Dey., et al.,2009 | X | X |
| Gajalakshmi., et al.,2009 |  | X |
| Jayalekshmi., et al.,2009 |  | X |
| Marzouk., et al.,2009 | X |  |
| Pakseresht., et al.,2009 | X |  |
| Cho., et al.,2010 | X |  |
| Ghiasvand., et al.,2010 | X |  |
| Justenhoven., et al.,2010 | X |  |
| Tehranian., et al.,2010 | X |  |
| Eaker., et al.,2011 | X |  |
| Lodha., et al.,2011 |  | X |
| Motie., et al.,2011 | X |  |
| Parameshwari., et al.,2013 | X | X |
| Shamsi., et al.,2013 | X |  |
| Thompson., et al.,2013 | X |  |
| Pimhanam., et al.,2014 | X |  |
| Tazhibi., et al.,2014 | X |  |
| Wirth., et al.,2014 |  | X |
| Mohite., et al.,2015 | X | X |
| Sufian., et al.,2015 | X |  |
| Bano., et al.,2016 | X |  |
| Jafari-Mehdiabad., et al.,2016 | X |  |
| Yan., et al.,2016 | X |  |
| Balekouzou., et al.,2017 | X |  |
| Dianatinasab., et al.,2017 | X |  |
| Shaukat., et al.,2017 | X |  |
| Khalis., et al.,2018 | X |  |
| Khan., et al.,2018 | X | X |
| N | 52 | |
| Rows (r) | 48 | |
| Columns (c) | 2 | |
| Corrected Covered Area= (N-r)/rc-r | 8.3% moderate CCA. *Both articles were included | |

**（8）-menarcheal age**

|  | *Manouchehri. et al.,2022** | *Khoramdad. et al., 2022** |
| --- | --- | --- |
| Primary studies |  |  |
| Dianatinasab., et al., 2017 |  | X |
| Ghiasvand., et al.,2011 | X |  |
| Hajian., et al., 2012 |  | X |
| Hajian., et al.,2011 | X |  |
| Hosseinzade., et al.2014, |  | X |
| Jokar., et al.,2016 | X |  |
| Marzbani., et al., 2017 | X |  |
| Razmara., et al.,2010 | X |  |
| Sepandi., et al.,2014 | X |  |
| Tehranian., et al., 2010 | X | X |
| N | 11 | |
| Rows (r) | 10 | |
| Columns (c) | 2 | |
| Corrected Covered Area= (N-r)/rc-r | 10% moderate CCA. *Both articles were included | |

**（8）-menopausal status**

|  | *Manouchehri. et al.,2022** | *Khoramdad. et al., 2022** |
| --- | --- | --- |
| Primary studies |  |  |
| Lotfi.,et al., 2008 |  | X |
| Bahadoran., et al.,2013 |  | X |
| Dianatinasab., et al., 2017 | X | X |
| Ebrahimi., et al.,2002 | X |  |
| Ghosn., et al., 2020 |  | X |
| Hajian., et al.,2012 |  | X |
| Hajian., et al., 2011 | X |  |
| Heidari., et al.,2018 |  | X |
| Holakouie Naieni., et al.,2007 | X |  |
| Hosseinzade., et al., 2014 | X | X |
| Jokar., et al., 2016 | X |  |
| Keihanian., et al., 2010 | X |  |
| Lotfi., et al.,2008 | X |  |
| Mahouri., et al.,2007 | X |  |
| Montazeri., et al.,2004 | X |  |
| Mousazade., et al.,2019 | X |  |
| Pourzand., et al.,2016 |  | X |
| Rezaeiian., et al.,2012 |  | X |
| Safabakhsh., et al.,2020 |  | X |
| Sepandi., et al.,2014 | X |  |
| Sheikhi., et al.,2014 |  | X |
| Vahid., et al.,2018 |  | X |
| Yavari., et al.,2005 | X |  |
| Zare., et al.,2013 | X |  |
| Zayeri., et al.,2016 | X |  |
| N | 27 | |
| Rows (r) | 25 | |
| Columns (c) | 2 | |
| Corrected Covered Area= (N-r)/rc-r | 8% moderate CCA. *Both articles were included | |

**（9）**

|  | *Li. et al.,2021* | *Lafranconi. et al., 2018** |
| --- | --- | --- |
| Primary studies |  |  |
| Arthur., et al., 2018 | X |  |
| Baker., et al.,2006 | X |  |
| Bhoo-Pathy., et al.2015, | X | X |
| Bissonauth., et al.,2009 | X |  |
| Boggs., et al., 2010 | X | X |
| Folsom., et al.,1993 |  | X |
| Ganmaa., et al., 2008 | X | X |
| Gierach., et al.,2012 | X | X |
| Gronwald., et al.,2006 | X |  |
| Harris., et al., 2015 | X |  |
| Hashibe., et al., 2015 | X | X |
| Hirose., et al., 2007 | X |  |
| Hirvonen., et al.,2006 | X | X |
| Hoyer., et al.,1992 |  | X |
| Ishitani., et al.,2008 | X | X |
| Key., et al.,1999 |  | X |
| Kotemori., et al.,2017 | X |  |
| Kotsopoulos., et al.,2007 | X |  |
| Larsson., et al.,2009 | X | X |
| Lee., et al.,2019 | X |  |
| Li., et al.,2011 | X |  |
| Mizoo., et al.,2013 | X |  |
| Nilsson., et al.,2010 | X |  |
| Nkondjock., et al.,2006 | X |  |
| Oh., et al.,2015 | X | X |
| Suzuki., et al.,2004 |  | X |
| Vatten., et al.,1990 |  | X |
| Wilson., et al.,2009 | X | X |
| Yaghjyan., et al., 2018 | X |  |
| N | 39 | |
| Rows (r) | 29 | |
| Columns (c) | 2 | |
| Corrected Covered Area= (N-r)/rc-r | 34.5% very high CCA. *This article was selected due to the higher quality according to AMSTAR-2 tool. | |

**(10)**

|  | *Turner. et al.,2011* | *Boyd. et al., 2003** |
| --- | --- | --- |
| Primary studies |  |  |
| Bingham., et al.,2003 | X | X |
| Byrne., et al.,2002 | X |  |
| Chaillier., et al., 1998 | X | X |
| Cho., et al.,2003 | X | X |
| Destefani., et al.1998, | X | X |
| Ewertz., et al.,1990 | X | X |
| Franceschi., et al., 1996 |  | X |
| Gaard., et al.,1995 | X | X |
| Gago-Dominguez., et al.,2003 | X |  |
| Goodstine., et al.,2003 | X |  |
| Graham., et al.,1982 | X | X |
| Graham., et al.,1991 | X | X |
| Graham., et al.,1992 | X | X |
| Hermann., et al.,2002 | X |  |
| Hirohata., et al.,1985 | X | X |
| Holmberg., et al.,1994 | X | X |
| Holmes., et al.,2004 | X |  |
| Holmes., et al.,1999 | X | X |
| Horn-Ross., et al., 2002 | X |  |
| Howe., et al., 1991 |  | X |
| Hunter., et al., 1996 | X |  |
| Ingram., et al.,1991 |  | X |
| Jones., et al.,1987 | X | X |
| Katsouyanni., et al.,1988 |  | X |
| Katsouyanni., et al.,1994 |  | X |
| Kim., et al.,2006 | X |  |
| Knekt., et al.,1990 |  | X |
| Kushi., et al.,1992 | X | X |
| Landa., et al.,1994 |  | X |
| Lee., et al.,1991 | X | X |
| Levi., et al.,1993 |  | X |
| Linos., et al.,2010 | X |  |
| London., et al.,1993 | X |  |
| Luf., et al.,2007 | X |  |
| Mannisto., et al.,1999 | X | X |
| Martin-Moreno., et al.,1994 | X | X |
| Miller., et al.,1978 |  | X |
| Nunez., et al.,1996 |  | X |
| Potischman., et al. 1998, | X | X |
| Prentice., et al.,2006 | X |  |
| Pryor., et al.,1989 | X | X |
| Richardson., et al.,1991 | X | X |
| Rohan., et al.,1998 | X |  |
| Rohan., et al.,1988 |  | X |
| Ronco., et al.,1996 | X |  |
| Salaetal., et al.,2000 | X |  |
| Seiri., et al.,2002 | X |  |
| Sun-Zhang., et a1990l., | X | X |
| Thiebaut., et al.,2007 | X |  |
| Thiebaut., et al.,2001 |  | X |
| Toniolo., et al.,1994 |  | X |
| Toniolo., et al.,1989 | X | X |
| Trichopoulou., et al1995., | X | X |
| van den Brandt., et al.,1993 | X | X |
| Van’t Veer., et al.,1991 |  | X |
| Velie., et al.,2000 | X | X |
| Voorrips., et al.,2002 | X |  |
| Wakai., et al.,2000 | X | X |
| Wang., et al.,2008 | X |  |
| Willett., et al.,1992 | X |  |
| Willett., et al.,1987 | X |  |
| Wirfalt., et al.,2002 | X |  |
| Witte., et al.,1997 | X | X |
| Wolk., et al.,1998 | X | X |
| Yuan., et al.,1995 |  | X |
| Zaridze., et al.,1991 | X | X |
| N | 95 | |
| Rows (r) | 66 | |
| Columns (c) | 2 | |
| Corrected Covered Area= (N-r)/rc-r | 43.9 % very high CCA. *This article was selected due to the higher quality according to AMSTAR-2 tool. | |

**(11)**

|  | *Li. et al.,2022** | *Farvid. et al., 2020* |
| --- | --- | --- |
| Primary studies |  |  |
| Andrea., et al., 2021 | X |  |
| Cade., et al.,2007 |  | X |
| Deschasaux., et al., 2013 | X | X |
| Elizabeth., et al.,2007 | X |  |
| Farvid., et al., 2016 | X | X |
| Ferrari., et al.,2013 | X | X |
| Giles., et al., 2006 | X | X |
| Graham., et al.,1992 | X | X |
| Heath., et al.,2020 | X |  |
| Holmes., et al., 2004 |  | X |
| Horn-Ross., et al., 2002 |  | X |
| Key., et al., 2019 | X | X |
| Kushi., et al.,1992 | X | X |
| Maruti., et al.,2008 |  | X |
| Maruti., et al.,2000 | X |  |
| Narita., et al.,2017 | X | X |
| Park., et al.,2009 | X | X |
| Shikany., et al.,2011 | X | X |
| Sieri., et al.,2002 | X | X |
| Sonestedt., et al.2008, | X |  |
| Suzuki,., et al.,2008 | X | X |
| Terry., et al.,2002 | X | X |
| Verhoeven., et al.1997, | X | X |
| Wen., et al.,2009 | X | X |
| N | 39 | |
| Rows (r) | 24 | |
| Columns (c) | 2 | |
| Corrected Covered Area= (N-r)/rc-r | 62.5% very high CCA. *This article was selected due to the higher quality according to AMSTAR-2 tool. | |

**(12)**

|  | *Cao. et al.,2016** | *Turati. et al., 2015* |
| --- | --- | --- |
| Primary studies |  |  |
| Augustin., et al., 2001 |  | X |
| Cho., et al.,2003 | X | X |
| Frazier., et al., 2004 | X |  |
| George., et al.,2009 |  | X |
| Giles., et al., 2006 | X |  |
| Higginbotham., et al.,2004 |  | X |
| Holmes., et al., 2004 | X | X |
| Hu., et al.,2013 |  | X |
| Jonas., et al.,2003 | X | X |
| Lajous., et al., 2008 | X |  |
| Lajous., et al., 2005 |  | X |
| Larsson., et al., 2009 | X | X |
| Levi., et al.,2002 |  | X |
| McCann., et al.,2007 |  | X |
| Nielsen., et al.,2005 | X | X |
| Romieu., et al.,2012 |  | X |
| Shikany., et al.,2011 | X | X |
| Sieri., et al.,2007 | X | X |
| Silvera., et al.,2005 | X | X |
| Wen., et al.,2009 | X | X |
| Woo., et al.,2013 |  | X |
| Yun., et al., 2010 |  | X |
| N | 31 | |
| Rows (r) | 22 | |
| Columns (c) | 2 | |
| Corrected Covered Area= (N-r)/rc-r | 40.9% very high CCA. *This article was selected due to the higher quality according to AMSTAR-2 tool. | |

**(13)**

|  | *Cao. et al.,2016** | *Turner. et al., 2011* |
| --- | --- | --- |
| Primary studies |  |  |
| Boeke., et al., 2014 | X |  |
| Byrne., et al.,2002 |  | X |
| Cho., et al., 2003 |  | X |
| Freedman., et al.,2006 | X |  |
| Gaard., et al., 1995 |  | X |
| Gago-Dominguez., et al., 2003 |  | X |
| Goodstine., et al.,2003 |  | X |
| Hermann., et al.,2002 |  | X |
| Holmes., et al.,1999 |  | X |
| Horn-Ross., et al.,2002 |  | X |
| Howe., et al., 1991 | X |  |
| Hunter., et al.,1996 |  | X |
| Jones., et al.,1987 | X |  |
| Katsouyanni., et al.,1994 |  | X |
| Kim., et al., 2006 |  | X |
| Knekt., et al., 1990 | X |  |
| Kushi., et al.,1995 | X |  |
| Kushi., et al.,1992 |  | X |
| Lee., et al.,1991 |  | X |
| Linos., et al.,2010 |  | X |
| Löf., et al.,2007 | X |  |
| London., et al.,1993 |  | X |
| Luf., et al.,2007 |  | X |
| Park., et al.,2012 | X |  |
| Richardson., et al.,1991 |  | X |
| Saadatian-Elahi., et al.,2004 |  | X |
| Sczaniecka., et al.,2012 | X |  |
| Seiri., et al.,2002 | X | X |
| Seiri., et al.,2014 | X |  |
| Thiébaut., et al.,2007 | X | X |
| Toniolo., et al.,1994 |  | X |
| Velie., et al.,2000 |  | X |
| Voorrips., et al.,2002 | X | X |
| Wakai., et al.,2005 | X |  |
| Wirfält., et al.,2002 | X | X |
| Wolk., et al.,1998 | X |  |
| N | 40 | |
| Rows (r) | 36 | |
| Columns (c) | 2 | |
| Corrected Covered Area= (N-r)/rc-r | 11.1% high CCA. *This article was selected due to the higher quality according to AMSTAR-2 tool. | |

**(14)-processed meat intake**

|  | *Farvid. et al.,2021** | *Anderson. et al., 2018* |
| --- | --- | --- |
| Primary studies |  |  |
| Anderson., et al., 2017 |  | X |
| Cross., et al.,2007 |  | X |
| Diallo., et al., 2018 | X |  |
| Dunneram., et al.,2019 | X |  |
| Farvid., et al., 2014 | X |  |
| Ferucci., et al.,2009 | X | X |
| Genkinger., et al., 2013 | X | X |
| Gilsing., et al.,2016 | X |  |
| Holmes., et al.,2003 | X | X |
| Inoue-Choi., et al., 2016 | X |  |
| Key., et al., 1999 | X |  |
| Knuppel., et al., 2020 | X |  |
| Larsson., et al.,2009 | X |  |
| Lo., et al.,2019 | X |  |
| Marcondes., et al.,2019 | X |  |
| Pala., et al.,2009 | X | X |
| Pouchieu., et al.,2014 | X | X |
| Taylor., et al.,2007 |  | X |
| VanDer Heletal.., et al.,2004 | X |  |
| N | 24 | |
| Rows (r) | 19 | |
| Columns (c) | 2 | |
| Corrected Covered Area= (N-r)/rc-r | 26.3% very high CCA. *This article was selected due to the higher quality according to AMSTAR-2 tool. | |

**(15)**

|  | *Li. et al.,2022** | *Xiao. et al., 2018* |
| --- | --- | --- |
| Primary studies |  |  |
| Adzersen., et al., 2003 |  | X |
| Andrea., et al.,2021 | X |  |
| Chatenoud., et al., 1998 |  | X |
| Dunneram., et al.,2019 | X |  |
| Egeberg., et al., 2009 | X | X |
| Farvid., et al.,2016 | X | X |
| Hyun., et al., 2017 | X |  |
| LaVecchia., et al.,1987 |  | X |
| Levi., et al.,1993 |  | X |
| Makarem., et al.,2018 | X |  |
| Mourouti., et al., 2016 |  | X |
| Nicodemus., et al., 2001 | X | X |
| Sonestedt., et al.,2008 | X | X |
| Tajaddini., et al.,2015 |  | X |
| Yun., et al.,2010 |  | X |
| N | 19 | |
| Rows (r) | 15 | |
| Columns (c) | 2 | |
| Corrected Covered Area= (N-r)/rc-r | 26.7% very high CCA. *This article was selected due to the higher quality according to AMSTAR-2 tool. | |

**(16)**

|  | *Wong. et al.,2021** | *Chen. et al., 2018* |
| --- | --- | --- |
| Primary studies |  |  |
| Angel., et al., 2020 | X |  |
| Cao., et al.,2018 | X |  |
| Girschik., et al., 2013 |  | X |
| Gu., et al.,2016 | X | X |
| Hurley., et al., 2015 | X | X |
| Kakizaki., et al.,2008 | X | X |
| Li., et al., 2011 | X |  |
| Lie., et al.,2006 | X |  |
| Lie., et al.,2011 | X |  |
| McElroy., et al.,2006 |  | X |
| McNeil., et al., 2019 | X |  |
| O’Leary., et al., 2006 | X |  |
| Pesch., et al.,2010 | X |  |
| Pinheiro., et al.,2006 | X | X |
| Pronk., et al.,2010 | X |  |
| Qian., et al.,2015 | X | X |
| Schernhammer., et al.,2001 | X |  |
| Schernhammer., et al.,2006 | X |  |
| Shen., et al.,2019 | X |  |
| Shigesato., et al.,2020 | X |  |
| Tynes., et al., 1996 | X |  |
| Verkasalo., et al., 2005 | X | X |
| Vogtmann., et al., 2013 | X | X |
| Wang., et al., 2015 |  | X |
| White., et al., 2017 | X |  |
| Wu., et al., 2013 | X | X |
| Xiao., et al., 2016 | X | X |
| N | 36 | |
| Rows (r) | 27 | |
| Columns (c) | 2 | |
| Corrected Covered Area= (N-r)/rc-r | 33.3% very high CCA. *This article was selected due to the higher quality according to AMSTAR-2 tool. | |

**(17)**

|  | *Wei. et al.,2021* | *Van. et al., 2021** |
| --- | --- | --- |
| Primary studies |  |  |
| Akerstedt., et al., 2015 | X | X |
| Andersen., et al.,2018 | X |  |
| Bustamante-Montes., et al.,2019 |  | X |
| Davis., et al.,2001 |  | X |
| Fritschi., et al., 2013 |  | X |
| Grundy., et al.,2013 |  | X |
| Hansen., et al., 2001 |  | X |
| Hansen and Lassen., et al.,2012 |  | X |
| Harris., et al.,2020 |  | X |
| James., et al., 2017 | X |  |
| Jones., et al., 2019 | X | X |
| Jorgensen., et al., 2017 |  | X |
| Knutsson., et al.,2013 | X | X |
| Koppes., et al.,2014 | X | X |
| Li., et al.,2014 |  | X |
| Lie., et al.,2006 |  | X |
| Lie., et al.,2011 |  | X |
| McNeil., et al.,2020 |  | X |
| Menegaux., et al.,2013 |  | X |
| O'Leary., et al.,2006 |  | X |
| Papantoniou., et al.,2016 |  | X |
| Pesch., et al.,2010 |  | X |
| Pham., et al.,2019 |  | X |
| Pronk., et al.,2010 | X | X |
| Schernhammer., et al.,2001 | X |  |
| Schernhammer., et al.,2006 | X |  |
| Sweeney., et al.,2020 | X | X |
| Szkiela., et al.,2021 |  | X |
| Travis., et al.,2016 | X | X |
| Vistisen., et al.,2017 | X | X |
| Wang., et al.,2015 |  | X |
| Wegrzyn., et al.,2017 | X | X |
| White., et al.,2017 | X |  |
| Yang., et al., 2019 |  | X |
| N | 43 | |
| Rows (r) | 34 | |
| Columns (c) | 2 | |
| Corrected Covered Area= (N-r)/rc-r | 26.5% very high CCA. *This article was selected due to the same quality according to AMSTAR-2 tool but of recent time. | |

**(18)**

|  | Zhao*. et al.,2014** | *Chen. et al., 2013* |
| --- | --- | --- |
| Primary studies |  |  |
| Coogan., et al., 1996 |  | X |
| Coogan., et al.,1998 | X | X |
| Davis., et al., 2002 | X | X |
| Demers., et al.,1991 | X |  |
| Feychting., et al., 1998 | X | X |
| Forssen., et al.,2000 | X | X |
| Forssen., et al., 2005 |  | X |
| Gammon., et al.,1998 |  | X |
| Kabab., et al.,2003 |  | X |
| Kabat., et al.,2003 | X | X |
| Kliukiene., et al., 2003 |  | X |
| Kliukiene., et al., 2004 | X | X |
| Labreche., et al.,2003 | X | X |
| Li., et al.,1997 |  | X |
| London., et al.,2003 | X | X |
| Loomis., et al.,1994 | X | X |
| McElroy., et al.,2001 | X | X |
| McElroy., et al.,2007 | X | X |
| Rosenbaum., et al.,1994 | X |  |
| Schoenfeld., et al.,2003 | X | X |
| Wijngaardem., et al.,2001 | X | X |
| Vena., et al.,1994 |  | X |
| Vena., et al.,1991 |  | X |
| Zheng., et al.,2000 |  | X |
| Zhu., et al., 2003 | X | X |
| N | 39 | |
| Rows (r) | 25 | |
| Columns (c) | 2 | |
| Corrected Covered Area= (N-r)/rc-r | 56% very high CCA. *This article was selected due to the same quality according to AMSTAR-2 tool but of recent time. | |

**(19)**

|  | *Jin. et al.,2020** | *Farahmand. et al., 2019* |
| --- | --- | --- |
| Primary studies |  |  |
| Afshar., et al., 2015 |  | X |
| Antonsson., et al.,2012 |  | X |
| El-Naby., et al., 2017 | X | X |
| Fawzy., et al.,2008 | X | X |
| Fessahaye., et al., 2017 | X | X |
| Fina., et al.,2001 |  | X |
| Glenn., et al., 2012 |  | X |
| Hachana., et al.,2011 | X | X |
| Joshi., et al.,2009 | X | X |
| Kalkan., et al., 2005 | X | X |
| Kazemi Aghdam., et al., 2017 |  | X |
| Khabaz., et al., 2013 | X | X |
| Labrecque., et al.,1995 |  | X |
| Ladera., et al.,2017 |  | X |
| Lorenzetti., et al.,2010 |  | X |
| Mazouni., et al.,2011 |  | X |
| Mohamed., et al.,2007 |  | X |
| Mohammadizadeh., et al.,2014 | X |  |
| Morales-Sánchez., et al.,2013 | X | X |
| Naushad., et al.,2017 |  | X |
| Pai., et al.,2018 | X | X |
| Peng., et al.,2014 | X |  |
| Preciado., et al.,2005 | X | X |
| Richardson., et al.,2015 | X | X |
| Saeedi., et al.,2018 |  | X |
| Shadood., et al.,2018 |  | X |
| Sharifpour., et al.,2019 |  | X |
| Tahmasebi Fard., et al., 2013 |  | X |
| Tsai., et al.,2005 |  | X |
| Yahia., et al.,2014 | X | X |
| Zekri., et al.,2012 | X | X |
| Zhang., et al.,2017 |  | X |
| N | 45 | |
| Rows (r) | 32 | |
| Columns (c) | 2 | |
| Corrected Covered Area= (N-r)/rc-r | 40.6% very high CCA. *This article was selected due to the higher quality according to AMSTAR-2 tool. | |

**Supplemental Table 9.** Mendelian randomization (MR) analysis; exposure-outcome included in the main analysis, cohorts only.

|  | **Exposure** | **Example of Exposure contras** | **Outcome** | **MR** | **F-statistics** |
| --- | --- | --- | --- | --- | --- |
|  | | | | |  |
| 1 | Breast density | highest vs. lowest category | Breast cancer | No GWAS available | NA |
| 2 | Cardiac glycosides use | ever vs. never | Breast cancer | Insufficient SNPs | NA |
| 3 | Atrial fibrillation | ever vs. never | Breast cancer | OK | 156.27 |
| 5 | Vegetable-fruit-soybean dietary pattern | highest vs. lowest | Breast cancer | No GWAS available | NA |
| 6 | BMI>=25 | high vs. low, postmenopausal | Breast cancer | Insufficient SNPs | NA |
| 7 | Antipsychotic use | ever vs. never | Breast cancer | No GWAS available | NA |
| 8 | Calcium channel blockers | ever vs. never | Breast cancer | OK | 19.21 |
| 9 | Education level | highest vs. lowest category | Breast cancer | OK | 13.75 |
| 10 | Antibiotic use | ever vs. never | Breast cancer | No GWAS available | NA |
| 11 | Light exposure at night | highest vs. lowest category | Breast cancer | No GWAS available | NA |
| 12 | Smoking | ever vs. never | Breast cancer | OK | 34.74 |
| 13 | Physical activity | highest vs. lowest category | Breast cancer | OK | 199.28 |
| 14 | BMI iya, dose-response | per 5 kg/m2 | Breast cancer | No GWAS available | NA |
| 15 | Antibody | ever vs. never | Breast cancer | No GWAS available | NA |
| 16 | Alcohol | highest vs. lowest category | Breast cancer | OK | 27.96 |
| 17 | Bone mineral density | highest vs. lowest category | Breast cancer | OK | 14.32 |
| 18 | Fat mass | highest vs. lowest category | Breast cancer | OK | 12.61 |
| 19 | Weight gain | highest vs. lowest category | Breast cancer | No GWAS available | NA |
| 20 | Famine exposure | ever vs. never | Breast cancer | No GWAS available | NA |
| 21 | IGF-1 concentrations | highest vs. lowest category | Breast cancer | OK | 16.53 |
| 22 | Periodontal disease | ever vs. never | Breast cancer | No GWAS available | NA |
| 23 | BMI | highest vs. lowest category | Breast cancer | OK | 13.61 |
| 24 | Metabolic Syndrome | ever vs. never | Breast cancer | No GWAS available | NA |
| 25 | Hyperthyroidism | ever vs. never | Breast cancer | Weak instrument | 4.01 |
| 26 | Total meat intake, dose-response | per 100 g/day | Breast cancer | No GWAS available | NA |
| 27 | Red meat intake | highest vs. lowest category | Breast cancer | No GWAS available | NA |
| 28 | Fruit intake, dose-response | per 100 g/day | Breast cancer | OK | 50.95 |
| 29 | BMI, dose-response | per 5 kg/m2 | Breast cancer | No GWAS available | NA |
| 30 | Aspirin intake | ever vs. never | Breast cancer | OK | 197.23 |
| 31 | Fiber intake | highest vs. lowest category | Breast cancer | No GWAS available | NA |
| 32 | Selenium | ever vs. never | Breast cancer | No GWAS available | NA |
| 33 | BMI<25 | high vs. low, postmenopausal | Breast cancer | No GWAS available | NA |
| 34 | Lifestyle Quality Indices | highest vs. lowest category | Breast cancer | No GWAS available | NA |
| 35 | Tofu intake | highest vs. lowest category | Breast cancer | OK | 42.46 |
| 36 | Adherence score | highest vs. lowest category | Breast cancer | No GWAS available | NA |
| 37 | Bariatric Surgery | ever vs. never | Breast cancer | No GWAS available | NA |
| 38 | Autoimmune thyroiditis | ever vs. never | Breast cancer | No GWAS available | NA |
| 39 | Goitre | ever vs. never | Breast cancer | OK | 292.05 |
| 40 | Negative Emotions | ever vs. never | Breast cancer | No GWAS available | NA |
| 41 | Birth length | highest vs. lowest category | Breast cancer | No GWAS available | NA |
| 42 | Obstructive sleep apnea | ever vs. never | Breast cancer | No GWAS available | NA |
| 43 | Flight attendants | yes vs. no | Breast cancer | No GWAS available | NA |
| 44 | Serum/plasma iron | highest vs. lowest category | Breast cancer | No GWAS available | NA |
| 45 | Sleep-disordered breathing | ever vs. never | Breast cancer | Insufficient SNPs | NA |
| 46 | DII, dose-response | per 1‐point | Breast cancer | No GWAS available | NA |
| 47 | Wine Drinking | highest vs. lowest category | Breast cancer | Insufficient SNPs | NA |
| 48 | BMI>=30 | highest vs. lowest category | Breast cancer | No GWAS available | NA |
| 49 | Processed meat intake, dose-response | per 50 g/day | Breast cancer | OK | 110.38 |
| 50 | Plasma prolactin levels | highest vs. lowest category | Breast cancer | No GWAS available | NA |
| 51 | SSBs, dose-response | per 250mg/day | Breast cancer | No GWAS available | NA |
| 52 | Diabetes | ever vs. never | Breast cancer | OK | 48.06 |
| 53 | Sedentary work | yes vs. no | Breast cancer | No GWAS available | NA |
| 54 | Birth Weight | highest vs. lowest category | Breast cancer | OK | 16.16 |
| 55 | Occupational exposure-organic solvents | ever vs. never | Breast cancer | No GWAS available | NA |
| 56 | Total fat intake | highest vs. lowest category | Breast cancer | Insufficient SNPs | NA |
| 57 | Antidepressant use | ever vs. never | Breast cancer | Insufficient SNPs | NA |
| 58 | Paternal age, dose-response | per 15 years | Breast cancer | No GWAS available | NA |
| 59 | Processed meat intake | highest vs. lowest category | Breast cancer | No GWAS available | NA |
| 60 | Glycemic index/Glycemic load | highest vs. lowest category | Breast cancer | No GWAS available | NA |
| 61 | Glycemic index | highest vs. lowest category | Breast cancer | No GWAS available | NA |
| 62 | Glycemic index, dose-response | per 10 units/day | Breast cancer | No GWAS available | NA |
| 63 | NO2, dose-response | per 10 ug/m3 | Breast cancer | No GWAS available | NA |
| 64 | Vegetable intake, dose-response | Per 100 g/day | Breast cancer | No GWAS available | NA |
| 65 | Soy intake, dose-response | per 30 g/day | Breast cancer | No GWAS available | NA |
| 66 | Soy isoflavone, dose-response | per 10mg/day | Breast cancer | No GWAS available | NA |
| 67 | Coffee intake | per 10mg/day | Breast cancer | OK | 73.20 |
| 68 | Vitamin D intake | highest vs. lowest category | Breast cancer | No GWAS available | NA |
| 69 | Cheese intake, dose-response | per 30 g/day | Breast cancer | OK | 37.50 |
| 70 | B-carotene, dose-response | per 5000ug/day | Breast cancer | No GWAS available | NA |
| 71 | Fruit intake | highest vs. lowest category | Breast cancer | No GWAS available | NA |
| 72 | Serum TG levels | highest vs. lowest category | Breast cancer | OK | 21.61 |
| 73 | Flavonols | per 5000ug/day | Breast cancer | No GWAS available | NA |
| 74 | Dietary calcium intake, dose-response | per 350mg/day | Breast cancer | No GWAS available | NA |
| 75 | Dietary folate intake | highest vs. lowest category | Breast cancer | OK | 930.44 |
| 76 | Prudent/healthy dietary pattern | highest vs. lowest category | Breast cancer | No GWAS available | NA |
| 77 | Twin membership | highest vs. lowest category | Breast cancer | No GWAS available | NA |
| 78 | Adherence score, dose-response | per 1‐point | Breast cancer | No GWAS available | NA |
| 79 | A-carotene | highest vs. lowest category | Breast cancer | Repeated | NA |
| 80 | Parity | parous vs. nulliparous | Breast cancer | No GWAS available | NA |
| 81 | Vitamin B2 | highest vs. lowest category | Breast cancer | No GWAS available | NA |
| 82 | Weight loss | highest vs. lowest category | Breast cancer | No GWAS available | NA |
| 83 | Fruits and vegetables intake | highest vs. lowest category | Breast cancer | No GWAS available | NA |
| 84 | Bisphosphonates | ever vs. never | Breast cancer | No GWAS available | NA |
| 85 | Physical activity at a young age | highest vs. lowest category | Breast cancer | No GWAS available | NA |
| 86 | Thiazolidinediones | ever vs. never | Breast cancer | No GWAS available | NA |
| 87 | Total dairy food intake | highest vs. lowest category | Breast cancer | No GWAS available | NA |
| 88 | Dietary calcium intake | highest vs. lowest category | Breast cancer | Repeated | NA |
| 89 | CAD | ever vs. never | Breast cancer | EAF deficiency | NA |
| 90 | Insulins | ever vs. never | Breast cancer | OK | 590.76 |
| 91 | Vegetarians | yes vs. no | Breast cancer | Insufficient SNPs | NA |
| 92 | Higher Mushroom Consumption | highest vs. lowest category | Breast cancer | No GWAS available | NA |
| 93 | Time in the Sun | highest vs. lowest category | Breast cancer | No GWAS available | NA |

**Supplemental Table 10.** Two-sample inverse variance weighted mendelian randomization full results of the analyses of risk factors on breast cancer.

| Exposure | OR | SE | Lower 95%CI | Upper 95%CI | P | FDR |
| --- | --- | --- | --- | --- | --- | --- |
| Atrial fibrillation | 3.32 | 0.71 | 0.82 | 13.45 | 0.092 | 0.19883 |
| Calcium channel blockers | 0.91 | 0.03 | 0.86 | 0.98 | 0.009 | 0.04314 |
| Education level | 0.99 | 0.01 | 0.96 | 1.01 | 0.185 | 0.29129 |
| Smoking | 1.18 | 0.10 | 0.97 | 1.43 | 0.095 | 0.19883 |
| Physical activity | 0.94 | 0.03 | 0.88 | 1.01 | 0.088 | 0.19883 |
| Alcohol | 1.01 | 0.06 | 0.91 | 1.13 | 0.823 | 0.82331 |
| Bone mineral density | 1.01 | 0.03 | 0.96 | 1.06 | 0.716 | 0.82331 |
| Fat mass | 0.80 | 0.12 | 0.63 | 1.01 | 0.058 | 0.18349 |
| IGF-1 concentrations | 1.08 | 0.03 | 1.02 | 1.15 | 0.009 | 0.04314 |
| BMI | 0.82 | 0.07 | 0.71 | 0.95 | 0.009 | 0.04314 |
| Fruit intake | 0.64 | 0.17 | 0.46 | 0.90 | 0.010 | 0.04314 |
| Aspirin intake | 0.84 | 0.71 | 0.21 | 3.41 | 0.808 | 0.82331 |
| Tofu intake | 1.41 | 0.86 | 0.26 | 7.58 | 0.690 | 0.82331 |
| Goitre | 28.76 | 4.99 | 0.00 | 509785.17 | 0.501 | 0.73478 |
| Processed meat intake | 1.28 | 0.17 | 0.93 | 1.78 | 0.133 | 0.24322 |
| Diabetes | 0.71 | 0.60 | 0.22 | 2.30 | 0.567 | 0.77994 |
| Birth Weight | 1.10 | 0.06 | 0.98 | 1.24 | 0.099 | 0.19883 |
| Coffee intake | 0.77 | 0.18 | 0.54 | 1.10 | 0.156 | 0.26372 |
| Cheese intake | 0.82 | 0.09 | 0.69 | 0.98 | 0.032 | 0.11790 |
| Serum TG levels | 0.91 | 0.03 | 0.86 | 0.97 | 0.005 | 0.04314 |
| Dietary folate intake | 0.30 | 3.07 | 0.00 | 122.52 | 0.693 | 0.82331 |
| Insulins | 0.59 | 2.23 | 0.01 | 47.23 | 0.816 | 0.82331 |

**Abbreviations:** FDR: false discovery rate; OR: odds ratio; P: P-value; SE: standard error; 95%CI: 95% confidence interval.

**Supplemental Table 11.** Results from Mendelian randomization sensitivity analyses of risk factors on breast cancer.

|  | **Weighted Median** | | | | **MR-Egger** | | | | **MR-Pleiotropy test** |
| --- | --- | --- | --- | --- | --- | --- | --- | --- | --- |
|  | **OR** | **CI-lower** | **CI-upper** | **P-value** | **OR** | **CI-lower** | **CI-upper** | **P-value** | **P-value** |
| Atrial fibrillation | 0.83 | 0.11 | 6.06 | 0.857 | 0.27 | 0.01 | 5.21 | 0.401 | 0.092 |
| Calcium channel blockers | 0.98 | 0.94 | 1.03 | 0.515 | 0.81 | 0.66 | 1.00 | 0.049 | 0.009 |
| Education level | 0.99 | 0.97 | 1.01 | 0.377 | 0.97 | 0.88 | 1.08 | 0.609 | 0.185 |
| Smoking | 1.06 | 0.87 | 1.30 | 0.559 | 1.44 | 0.64 | 3.25 | 0.386 | 0.095 |
| Physical activity | 0.97 | 0.93 | 1.01 | 0.126 | 0.99 | 0.63 | 1.55 | 0.962 | 0.088 |
| Alcohol | 1.04 | 0.93 | 1.17 | 0.479 | 1.03 | 0.82 | 1.28 | 0.813 | 0.823 |
| Bone mineral density | 1.01 | 0.95 | 1.06 | 0.792 | 1.01 | 0.88 | 1.16 | 0.885 | 0.716 |
| Fat mass | 0.74 | 0.64 | 0.87 | 0.000 | 0.30 | 0.16 | 0.56 | 0.000 | 0.058 |
| IGF-1 concentrations | 1.07 | 1.00 | 1.14 | 0.053 | 1.07 | 0.94 | 1.21 | 0.319 | 0.009 |
| BMI | 0.75 | 0.66 | 0.85 | 0.000 | 0.43 | 0.31 | 0.60 | 0.000 | 0.009 |
| Fruit intake | 0.66 | 0.46 | 0.94 | 0.022 | 0.37 | 0.12 | 1.16 | 0.094 | 0.010 |
| Aspirin intake | 2.21 | 0.68 | 7.18 | 0.187 | 0.11 | 0.00 | 6.26 | 0.321 | 0.808 |
| Tofu intake | 1.99 | 0.28 | 13.95 | 0.49 | 31.92 | 0.04 | 23259.84 | 0.41 | 0.692 |
| Goitre | 0.87 | 0.00 | 19145.74 | 0.979 | 0.00 | 0.00 | 277178.04 | 0.392 | 0.501 |
| Processed meat intake | 1.27 | 0.92 | 1.77 | 0.152 | 0.41 | 0.08 | 2.08 | 0.296 | 0.133 |
| Diabetes | 2.08 | 0.75 | 5.81 | 0.162 | 7.71 | 0.43 | 139.44 | 0.172 | 0.567 |
| Birth Weight | 1.12 | 1.01 | 1.24 | 0.035 | 0.85 | 0.58 | 1.26 | 0.431 | 0.099 |
| Coffee intake | 0.92 | 0.70 | 1.21 | 0.551 | 1.00 | 0.49 | 2.04 | 0.989 | 0.156 |
| Cheese intake | 0.89 | 0.74 | 1.08 | 0.229 | 0.85 | 0.41 | 1.78 | 0.673 | 0.032 |
| Serum TG levels | 0.92 | 0.87 | 0.97 | 0.004 | 0.89 | 0.82 | 0.98 | 0.016 | 0.005 |
| Dietary folate intake | 0.27 | 0.00 | 14.82 | 0.518 | 0.00 | 0.00 | 0.02 | 0.286 | 0.693 |
| Insulins | 0.10 | 0.02 | 0.55 | 0.008 | 0.17 | 0.00 | 821.31 | 0.694 | 0.816 |

**Supplemental Table 12.** Results from multivariable (MV) Mendelian randomization (MR) sensitivity analyses of risk factors on breast cancer.

**Table12.1.**

|  | **OR** | **SE** | **95%CI-lower** | **95%CI-upper** | **P-value** |
| --- | --- | --- | --- | --- | --- |
| **Calcium channel blockers** | 0.920 | 0.040 | 0.850 | 0.995 | 0.033 |
| **IGF-1 concentrations** | 1.083 | 0.030 | 1.021 | 1.148 | 0.000 |
| **BMI** | 0.954 | 0.049 | 0.867 | 1.050 | 0.162 |
| **Serum TG levels** | 0.978 | 0.034 | 0.915 | 1.045 | 0.367 |

**Table 12.2.**

|  | **OR** | **SE** | **95%CI-lower** | **95%CI-upper** | **P-value** |
| --- | --- | --- | --- | --- | --- |
| **Fruit intake** | 0.776 | 0.096 | 0.643 | 0.935 | 0.008 |
| **Cheese intake** | 0.626 | 0.173 | 0.447 | 0.878 | 0.007 |

*Instruments selected based on exposure.
